# Entropy-Driven Design of Depolymerizable Polyolefins from Strained Bridged Bicyclic Monomers

**DOI:** 10.1021/jacs.6c02456

**Published:** 2026-03-25

**Authors:** Tarek Ibrahim, Desmond Brown, Hao Sun

**Affiliations:** Department of Chemistry and Chemical & Biomedical Engineering, Tagliatela College of Engineering, 8518 University of New Haven, West Haven, Connecticut 06516, United States

## Abstract

Polymers produced by ring-opening metathesis polymerization (ROMP) of strained cyclic olefin monomers, such as norbornene and cyclobutene, are challenging to depolymerize back to their constituent monomers due to their favorable polymerization thermodynamics. Current strategies for creating depolymerizable ROMP polymers focus on designing low-strain monomers with small enthalpic driving forces, which facilitate depolymerization by reducing the monomer polymerizability. Because polymerization thermodynamics is governed by both enthalpic and entropic contributions, we reason that depolymerizable polymers could be achieved from highly strained cyclic olefin monomers if the entropic penalty of polymerization is sufficiently large. Here, we present a depolymerizable polymer system based on a series of strained bicyclo[3.2.1] monomers, which combine a substantial enthalpic driving force (−6 to −11 kcal/mol) with a significant entropic penalty of polymerization (−15 to −24 cal/mol/K). The large entropic penalty, arising from the rigid polymer backbone, lowers the ceiling temperature and imparts depolymerizability to the polymer system, leading to monomer recovery (74–99%) under standard ring-closing metathesis conditions. Moreover, the enthalpic driving force remains sufficient to enable efficient ring-opening metathesis polymerization and block copolymer synthesis. This entropy-driven strategy thus unlocks access to depolymerizable polymers from strained cyclic olefin monomers that are not traditionally considered building blocks for such materials, offering a new direction for the design of chemically recyclable polymers with an expanded monomer scope.

## Graphical abstract

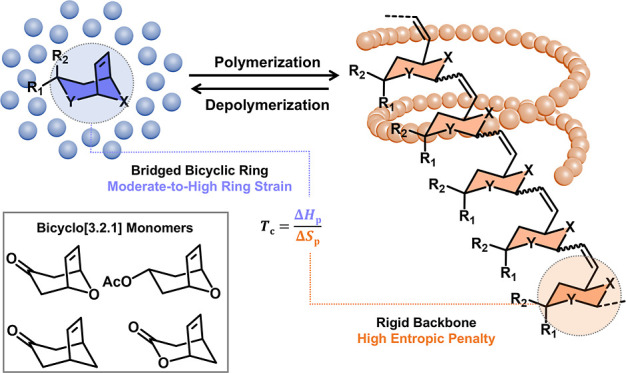

## Introduction

Chemical recycling to monomers via depolymerization offers a promising strategy to alleviate the global environmental burden of commodity plastic waste by enabling a circular plastic economy.^1–3^ Typically, depolymerization can be triggered by solvolysis or thermolysis. Solvolytic depolymerization is commonly used for polymer materials containing labile covalent bonds,^4,5^ whereas thermolytic depolymerization can be applied to polymers with hydrocarbon backbones, such as polyolefins.^6,7^ In thermally triggered depolymerization, polymers are heated above their ceiling temperature (*T*_c_), beyond which they revert to their constituent monomers.^8^ However, the inherently high ceiling temperatures of current commodity plastics (e.g., polyethylene, *T*_c_ = 610 °C) not only compromise the energy efficiency of the depolymerization process, but also lead to undesired side reactions at temperatures above *T*_c_.^8,9^ Therefore, there is strong motivation to develop new polymer structures with intrinsically low ceiling temperatures that enable energy-efficient and selective depolymerization under mild conditions.^10–13^ Moreover, for practical applications, it is critical that such low-ceiling-temperature polymers remain thermally stable in a kinetically trapped state and undergo depolymerization only in the presence of suitable catalysts.^14–17^

Ring-opening metathesis polymerization (ROMP) has been widely employed to access functional polymers, including the industrial production of polyolefins such as polynorbornenes (Norsorex) and polyoctenamers (Vestenamer).^18,19^ In a typical ROMP process, the release of ring strain energy (RSE) from cyclic olefin monomers provides the enthalpic driving force (Δ*H*_p_), which offsets the unfavorable entropy change (Δ*S*_p_ < 0) associated with the polymerization.^20^ Highly strained monomers (RSE >16 kcal/mol), such as norbornene, cyclopropene, and cyclobutene, are ideal candidates for ROMP due to their favorable polymerization thermodynamics (Figures 1a and S1).^21^ Nevertheless, polyolefins derived from these high-RSE monomers are generally nondepolymerizable because of their extremely high ceiling temperatures (Figure S1).^21^ To access ROMP polymers with lower *T*_c_ values, recent studies have focused on the design of low-RSE monomers (RSE = 3.8–7.2 kcal/mol) (Figure 1a), including 2,3-dihydrofuran,^22^ cyclopentene,^23,24^ dicyclopentadiene,^25^ substituted cyclohexene,^26^ fused-ring cyclohexenes,^27,28^ functional cycloheptenes,^29,30^ fused-ring cyclooctenes,^31–33^ (*Z*)–1-(*tert*-butyl)­cyclooct-4-en-1-ol,^34^ and macrocycles.^35,36^ The reduced RSE decreases the polymerization enthalpy, thereby lowering the ceiling temperature, as described by the equation: *T*_c_ = Δ*H*_p_/Δ*S*_p_.^37^ While this “low-strain” approach has enabled the development of depolymerizable polymers, the reduced ring strain of these monomers inevitably compromises polymerization efficiency, often leading to undesired secondary metathesis reactions when the backbone olefins lack sufficient steric hindrance.^38,39^ Moreover, complex architectures such as block copolymers remain inaccessible with low-strain monomers.

**Figure 1:**
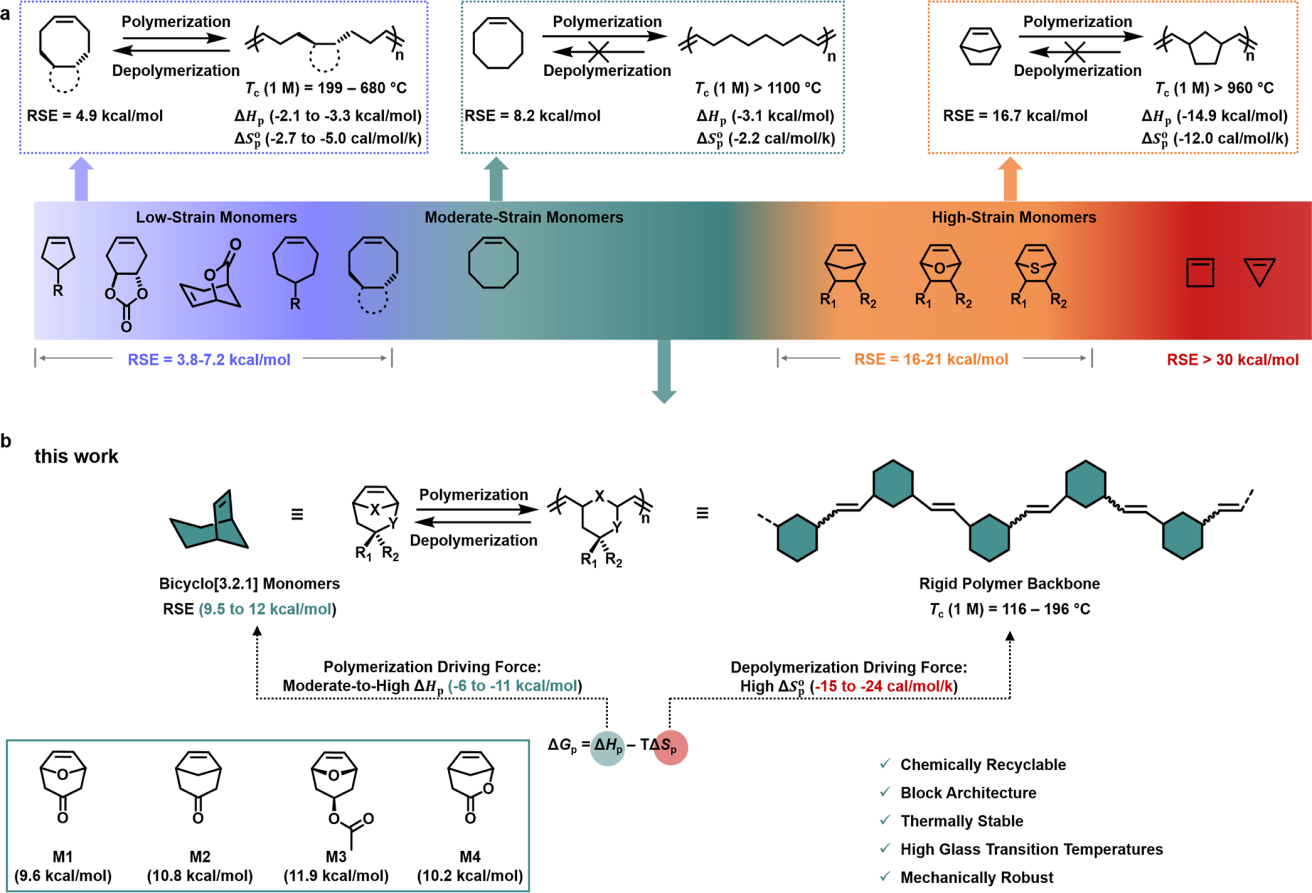
Reversibility in ROMP systems. (a) Reported relationship between monomer ring strain energies (RSEs) and their polymerization/depolymerization behavior. Low-strain monomers are typically used to access depolymerizable polymers, whereas polymers derived from conventional moderate-to high-RSE monomers are generally nondepolymerizable. (b) Entropy-driven design of depolymerizable polymers from strained bicyclo[3.2.1] monomers. The moderate-to high RSEs of bicyclo[3.2.1] monomers provide a moderate-to high enthalpic driving force for polymerization, while the large entropic penalty lowers the ceiling temperature, enabling efficient depolymerization.

Since the ceiling temperature of polymers is governed by both the enthalpy and entropy changes of polymerization (vide supra), we reason that low-*T*_c_, depolymerizable polyolefins can be obtained from moderate-to high-strain monomers, provided that the entropic penalty of polymerization is sufficiently large. Such low-*T*_c_ polymer systems would exhibit high polymerization efficiency due to their substantial enthalpic driving force, while also enabling efficient depolymerization driven by a large entropic gain. It is noteworthy that small-sized, low-strain cyclic olefin monomers such as cyclopentene and 2,3-dihydrofuran are characterized by relatively large entropic penalties of polymerization.^22,40^ However, their small enthalpy changes of polymerization, together with the large entropic penalties, render polymerization inefficient, often requiring low temperatures and high monomer concentrations to achieve satisfactory conversions.^38^ In contrast, the moderately strained, medium-sized *cis*-cyclooctene exhibits a minimal entropic penalty for polymerization, facilitating efficient polymerization but hindering depolymerization process.^21,31^ To date, a combination of a moderate-to-high enthalpic driving force and a large entropic penalty of polymerization is rare in ROMP. Because the substantial enthalpic driving force for polymerization disfavors the depolymerization process, depolymerization of ROMP polymers derived from moderately strained monomers can only be driven by a large entropic penalty of polymerization.

Notably, the overall entropy change of ROMP consists of a negative translational component (Δ*S*_t_), reflecting the loss of freedom as monomers are incorporated into the polymer chain, and a positive conformational component (Δ*S*_conf_), arising from the increased rotational and vibrational degrees of freedom along the polymer backbone.^31,41,42^ To make Δ*S*_p_ more negative, thereby increasing the entropic penalty, the gain in conformational entropy upon monomer ring opening can be reduced by limiting the number of freely rotatable bonds in the polymer backbone (Scheme S1). Consequently, a highly rigid backbone is desirable for designing low-*T*_c_ polymers, as it maximizes the entropic penalty of polymerization by minimizing Δ*S*_conf_.

## Results and Discussion

### Monomer Design and Synthesis

To access depolymerizable polymers that exhibit both large enthalpy and entropy changes of polymerization, we first turned our attention to norbornene derivatives, which are well-known for their substantial ring strain energies.^43–45^ The bridged bicyclic structure of norbornene allows ROMP of these monomers to produce polymers in which each repeating unit retains a five-membered ring, creating a rigid backbone that limits the conformational entropy gained during ring opening. Indeed, the entropy change in polymerization of norbornene (−12.0 cal/mol/K) is markedly larger than that of monocyclic monomers with similar ring sizes, such as cyclohexene (−7.4 cal/mol/K) and cyclooctene (−2.2 cal/mol/K) (Figure S1), supporting our rationale for selecting bridged bicyclic monomers to achieve a large entropic penalty of polymerization.^21^ However, norbornene also possesses an excessively large polymerization enthalpy (−14.9 kcal/mol), resulting in a ceiling temperature of 968 °C that remains too high for depolymerization (Figure 1a).^46^ It is noteworthy that the reported thermodynamic parameters for the ROMP of norbornene and other monocyclic olefin monomers shown in Figure S1 were determined using calorimetric methods, which differ from our van ’t Hoff approach (vide infra). Accordingly, the reported enthalpy and entropy values for these monomers should be considered relative rather than absolute.

To tune the thermodynamics toward lower ceiling temperatures, we designed a new class of bridged bicyclic monomers (**M1**–**M4**) based on a bicyclo[3.2.1] framework (Figure 1b). Compared with norbornene, a bicyclo[2.2.1] system, the additional atom in the bicyclo[3.2.1] ring is expected to reduce structural distortion and lower the ring strain energy (Figure 2). Furthermore, ring opening of bicyclo[3.2.1] monomers form larger, more sterically hindered six-membered rings than the five-membered rings in polynorbornene, generating a more rigid backbone and a potentially greater entropic penalty during polymerization (Figure 1b). Taken together, these thermodynamic considerations suggest that polymers derived from bicyclo[3.2.1] monomers would exhibit ceiling temperatures lower than that of polynorbornene, thus enabling efficient depolymerization unprecedented in traditional norbornene systems.

**Figure 2:**
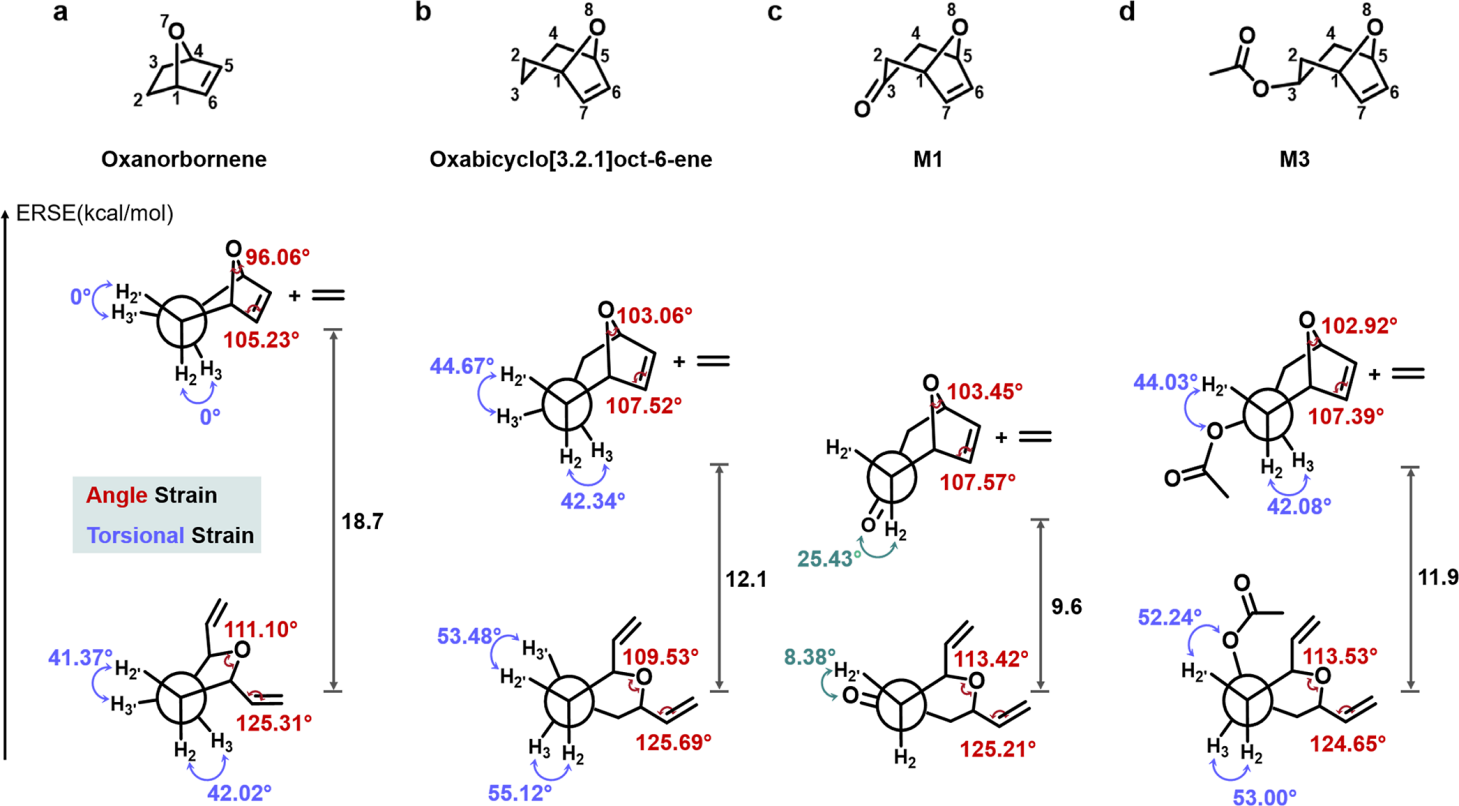
Conformational analysis of monomers and their ring-opened products. (a–d) Structures and corresponding Newman projections along the C2–C3 bond for oxanorbornene (a), oxabicyclo[3.2.1]­oct-6-ene (b), **M1** (c), and **M3** (d), along with their ethenolysis ring-opened products. Geometries and energies were optimized using density functional theory at the B3LYP/6–31G­(d) level. Both angle and torsional strains are dramatically reduced in the bicyclo[3.2.1] systems compared with oxanorbornene.

Monomers **M1**–**M3** were synthesized via [4 + 3] cycloaddition chemistry (Figures 3a and S2–S8). Specifically, **M1** was obtained from the cycloaddition of furan with 1,1,3,3-tetrabromoacetone,^47^ and served as a precursor for **M3** through the exoselective reduction of ketone and subsequent acetylation. **M2** was prepared using cyclopentadiene as the diene, while the lactone monomer **M4** was accessed via Baeyer–Villiger oxidation of norbornenone (Figures 3b and S9).

**Figure 3:**
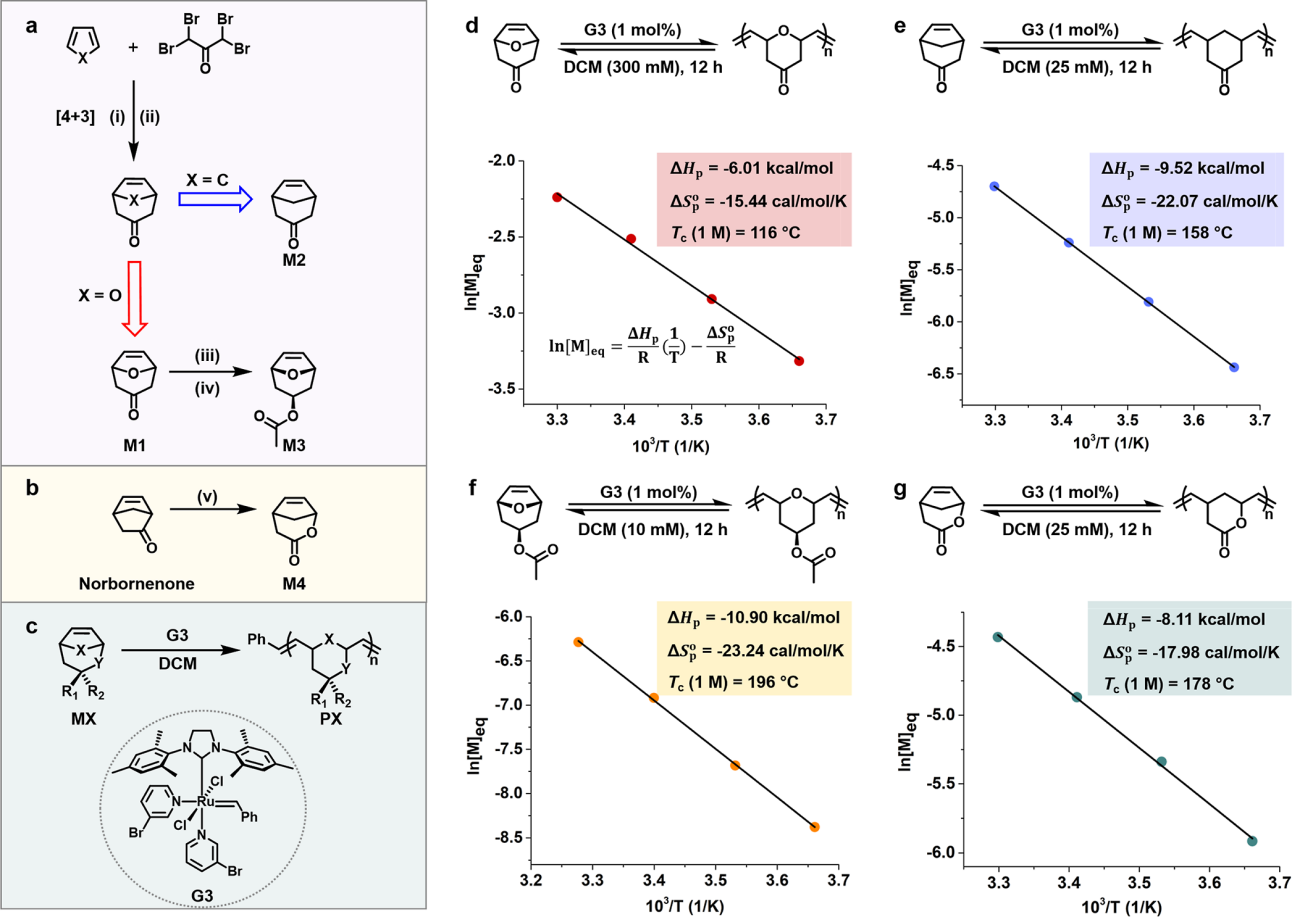
Synthesis of bicyclo[3.2.1] monomers and their polymerization thermodynamics. (a) Synthetic scheme of bicyclo[3.2.1] monomers **M1**–**M3**. Conditions: (i) Cu/Zn, MeCN, 5–10 °C; (ii) Cu/Zn, NH_4_Cl, −78 °C; (iii) Sm, diiodoethane, isopropanol, THF, reflux; (iv) acetic anhydride, TEA, 4-DMAP, 0–20 °C. (b) Synthetic route to **M4** via Baeyer–Villiger oxidation of norbornenone. Conditions: (v) mCPBA, NaHCO_3_, DCM, 0–10 °C. (c) ROMP synthesis of bicyclo[3.2.1]-based polymers using the third-generation Grubbs catalyst (**G3**) in DCM. (d) van ’t Hoff plot of **M1**, with the corresponding equation shown as an inset. (e–g) van ’t Hoff plots of **M2** (e), **M3** (f), and **M4** (g). The enthalpy change (Δ*H*_p_) and standard-state entropy change (Δ*S*_p_°) of the polymerization were obtained from the slope and y-intercept of each plot. The ceiling temperature at 1 M (*T*_c_ (1 M)) was calculated as the ratio of Δ*H*_p_ and Δ*S*_p_°.

### Computational Analysis of Bicyclo[3.2.1] Monomers

The RSEs of **M1**–**M4** were evaluated using density functional theory (DFT) calculations (Figures S68–S71) and compared with a library of previously reported ROMP monomers (Table S15 and Figures S72–S79). Conformational analysis of the monomers and their ring-opened forms revealed reductions in both angle and torsional strain relative to oxanorbornene (Figure 2). In particular, the insertion of an additional carbon mitigates the structural distortion inherent to oxanorbornene, as shown by the larger C1–O8–C5 bond angle in oxabicyclo[3.2.1]­oct-6-ene (103.06°) compared with C1–O7–C4 in oxanorbornene (96.06°), bringing it closer to the ideal sp^3^ hybridization angle of ∼ 109.5° (Figure 2). Moreover, the changes in the H2–C2–C3–H3 and H2′–C2–C3–H3′ dihedral angles upon ring opening are considerably smaller in oxabicyclo[3.2.1]­oct-6-ene than in oxanorbornene, indicating its lower torsional strain.

Functionalization at C3 further modulates the torsional strain of the monomers, giving rise to **M1** and **M3**, with tunable RSEs ranging from 9.6 to 11.9 kcal/mol (Figure 2c,d). Similarly, **M2** and **M4** exhibit smaller angle strains than norbornene, corresponding to RSEs of 10.7 and 10.2 kcal/mol, respectively (Figure S80). Overall, although smaller than norbornene (16.7 kcal/mol) and oxanorbornene (18.7 kcal/mol), the RSEs of **M1**–**M4** (9.6–11.9 kcal/mol) remain significantly larger than those of low-strain cyclic olefins such as *trans*-cyclobutane-fused cyclooctene (4.9–5.3 kcal/mol)^31^ and cyclopentene (5.9 kcal/mol).^48^ The moderate-to-high RSEs of these (oxa)­bicyclo[3.2.1] monomers predict their excellent ROMP reactivity (vide infra).

### Investigation of Polymerization Thermodynamics

To shed light on the reversibility of bicyclo[3.2.1]-derived polymers, we determined their ceiling temperatures by examining the thermodynamic parameters of polymerization (Figures 3 and S10–S13, Tables S1–S4). ^1^H NMR spectroscopy was employed to acquire the equilibrium monomer concentration ([M]_eq_) during solution polymerization at different temperatures (Figures S10–S13). Using the van ’t Hoff analysis, Δ*H*_p_ and Δ*S*_p_° at the standard state (1 M) were obtained from the slope and intercept of the plot (Figure 3d–g), respectively.^28^ Consequently, the ceiling temperatures at 1 M were calculated from the ratio of Δ*H*_p_ to Δ*S*_p_°.

As shown in Figure 3, **M1**–**M4** exhibit Δ*H*_p_ values of −6.01 to −10.90 kcal/mol and Δ*S*_p_° values of −15.44 to −23.24 cal/mol/K, both markedly larger than those of previously reported depolymerizable ROMP systems (Figure S1). These thermodynamic parameters correspond to relatively low ceiling temperatures for **P1**–**P4**, ranging from 116 to 196 °C, which are considerably lower than that of polynorbornene (968 °C). Moreover, the entropic penalties of polymerization observed in these bicyclo[3.2.1] monomers are larger than that of norbornene, confirming the pivotal role of polymer rigidity in increasing the overall entropy change of polymerization by limiting the gain in conformational entropy (Figure S1a). It is worth noting that the observed enthalpy changes are smaller than their computed RSE values. This disparity between RSE and Δ*H*_p_ is common in ROMP and can be attributed to the formation of cyclic oligomers,^31^ and the energy absorbed by polymer conformations.^42^

Despite their relatively low ceiling temperatures, the polymerizability of bicyclo[3.2.1] monomers was not compromised, in contrast to low-strain monomers. Indeed, the calculated Gibbs free energy change of polymerization (Δ*G*_p_) for the bicyclo[3.2.1] monomers at 293.15 K was more negative than in previously reported low-strain depolymerizable systems and even *cis*-cyclooctene (a nondepolymerizable system), indicating their enhanced polymerizability (Figure S1a).

### Polymer Synthesis and Characterization

To evaluate the polymerizability of these bridged bicyclic monomers, ROMP was performed using the third-generation Grubbs catalyst (**G3**) in DCM (Figures 3c and S14–S28, Tables S5–S11). Polymerization kinetics studies reveal rapid reaction rates, with equilibrium established within 1 h (Tables S5, S7, and S9). Critically, high monomer conversions exceeding 87% were obtained under dilute conditions (<0.3 M) in all cases (Tables S6, S8, S10, and S11). Quantitative monomer conversion was achieved for **M2**–**M4**, confirming their excellent polymerizability arising from the substantial enthalpic driving force that results in a more negative ΔG_p_. This result is in good agreement with the Gibbs free energy changes of polymerization for these monomers (Figure S1).

Size exclusion chromatography (SEC) analyses indicate that the molecular weights of the resulting polymers are readily tunable by adjusting the monomer-to-initiator ratio (Figures 4a–c, S17 and S28). Moreover, the moderate-to-high RSEs are sufficient to enable the block copolymer synthesis via sequential monomer addition (Supplementary Section 4.9). As a proof of concept, **P3**-*b*-**P1** was obtained by chain extension of a **P3** macroinitiator with **M1**, as confirmed by SEC analysis and diffusion-ordered NMR spectroscopy (Figure S29). Notably, **P3** exhibits a narrower molecular weight distribution (*D̵* = 1.04–1.16) than **P1** (*D̵* = 1.65–1.80), **P2** (*D̵* = 1.83–1.95), and **P4** (*D̵* = 1.13–1.36) (Tables S6, S8, S10, and S11), indicating improved control over the polymerization of **M3**.

**Figure 4:**
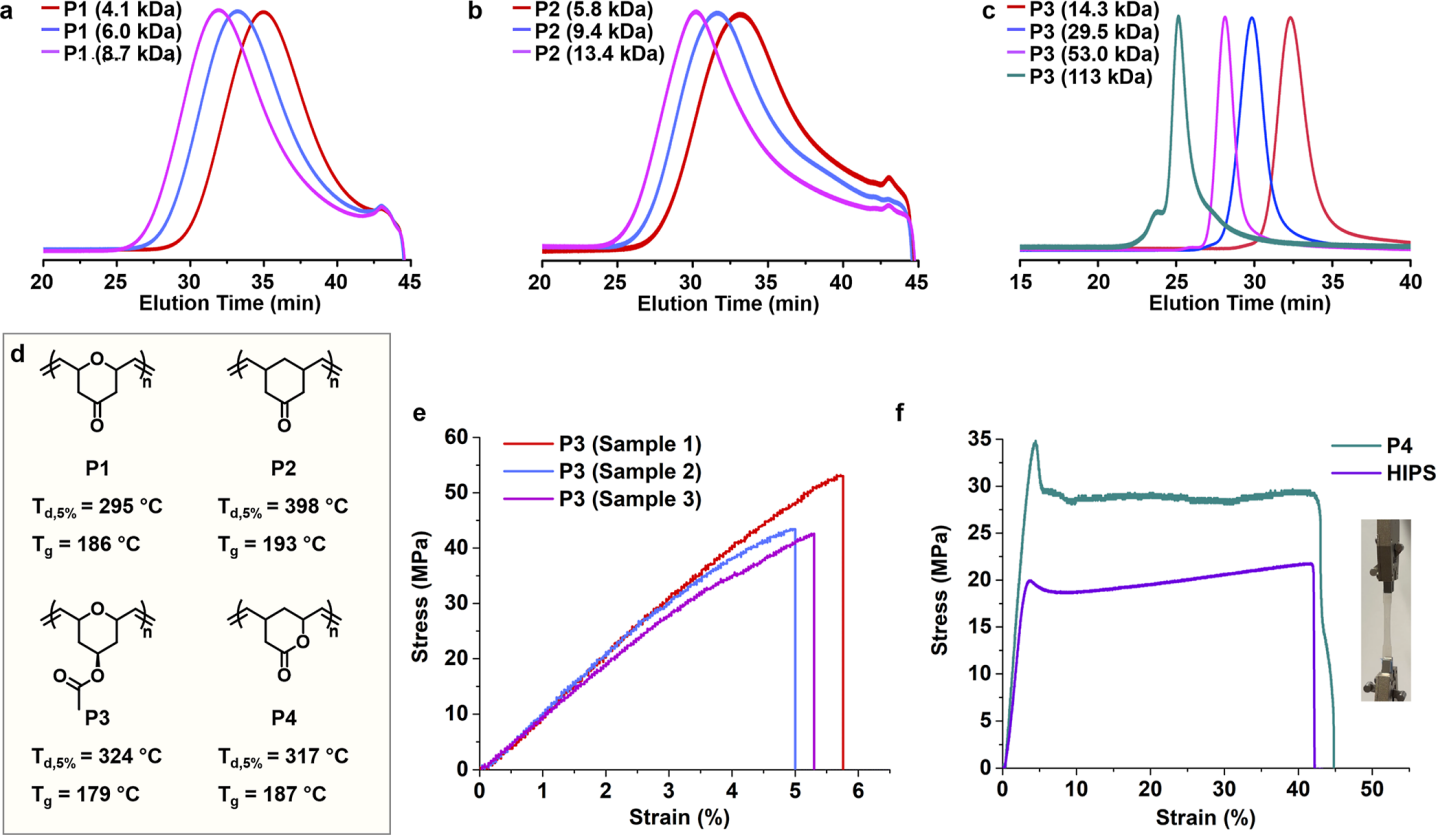
Synthesis of bicyclo[3.2.1]-derived polymers and their thermomechanical properties. (a–c) Size-exclusion chromatography traces of **P1** (a), **P2** (b), and **P3** (c). (d) Thermal properties of **P1**–**P4**. (e) Stress–strain curves of **P3** (*M*_n_ = 113 kDa, *D̵* = 1.16) obtained from tensile testing (5 mm/min, room temperature). (f) Representative stress–strain curves of **P4** (*M*_n_ = 47.5 kDa, *D̵* = 1.21) and commercial high-impact polystyrene (HIPS, *M*_n_ = 89 kDa, *D̵* = 2.0) measured under identical conditions (5 mm/min, room temperature). An inset shows a photograph of **P4** during tensile deformation.

The thermal properties of **P1**–**P4** were further investigated by thermogravimetric analysis (TGA) and differential scanning calorimetry (DSC). TGA revealed their excellent thermal stability, with decomposition temperatures exceeding 295 °C (Figures 4d and S58–S61). DSC demonstrated the high rigidity of these polymers, as reflected by glass transition temperatures (*T*_g_) of 179–193 °C (Figures 4d and S62–S65). The *T*_g_ values of these bicyclo[3.2.1]-derived polymers are noticeably higher than those of polynorbornenes, suggesting that six-membered backbone rings confer greater rigidity than five-membered rings.^49^

The high glass transition temperatures of the polymers developed in this study are expected to enhance their mechanical strength, yielding heat-resistant and mechanically robust materials suitable for applications in electronics, automotive components, and structural plastics.^50^ To evaluate their industrial promise, the mechanical properties of representative polymers **P3** and **P4** were assessed by tensile testing (Figures 4e,f and S66 and S67). Like typical high-*T*_g_ polymers, **P3** films are strong and stiff, exhibiting a Young’s modulus of 0.95 ± 0.07 GPa, an ultimate tensile strength of 46 ± 5.8 MPa, and a maximum strain of 5.3 ± 0.39% (Figure 4e). Impressively, **P4** shows a Young’s modulus of 0.93 ± 0.03 GPa, comparable to that of commercial high-impact polystyrene (HIPS, 0.92 ± 0.18 GPa), which accounts for 42% of global electronic-equipment waste (Figures 4f and S66 and S67).^51^ Both the ultimate tensile strength (32 ± 1.7 MPa) and maximum strain (50 ± 3.6%) of **P4** outperform those of HIPS (22 ± 0.9 MPa and 42 ± 4.2%, respectively), highlighting the potential of **P4** as a stronger, tougher, and more sustainable alternative to HIPS.

#### Depolymerization Studies

Given the relatively low ceiling temperatures of bicyclo[3.2.1]-based polymers, we reasoned that they should be capable of depolymerization via ring-closing metathesis.^52^ To verify this, **P1**–**P4** were subjected to depolymerization in the presence of the second-generation Grubbs catalyst (**G2**) (Figures 5 and S30–S55 and Tables S12–S14). Various reaction parameters, including polymer concentration, catalyst loading, and temperature, were screened to optimize the depolymerization efficiency (Tables S12–S14). Since the *T*_c_ (1 M) values of **P1**–**P4** remain far above the practical operating temperature of the Grubbs catalyst, we further calculated the *T*_c_ values under dilute conditions (0.02 M). Under these conditions, the ceiling temperatures decrease to −14 °C for **P1**, 46 °C for **P2**, 78 °C for **P3**, and 42 °C for **P4** (Figure S1c). Further dilution to 0.005 M reduces the *T*_c_ value of **P3** to 50 °C. Therefore, dilute conditions and a temperature of 55 °C were employed to drive the depolymerization. Under the optimized conditions (55 °C and 5 mol % G2), all polymers achieved over 74% monomer regeneration, as confirmed by NMR and SEC analyses (Figure 5). Notably, **P1** depolymerized with quantitative recovery of **M1** even at 200 mM, consistent with its low ceiling temperature (Figure S41). In contrast, bicyclo[2.2.1]-based polynorbornenone showed no monomer recovery under the same conditions (Figure S56).

**Figure 5:**
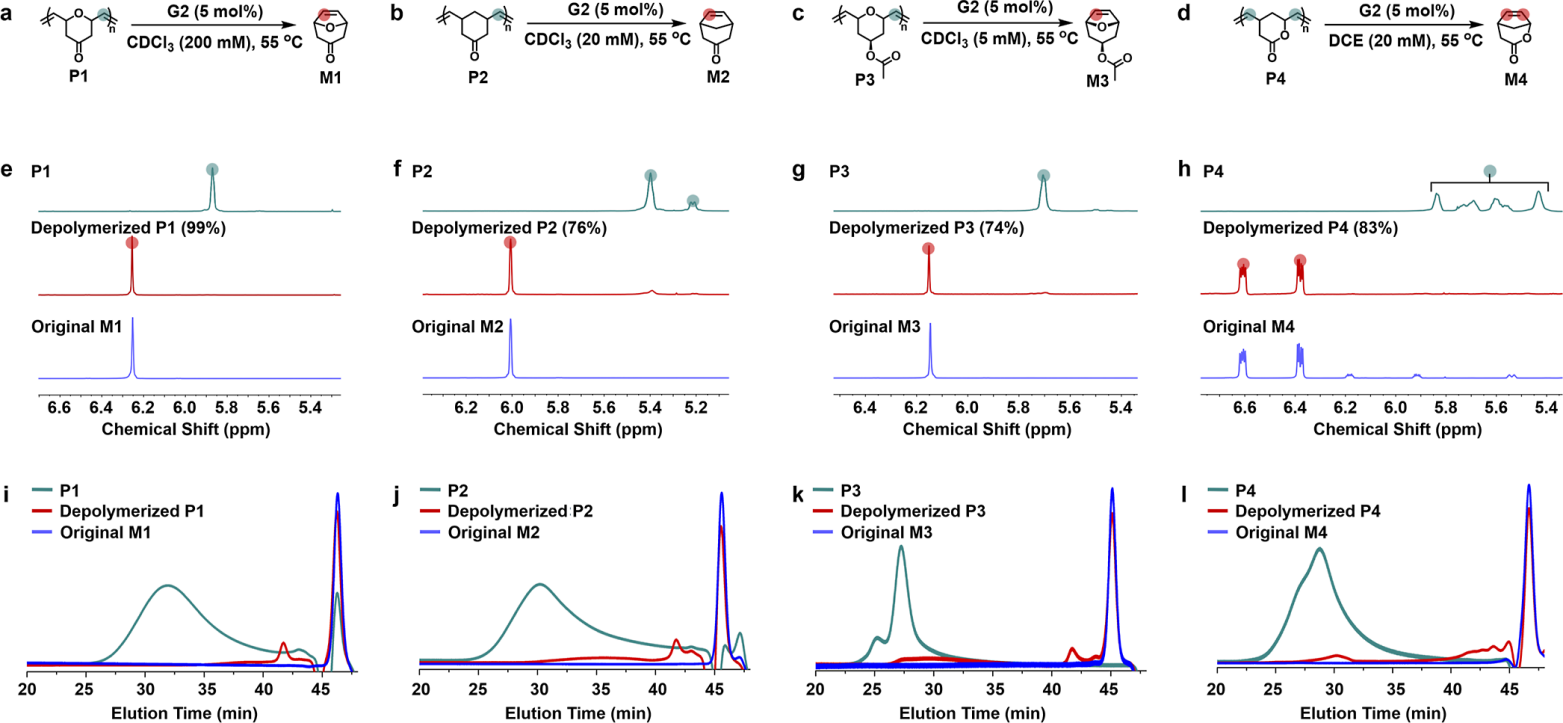
Depolymerization studies of bicyclo[3.2.1]-based polymers. (a–d) Schematic illustrations of the depolymerization of polymers **P1** (a), **P2** (b), **P3** (c), and **P4** (d). (e–h) Partial ^1^H NMR spectra of **P1** (e), **P2** (f), **P3** (g), and **P4** (h) before (green) and after (red) 12 h of heating in CDCl_3_ or 1,2-dichloroethane (DCE) at 55 °C in the presence of second-generation Grubbs catalyst (**G2**). The ^1^H NMR spectra of the corresponding monomers (blue) are shown for reference. (i–l) Size exclusion chromatography (SEC) traces for **P1** (i), **P2** (j), **P3** (k), and **P4** (l) before (in green) and after (in red) the depolymerization. The SEC traces of corresponding monomers (blue) are shown for comparison.

Sequential and selective depolymerization is desired for the chemical recycling of mixed plastic wastes^53–55^ and for the design of nanostructured materials.^56^ The distinct ceiling temperatures of **P1** (116 °C) and **P3** (196 °C) are expected to enable sequential depolymerization of the block copolymer **P3**-*b*-**P1**, allowing efficient recycling of both components even when covalently linked. To achieve this, **P3**-*b*-**P1** was first treated at a high polymer concentration (100 mM of olefin groups) to favor depolymerization of the **P1** block. NMR analysis indicated >60% recovery of **M1** with minimal depolymerization of **P3** (<7%) (Figure S57), and SEC confirmed the presence of **M1** signal with negligible **M3** (Figure 6). Subsequent dilution to 10 mM and increased catalyst loading promoted depolymerization of the more stable **P3** block, yielding 79% recovery of **M3**, demonstrating sequential depolymerization of the block copolymer (Figure S57).

**Figure 6:**
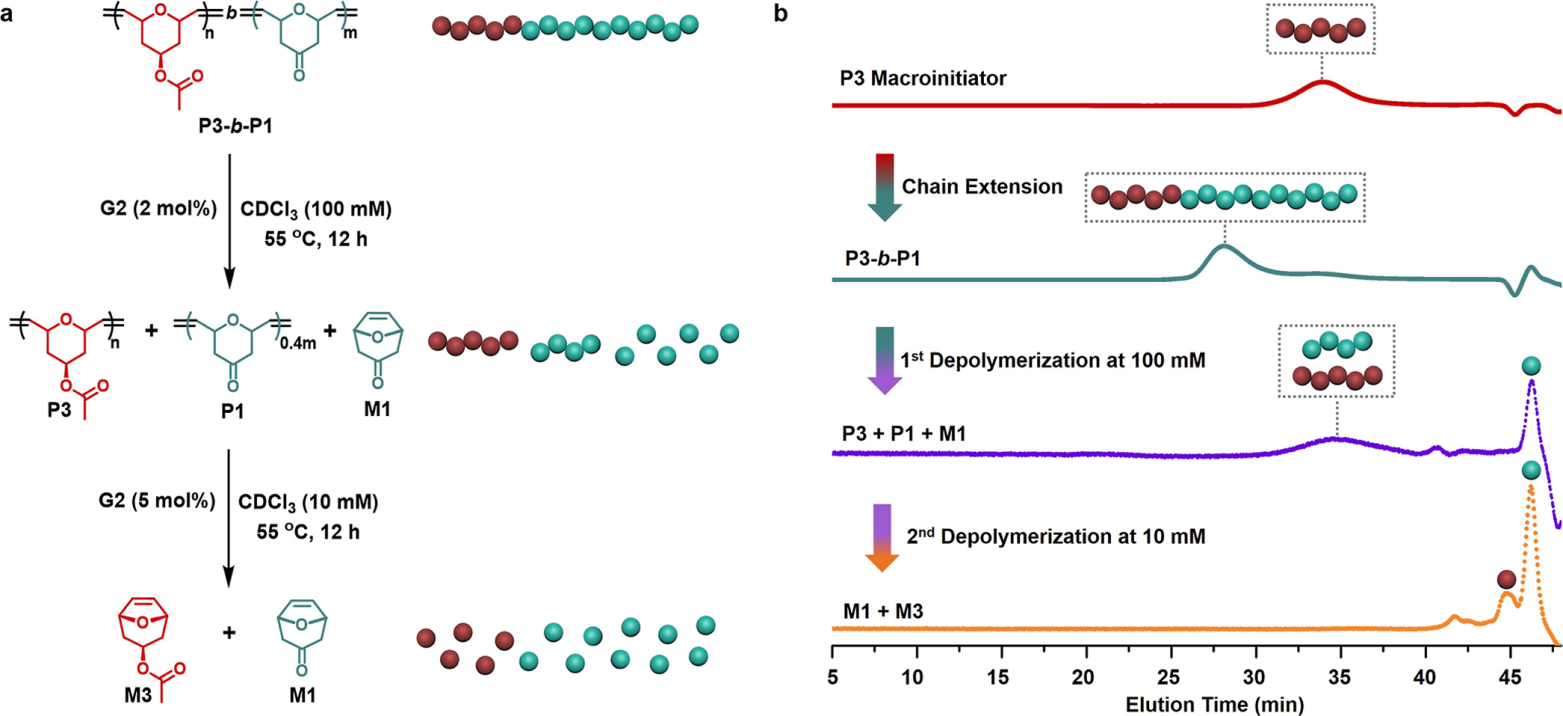
Sequential depolymerization of block copolymer. (a) Schematic illustration of the sequential depolymerization of **P3**-*b*-**P1** in two steps. The first depolymerization was carried out with 2 mol % of **G2** in CDCl_3_ at 55 °C with 100 mM of olefin groups. The second depolymerization was performed with 5 mol % of **G2** in CDCl_3_ at 55 °C with 10 mM of olefin groups. (b) Size-exclusion chromatography traces of the **P3** macroinitiator (red), **P3**-*b*-**P1** (green), the depolymerized product after the first depolymerization (purple), and the products after the second depolymerization (orange).

## Conclusions

Leveraging the design of bicyclo[3.2.1] monomers with moderate-to-high ring strain energies, we present a low-*T*_c_, depolymerizable polymer system characterized by both a large enthalpy change and a large entropy change of polymerization. The substantial ring strain energies of these monomers not only provide sufficient driving force for efficient polymerization, but also enable the synthesis of depolymerizable block copolymers, thereby broadening the architectural diversity of depolymerizable polymers. Moreover, the resulting bicyclo[3.2.1]-derived polymers were capable of ring-closing metathesis depolymerization, a feature not observed in traditional strained bridged bicyclic monomers such as norbornene derivatives.

We demonstrate that polymer backbone rigidity critically influences the entropic penalty of polymerization: the limited number of freely rotatable bonds restricts conformational entropy gain, thereby increasing the overall entropic penalty. This high backbone rigidity leads to materials with elevated glass transition temperatures and enhanced mechanical properties compared to commercial high-impact polystyrene. Given the highly modular structure of modern synthetic polymers, we envision that this strategy can be extended beyond ring-opening metathesis polymerization, providing a general approach to a new generation of sustainable polymers exhibiting both exceptional polymerizability and depolymerizability.

## Experimental Methods

### Polymer Synthesis

In a typical procedure for the synthesis of P1 (entry 2 in Table S2) at room temperature, M1 (248 mg, 2.00 mmol, 100 equiv) was dissolved in DCM (4.6 mL) and degassed with argon. Separately, a solution of G3 catalyst (18 mg, 0.020 mmol, 1.0 equiv) in degassed DCM (2.0 mL) was prepared and quickly added to the monomer solution. The reaction mixture was stirred for 12 h. Polymerization was quenched with excess ethyl vinyl ether (190 μL, 2.00 mmol, 100 equiv). The resulting solution was precipitated into cold methanol to afford the polymer.

### Investigation of Polymerization Thermodynamics

To investigate the thermodynamics of polymerization, the reactions were carried out in DCM at various temperatures ranging from 0 to 30 °C using 1 mol % of G3 catalyst. The initial monomer concentration ([M]_0_) varied depending on the monomer. Each polymerization was allowed to proceed for 12 h to reach equilibrium. The equilibrium monomer concentrations ([M]_eq_) were determined by ^1^H NMR and are summarized in Tables S1–S4. By plotting the logarithm of [M]_eq_ against the inverse of temperature, the thermodynamic parameters, including the change in enthalpy of polymerization (Δ*H*_p_) and the standard-state change in entropy (Δ*S*_p_^*o*^), were obtained from the slope and intercept of the van ’t Hoff plot based on eq 1.^37^

1 $${\ln\left[M\right]}_{eq}=\frac{\Delta H_{p}}{R}\left(\frac{1}{T}\right)-\frac{\Delta S_{p}^{{}^{\circ}}}{R}$$

By using these thermodynamic parameters, the ceiling temperature at 1 M can be calculated based on eq 2.

2 $$T_{c}\left(1M\right)=\frac{\Delta H_{p}}{\Delta S_{p}^{{}^{\circ}}}$$

### Depolymerization Study

In a typical procedure for the ring-closing metathesis depolymerization of polymers at a concentration of 20 mM in olefin groups, the polymer (0.04 mmol of backbone olefin groups, 20 equiv) and G2 (1.7 mg, 0.002 mmol, 1.0 equiv) were dissolved in CDCl_3_ (2.0 mL) and degassed with argon. The reaction mixture was stirred at 55 °C for 12 h. Depolymerization was quenched with excess ethyl vinyl ether (20 μL, 0.20 mmol, 100 equiv). After quenching for 20 min, the solution was analyzed by NMR to determine the depolymerization yields.

## Supplementary Material



## Notes

Supplementary figures and tables, characterization data, ^1^H and ^13^C NMR spectra of monomers and polymers, SEC analysis of polymers, TGA and DSC analyses, tensile testing results, computational studies, and detailed experimental protocols (PDF)
