# Supplementary material for: Entropy-Driven Design of Depolymerizable Polyolefins from Strained Bridged Bicyclic Monomers
Source: J Am Chem Soc. 2026 Mar 25;148(15):16317–25. doi: 10.1021/jacs.6c02456 (PMC13107448; doi:10.1021/jacs.6c02456)
Supplement: Supplementary file 1 [file ja6c02456_si_001.pdf]

Supplementary Information for

Entropy-Driven Design of Depolymerizable Polyolefins from Strained Bridged Bicyclic Monomers

Tarek Ibrahim, Desmond Brown, and Hao Sun*

Department of Chemistry and Chemical & Biomedical Engineering, Tagliatela College of Engineering, University of New Haven, West Haven, Connecticut 06516, USA

Corresponding to: Dr. Hao Sun, Email: hasun@newhaven.edu

Supplementary Information:

Materials and Instrumentation

Computational Study

Experimental Methods

Scheme S1

Figures S1 to S80

Tables S1 to S15

Optimized Geometries of Monomers and Their Ring-Opened Structures from DFT Calculations

References

Table of Contents

1. Materials 3
2. Instrumentation 3
3. Computational Study 4
4. Experimental Methods 5
   4.1. Synthesis of 8-Oxabicyclo[3.2.1]oct-6-en-3-one (M1) 5
   4.2. Synthesis of Bicyclo[3.2.1]oct-6-en-3-one (M2) 6
   4.3. Synthesis of M3 7
   4.4. Synthesis of M4 8
   4.5. ROMP Synthesis of P1 9
   4.6. ROMP Synthesis of P2 9
   4.7. ROMP Synthesis of P3 10
   4.8. ROMP Synthesis of P4 10
   4.9. Synthesis of Block Copolymer P3-*b*-P1 11
   4.10. Investigation of Polymerization Thermodynamics 11
   4.11. Depolymerization Study 12
5. Supplementary Scheme, Figures, and Tables 13

References 106

1. Materials

1,1,3,3-tetrabromoacetone (>98%), furan (>99%), dicyclopentadiene (> 97%), zinc–copper couple (Cu/Zn), acetic anhydride (>99%), 1.2-dibromoethane (98%), iodine (99.5%), anhydrous acetonitrile (>99.8%), methanol (99%), ammonium chloride (>99.5%), ethanol (99.5%), ethyl vinyl ether (99%), 1,2-dichloroethane (> 99%), 1,2-diiodoethane (> 97%), 2-propanol (IPA) (99.5%), Celite 545, and triethylamine (TEA) (> 99.5%) were purchased from Fisher Scientific and used without purification. Samarium (Sm, powder), zinc (99.995%), 4-(dimethylamino)pyridine (DMAP) (>99%), 3-chloroperbenzoic acid (mCPBA, < 77%), $CDCl_3$ (99.8 atom % D), CD_2Cl_2 (99.5 atom % D), DMSO-d_6 (99.9 atom % D), 1,2-dichloroethane-d_4 (99 atom % D), anhydrous dichloromethane (DCM, 99.8%), bicyclo[2.2.1]hept-5-en-2-one (norbornenone, 95%), Grubbs' catalyst 2nd generation (M204), and Grubbs' catalyst 3rd generation (M300) were purchased from Sigma Aldrich and used without purification. High-impact polystyrene (HIPS) was purchased from MEGA FORMAT. Cyclopentadiene was freshly obtained by retro-Diels-Alder reaction of dicyclopentadiene above 150 °C.[1]

2. Instrumentation

Nuclear Magnetic Resonance (NMR): ^{1}H NMR spectra were recorded on a Bruker spectrometer (400 MHz) in deuterated solvents. Chemical shifts are given in ppm downfield from tetramethylsilane (TMS).

Size Exclusion Chromatography (SEC): The molecular weight and polydispersity of synthetic polymers were determined by a size exclusion chromatography (SEC) system (TOSOH EcoSEC HLC-8320) equipped with a set of Phenomenex Phenogel 5μ, 1K-75K, 300 x 7.80 mm in series with a Phenomex Phenogel 5μ, 10K-1000K, 300 x 7.80 mm columns following a guard column and two detectors including a RI detector and a UV detector. The measurements were performed

using HPLC-grade THF, chloroform (0.75% ethanol), or DMF as the eluent at a flow rate of 0.5 mL/min at 35 °C and a series of polystyrene standards for the calibration of the columns.

Differential Scanning Calorimetry (DSC): Differential scanning calorimetry (DSC) measurements were performed using a Guangdong Newgoer DSC-300C system under a nitrogen gas flow (100 mL/min). Two thermal cycles with heating and cooling rates of 10 °C/min were performed. The glass transition temperatures were obtained from the second heating scans after removing the thermal history of polymers.

Thermogravimetric Analysis (TGA): Thermogravimetric analysis (TGA) was performed using a TA SDT Q600 system under a nitrogen gas flow (100 mL/min) with a heating rate of 10 °C/min. The temperature range for the analysis extended from ambient temperature to 650 °C.

Tensile Test: Tensile testing of polymers was performed using a Mark-10 F305-IMT system with a 200 N load at room temperature. A crosshead speed of 5 mm/min was used. P3 and P4 films were solvent-cast from a concentrated DCM solution in a covered Petri dish overnight, then dried under vacuum at 40 °C for two days. Rectangular samples (10 mm (L) × 5 mm (W)) were then cut from the polymer film using a razor blade.

3. Computational Study

The ring strain energies of cyclic olefin monomers were calculated using density functional theory (DFT) with Spartan software. To examine the various conformations available in both the monomers and their ring-opened products, an exhaustive conformer search was performed at the AM1 level of theory. The geometries and energies of the resulting conformers were then optimized at the B3LYP/6-31G(d) level of theory in the gas phase. The enthalpy changes (ΔH) were estimated as the enthalpy difference between the ring-opened products and the total enthalpy of the isolated reactants (monomer + ethylene), using their lowest-energy conformers.

4. Experimental Methods

4.1. Synthesis of 8-Oxabicyclo[3.2.1]oct-6-en-3-one (M1)

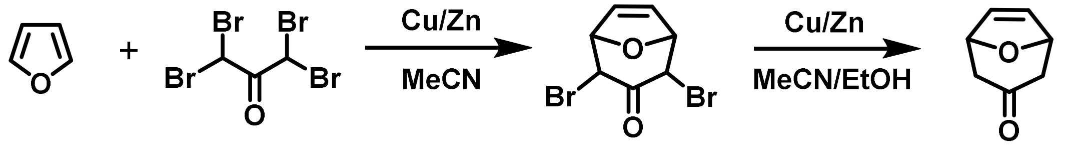

M1 was synthesized following a modified procedure adapted from prior work.[2]

Step 1: [4+3] Cycloaddition. In a typical protocol, furan (5.0 mL, 0.069 mmol), Cu/Zn couple (10.3 g, 0.16 mmol), and 1,2-dibromoethane (0.13 g, 6.9 μmol) were suspended in 40 mL of anhydrous acetonitrile and cooled to 5 °C under argon. The mixture was further sonicated for 30 min, after which a solution of 1,1,3,3-tetrabromoacetone (19.0 g, 0.051 mmol) in 20 mL of acetonitrile was added dropwise. The resulting mixture was sonicated at 10 °C, and the reaction progress was monitored by GC-MS. After confirming the completion of reaction, the mixture was filtered through a pad of Celite 545. The crude solution of 1,5-dibromo-8-oxabicyclo[3.2.1]oct-6-en-3-one (Compound 1) was used directly in the next step without purification.

Step 2: Debromination. Cu/Zn couple (23.5 g, 0.36 mmol) and ammonium chloride (13.0 g, 0.24 mol) were suspended in 50 mL of anhydrous ethanol and cooled to −78 °C under argon. The solution of 1,5-dibromo-8-oxabicyclo[3.2.1]oct-6-en-3-one was then added dropwise over 2 h. The reaction was warmed to room temperature, and the progress was monitored by GC–MS. Upon completion, the Cu/Zn couple was removed by filtration and washed with DCM. The filtrate was evaporated, and the residue was cooled in an ice bath and neutralized with saturated sodium bicarbonate solution. The resulting suspension was filtered and washed with DCM. The organic layer was dried over sodium sulfate and concentrated by vacuum evaporation at room temperature.

The crude product was further purified by flash chromatography (20% EtOAc/hexanes) to yield M1 as yellow crystals (2.98 g, 47% overall yield).

4.2. Synthesis of Bicyclo[3.2.1]oct-6-en-3-one (M2)

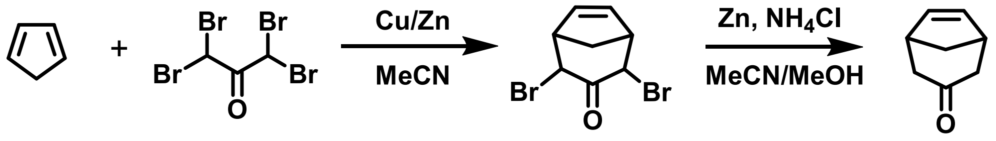

Compound 2 M2

M2 was synthesized following a modified procedure adapted from prior work.[2]

Step 1: [4+3] Cycloaddition. In a typical protocol, Cu/Zn couple (10.3 g, 0.16 mmol) and iodine (0.18 g, 6.9 µmol) were suspended in 40 mL of anhydrous acetonitrile and cooled to 5 °C under argon. The mixture was further sonicated for 30 min, after which a solution of cyclopentadiene (4.6 g, 0.069 mmol) and 1,1,3,3-tetrabromoacetone (19.0 g, 0.051 mmol) in 30 mL of acetonitrile was added dropwise. The resulting mixture was sonicated at 10 °C, and the reaction progress was monitored by GC-MS. After confirming the completion of reaction, the mixture was filtered through a pad of Celite 545. The crude solution of 1,5-dibromo-bicyclo[3.2.1]oct-6-en-3-one (Compound 2) was used directly in the next step without purification.

Step 2: Debromination. Activated Zn (10.0 g, 0.15 mmol) and ammonium chloride (10.0 g, 0.19 mol) were suspended in 50 mL of anhydrous methanol and cooled to 0 °C under argon. The solution of 1,5-dibromo-bicyclo[3.2.1]oct-6-en-3-one was then added dropwise with sonication over 2 h. The reaction was monitored by GC–MS. Upon completion, the Zinc residue was removed by filtration and washed with diethyl ether. The resulting suspension was cooled in an ice bath and neutralized with saturated sodium bicarbonate solution. The resulting suspension was filtered and washed with diethyl ether. The organic layer was dried over sodium sulfate and concentrated by

vacuum evaporation at room temperature. The crude product was further purified by flash chromatography (10% EtOAc/hexanes) to yield yellow crystals (2.52 g, 40% overall yield).

4.3. Synthesis of M3

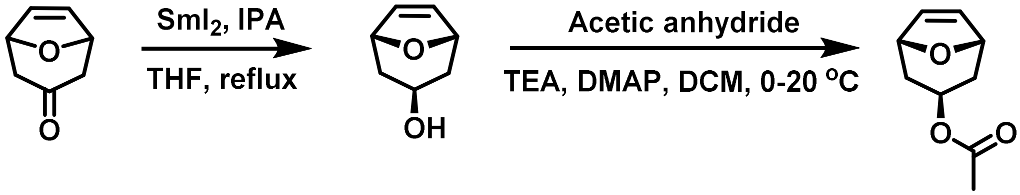

Step 1: Stereoselective reduction. The exo-hydroxy-functional product (Compound 3) was synthesized following a modified procedure based on prior work.[3] A 100-mL three-necked flask equipped with a gas inlet and reflux condenser was charged with Sm (2.48 g, 16.5 mmol) and diiodoethane (3.7 g, 13.2 mmol). The apparatus was flushed with nitrogen. The mixture was then cooled to 0 °C, and 35 mL of anhydrous THF was added slowly under a nitrogen atmosphere. The mixture was stirred for 10 min at 0 °C and 10 min at room temperature, then heated to reflux, during which the solution turned dark blue. M1 (1.0 g, 8.1 mmol) was mixed with 2-propanol (0.62 mL, 8.1 mmol) and dissolved in 17 mL of anhydrous THF. This solution was added slowly to the refluxing SmI_2 solution, and the reaction mixture was refluxed for 3 h.

Upon completion of the reaction and cooling to room temperature, the dark blue reaction mixture was treated with distilled water and 1 M HCl solution. The layers were separated, and the aqueous phase was extracted with ethyl acetate. The combined organic phases were treated sequentially with saturated aqueous $Na_2S_2O_3$ and saturated aqueous $NaHCO_3$ to remove iodine, then dried over Na_2SO_4. The solvent was removed under reduced pressure, and the resulting crude product was carried forward to the next step without further purification.

Step 2: Acetylation. In a dry flask, compound 3 (0.65 g, 5.1 mmol), triethylamine (1.1 mL, 7.7 mmol), and DMAP (94 mg, 0.77 mmol) were suspended in 17 mL of anhydrous DCM. A solution of acetic anhydride (0.73 mL, 7.7 mmol) in 8 mL of dry DCM was added dropwise to the reaction mixture at 0 °C. The mixture was stirred at 0 °C for 3 h, then allowed to warm to room temperature and stirred for an additional 12 h. The reaction solution was washed sequentially with saturated sodium bicarbonate, 1 M HCl, and brine. The organic phase was dried over Na_2SO_4 and concentrated under reduced pressure using a rotary evaporator. The crude product was purified by flash chromatography (30% EtOAc/hexanes) to afford M3 as yellow solid (0.47 g, 55% overall yield).

4.4. Synthesis of M4

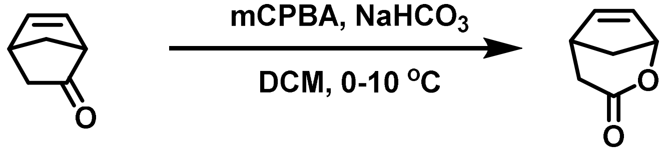

Bicyclo[2.2.1]hept-5-en-2-one **M4**

In a typical protocol, bicyclo[2.2.1]hept-5-en-2-one (2.16 g, 20.0 mmol) and $NaHCO_3$ (2.10 g, 25.0 mmol) were suspended in anhydrous DCM (50 mL). mCPBA (5.60 g, 25.0 mmol) was added portionwise to the reaction mixture over 3 h. The mixture was stirred at 0–10 °C for 10 h. The DCM solution was washed sequentially with saturated $NaHCO_3$ and saturated aqueous $Na_2S_2O_3$, then dried over Na_2SO_4. The solvent was removed under vacuum to yield M4 as a colorless liquid (1.93 g, 78%). The purity of M4 was estimated by ^{1}H NMR and GC-MS. Due to the instability of M4 in the slightly acidic environment of silica gel, column chromatography was not feasible for further purification. Nevertheless, since the impurity is inert to the Grubbs catalyst, crude M4 was used directly for ROMP.

4.5. ROMP Synthesis of P1

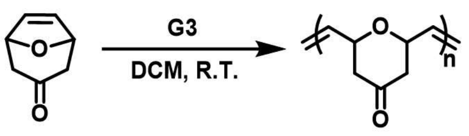

M1 **P1**

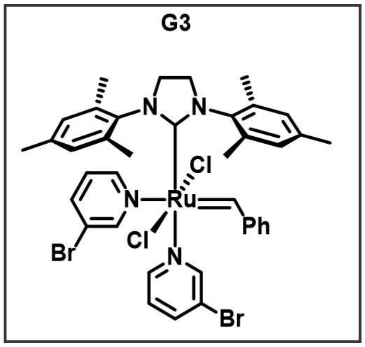

In a typical procedure for the synthesis of P1 (entry 2 in Table S2) at room temperature, M1 (248 mg, 2.00 mmol, 100 equiv.) was dissolved in DCM (4.6 mL) and degassed with argon. Separately, a solution of G3 catalyst (18 mg, 0.020 mmol, 1.0 equiv.) in degassed DCM (2.0 mL) was prepared and quickly added to the monomer solution. The reaction mixture was stirred for 12 h. Polymerization was quenched with excess ethyl vinyl ether (190 μL, 2.00 mmol, 100 equiv.). The resulting solution was precipitated into cold methanol to afford the polymer.

4.6. ROMP Synthesis of P2

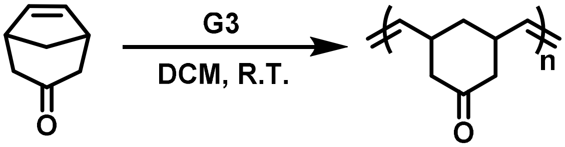

M2 **P2**

In a typical procedure for the synthesis of P2 (entry 4 in Table S4) at room temperature, M2 (244 mg, 2.00 mmol, 100 equiv.) was dissolved in DCM (18 mL) and degassed with argon. Separately, a solution of G3 catalyst (18 mg, 0.020 mmol, 1.0 equiv.) in degassed DCM (2.0 mL) was prepared and quickly added to the monomer solution. The reaction mixture was stirred for 12 h. Polymerization was quenched with excess ethyl vinyl ether (190 μL, 2.00 mmol, 100 equiv.). The resulting solution was concentrated and then precipitated into cold methanol to afford the polymer.

4.7. ROMP Synthesis of P3

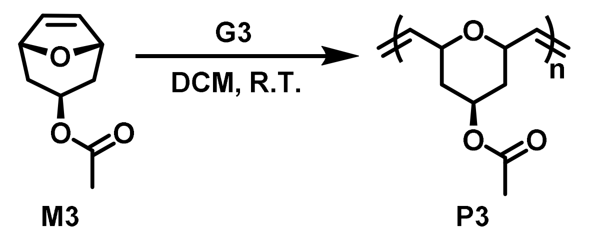

In a typical procedure for the synthesis of P3 (entry 3 in Table S6) at room temperature, M3 (202 mg, 1.20 mmol, 120 equiv.) was dissolved in DCM (23 mL) and degassed with argon. Separately, a solution of G3 catalyst (9.0 mg, 0.010 mmol, 1.0 equiv.) in degassed DCM (1.0 mL) was prepared and quickly added to the monomer solution. The reaction mixture was stirred for 12 h. Polymerization was quenched with excess ethyl vinyl ether (95 μL, 1.00 mmol, 100 equiv.). The resulting solution was concentrated and then precipitated into cold methanol to afford the polymer.

4.8. ROMP Synthesis of P4

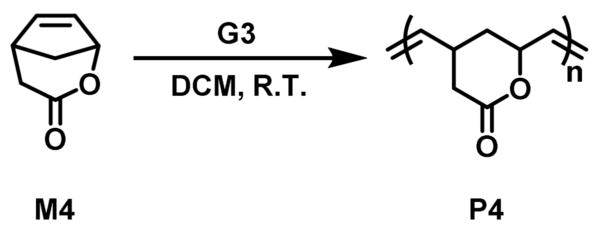

In a typical procedure for the synthesis of P4 (entry 2 in Table S8) at room temperature, M4 (372 mg, 3.00 mmol, 150 equiv.) was dissolved in DCM (18 mL) and degassed with argon. Separately, a solution of G3 catalyst (18 mg, 0.020 mmol, 1.0 equiv.) in degassed DCM (2.0 mL) was prepared and quickly added to the monomer solution. The reaction mixture was stirred for 12 h. Polymerization was quenched with excess ethyl vinyl ether (190 μL, 2.00 mmol, 100 equiv.). The resulting solution was concentrated and then precipitated into cold methanol to afford the polymer.

4.9. Synthesis of Block Copolymer P3-*b*-P1

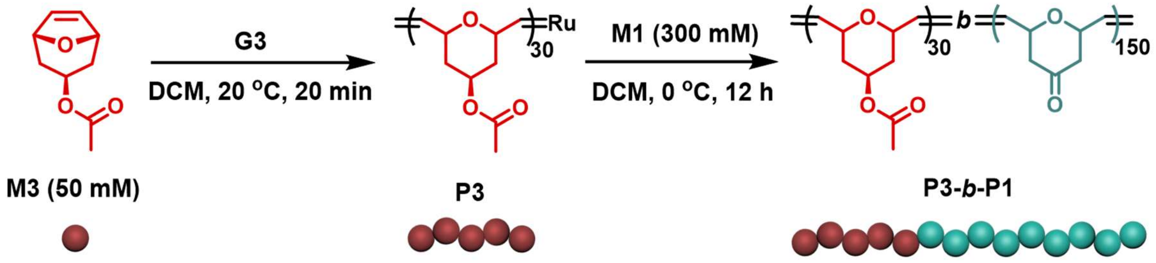

Step 1: Synthesis of Macroinitiator. M3 (30 mg, 0.18 mmol, 30 equiv.) was dissolved in degassed DCM (2.6 mL). Separately, G3 (5.3 mg, 0.006 mmol, 1 equiv.) was dissolved in degassed DCM (1.0 mL). The M3 solution was quickly added to the G3 solution and stirred for 20 min. A small aliquot was removed for NMR analysis of monomer conversion (>97%) and for GPC analysis of the P3 macroinitiator.

Step 2: Chain Extension. M1 (134 mg, 1.08 mmol, 180 equiv.) was then added to the macroinitiator solution under an argon atmosphere. The reaction mixture was immediately placed in an ice–water bath at 0 °C for 12 h until the conversion of M1 reached equilibrium (84%). After polymerization, the resulting block copolymer was recovered by precipitation into cold methanol.

4.10. Investigation of Polymerization Thermodynamics

To investigate the thermodynamics of polymerization, the reactions were carried out in DCM at various temperatures ranging from 0 °C to 30 °C using 1 mol% of G3 catalyst. The initial monomer concentration ($[M]_0$) varied depending on the monomer. Each polymerization was allowed to proceed for 12 hours to reach equilibrium. The equilibrium monomer concentrations ($[M]_{eq}$) were determined by ^{1}H NMR and are summarized in Tables S9–S12. By plotting the logarithm of $[M]_{eq}$ against the inverse of temperature, the thermodynamic parameters, including the change in enthalpy of polymerization ($\boldsymbol{\Delta H_p}$) and the standard-state change in entropy ($\boldsymbol{\Delta S_p^o}$), were obtained from the slope and intercept of the van't Hoff plot based on Equation 1.[4]

$$\ln[\mathrm{M}]_{\mathrm{eq}} = \frac{\Delta H_p}{\mathrm{R}}\left(\frac{1}{\mathrm{T}}\right) - \frac{\Delta S_p^o}{\mathrm{R}} \qquad \text{Equation 1}$$

By using these thermodynamic parameters, the ceiling temperature at 1M can be calculated as T_c (1M) = $\Delta H_p / \Delta S_p^o$.

4.11. Depolymerization Study

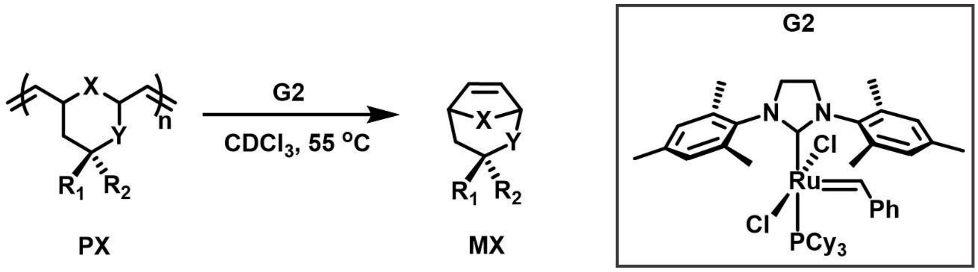

In a typical procedure for the ring-closing metathesis depolymerization of polymers at a concentration of 20 mM in olefin groups, the polymer (0.04 mmol of backbone olefin groups, 20 equiv.) and G2 (1.7 mg, 0.002 mmol, 1.0 equiv.) were dissolved in $CDCl_3$ (2.0 mL) and degassed with argon. The reaction mixture was stirred at 55 °C for 12 h. Depolymerization was quenched with excess ethyl vinyl ether (20 µL, 0.20 mmol, 100 equiv.). After quenching for 20 min, the solution was analyzed by NMR to determine the depolymerization yields.

5. Supplementary Scheme, Figures, and Tables

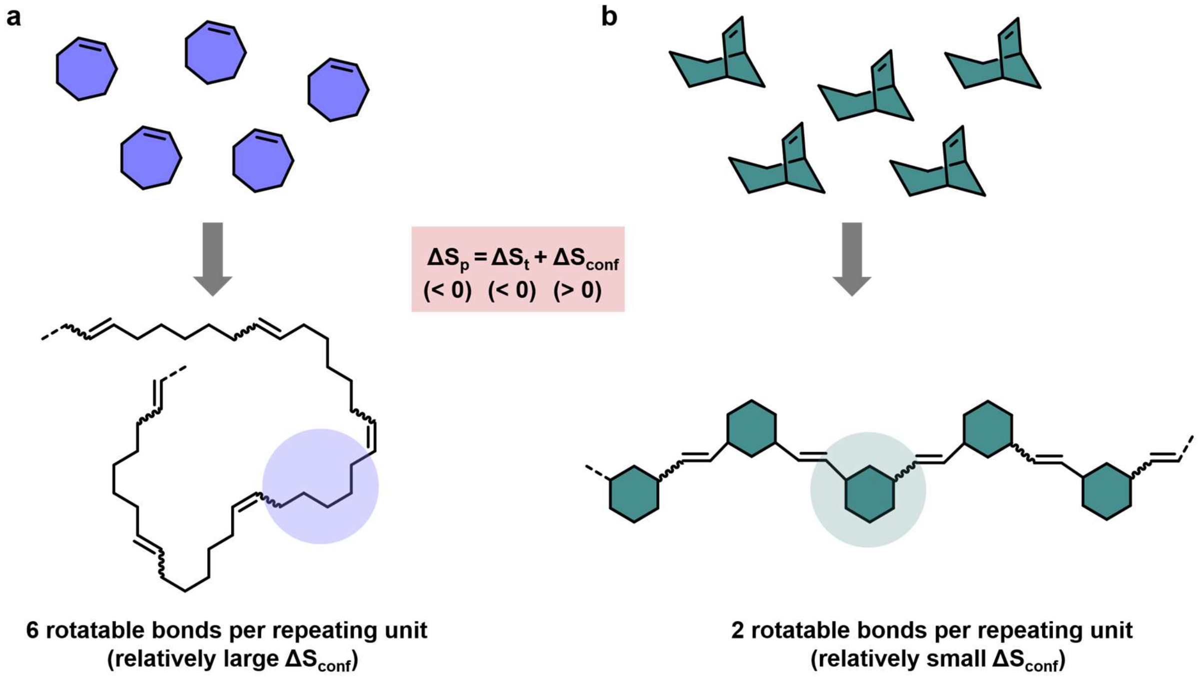

Scheme S1. **Entropic penalty of polymerization**. Schematic illustration of the entropy changes associated with the polymerization of a representative monocyclic monomer, cycloheptene (**a**), and a representative bridged bicyclic monomer, bicyclo[3.2.1]oct-6-ene (**b**). At the same concentration, the translational entropy change (ΔS_t) is expected to be similar for both monomers due to their comparable ring sizes. However, the polymer derived from the bridged bicyclic monomer possesses a more rigid backbone with fewer freely rotatable bonds, resulting in a smaller conformational entropy gain (ΔS_{conf}). Therefore, the overall entropic penalty (ΔS_p) for the ROMP of the bridged bicyclic monomer is expected to be larger (i.e., more negative).

a

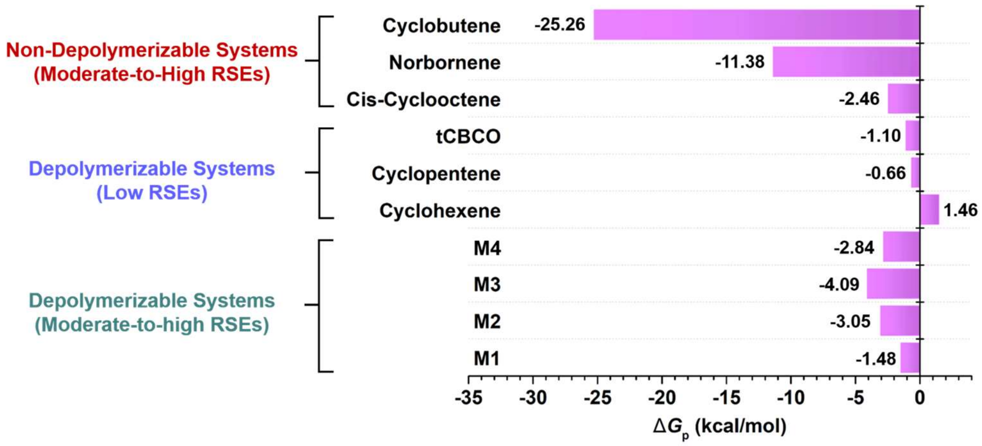

b

Monomers	ΔH_p (kcal/mol)	ΔS_p^o (cal/mol/K)	ΔG_p (293.15 K) (kcal/mol)	T_c (1 M) (°C)
**Cyclobutene**[5]	-28.9	-12.4	-25.26	2057
**Norbornene**[5]	-14.9	-12.0	-11.38	968
***Cis*-Cyclooctene**[5]	-3.1	-2.2	-2.46	1136
**tCBCO**[6]	-2.1	-3.4	-1.10	335
**Cyclopentene**[5]	-4.3	-12.4	-0.66	74
**Cyclohexene**[5]	-0.5	-6.7	1.46	-199
**M1**	-6.01	-15.44	-1.48	116
**M2**	-9.52	-22.07	-3.05	158
**M3**	-10.90	-23.24	-4.09	196
**M4**	-8.11	-17.98	-2.84	178

c

Monomers	ΔH_p (kcal/mol)	ΔS_p (0.02 M) (cal/mol/K)	ΔG_p (323.15 K) (kcal/mol)	T_c (0.02 M) (°C)
**M1**	-6.01	-23.21	1.49	-14
**M2**	-9.52	-29.84	0.12	46
**M3**	-10.90	-31.01	-0.88	78
**M4**	-8.11	-25.75	0.21	42

Figure S1. Polymerization thermodynamics of cyclic olefin monomers. (**a**) The Gibbs free energy changes (ΔG_p) of polymerization for cyclic olefin monomers at 293.15 K. ΔG_p values were calculated from the of ΔH_p and ΔS_p^o values summarized in **b**, using the equation: $\Delta G_p = \Delta H_p - T\Delta S_p^o$. tCBCO stands for *trans*-cyclobutane-fused cyclooctene, as reported in ref. 6. (**b)** Summary of polymerization thermodynamic parameters of cyclic olefin monomers at 1 M. Data for cyclobutene, norbornene, *cis*-cyclooctene, cyclopentene, and cyclohexene were obtained from a previous report,[5] and data for tCBCO was obtained from a previous work.[6] (**c**) Summary of polymerization thermodynamic parameters of M1–M4 at 0.02 M. ΔS_p^o (0.02 M) was calculated accoding to the equation: $\Delta S_p([M]) = \Delta S_p^o - R\ln(\frac{[M]_o}{[M]})$, as reported in a previous report.[7]

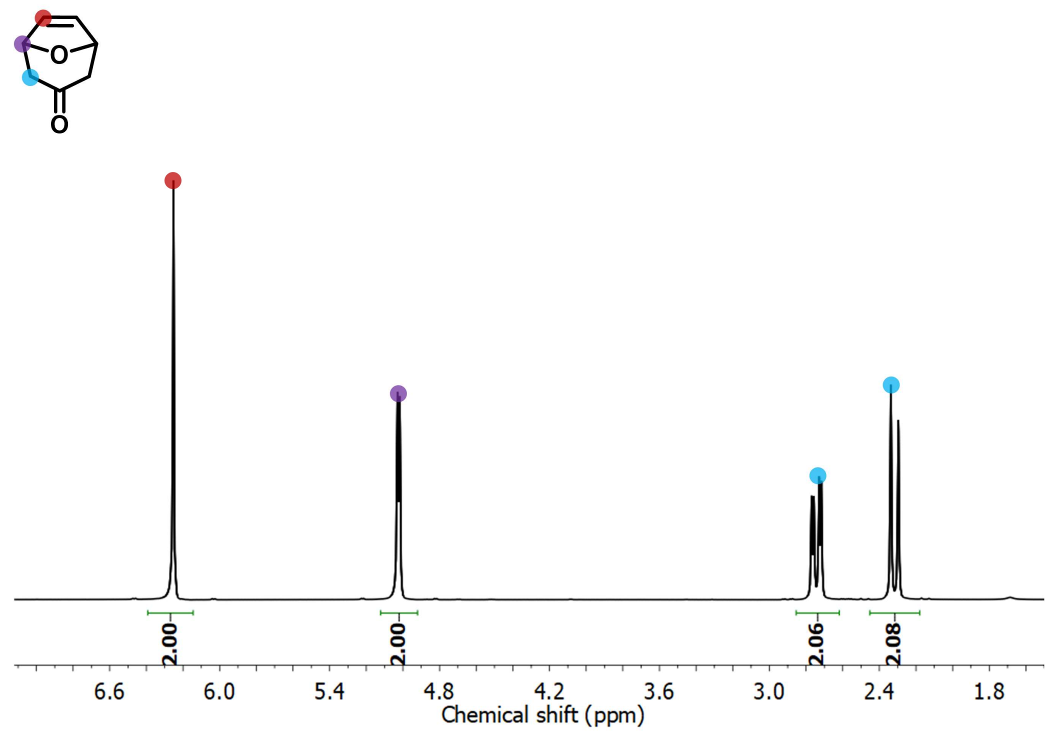

Figure S2. ^{1}H NMR spectrum of M1 in $CDCl_3$ at room temperature.

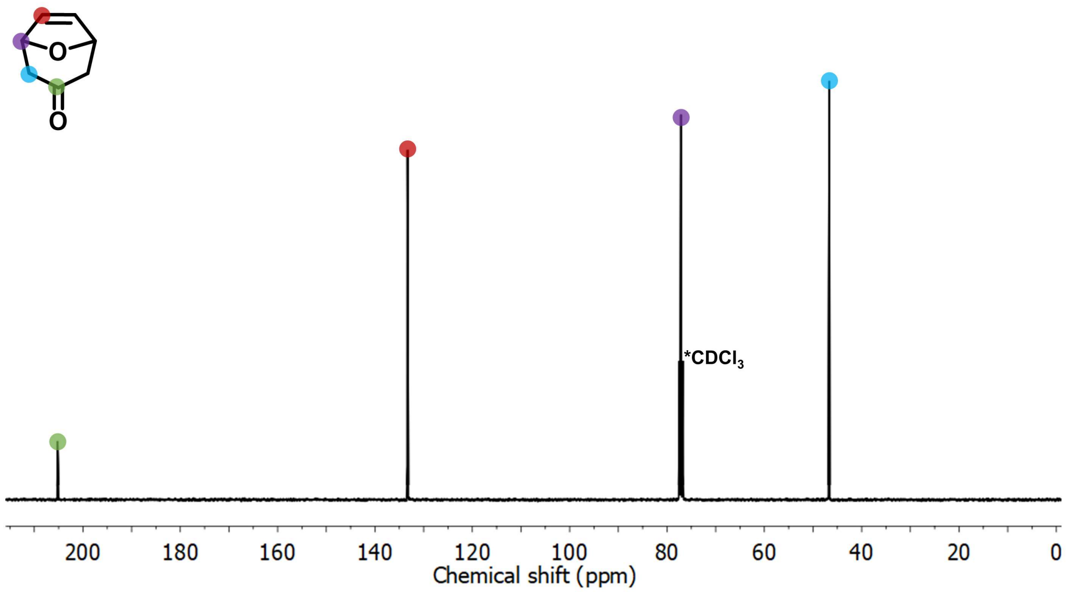

Figure S3. ^{13}C NMR spectrum of M1 in $CDCl_3$ at room temperature.

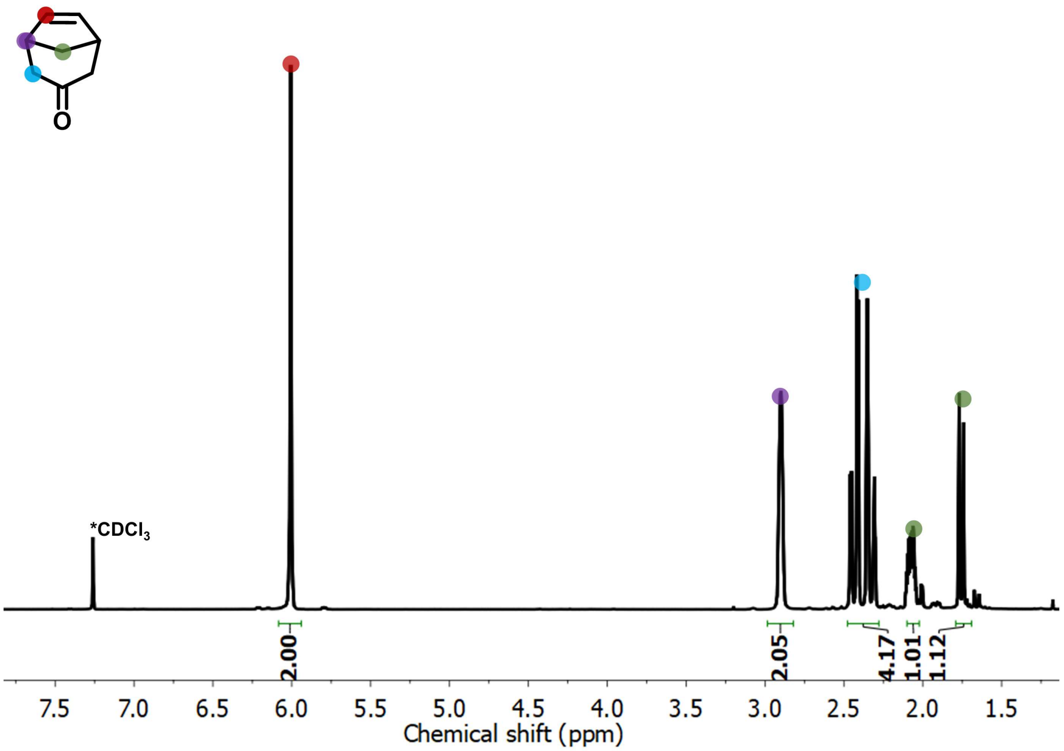

Figure S4. ^{1}H NMR spectrum of M2 in $CDCl_3$ at room temperature.

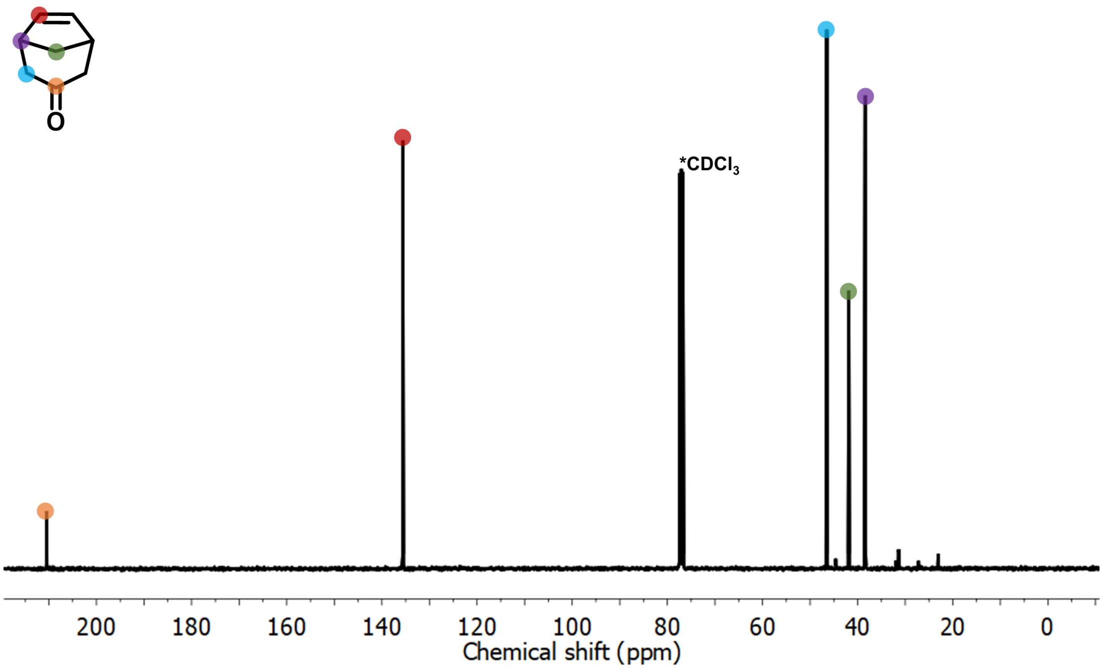

Figure S5. ^{13}C NMR spectrum of M2 in $CDCl_3$ at room temperature.

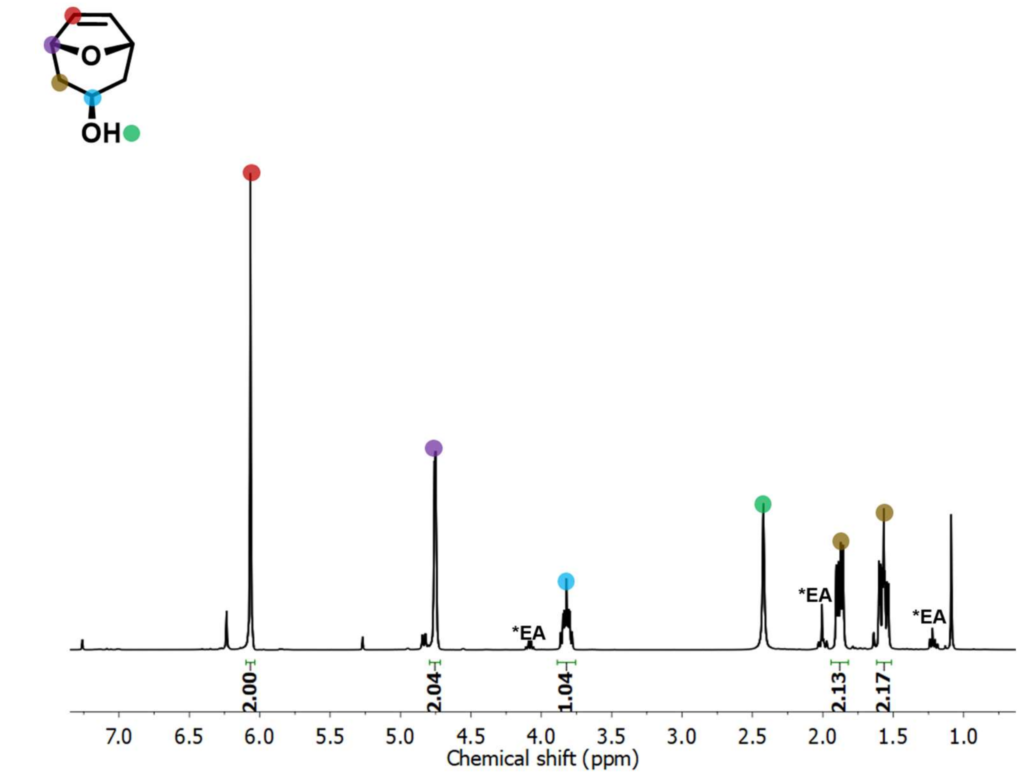

Figure S6. ^{1}H NMR spectrum of compound 3 in $CDCl_3$ at room temperature.

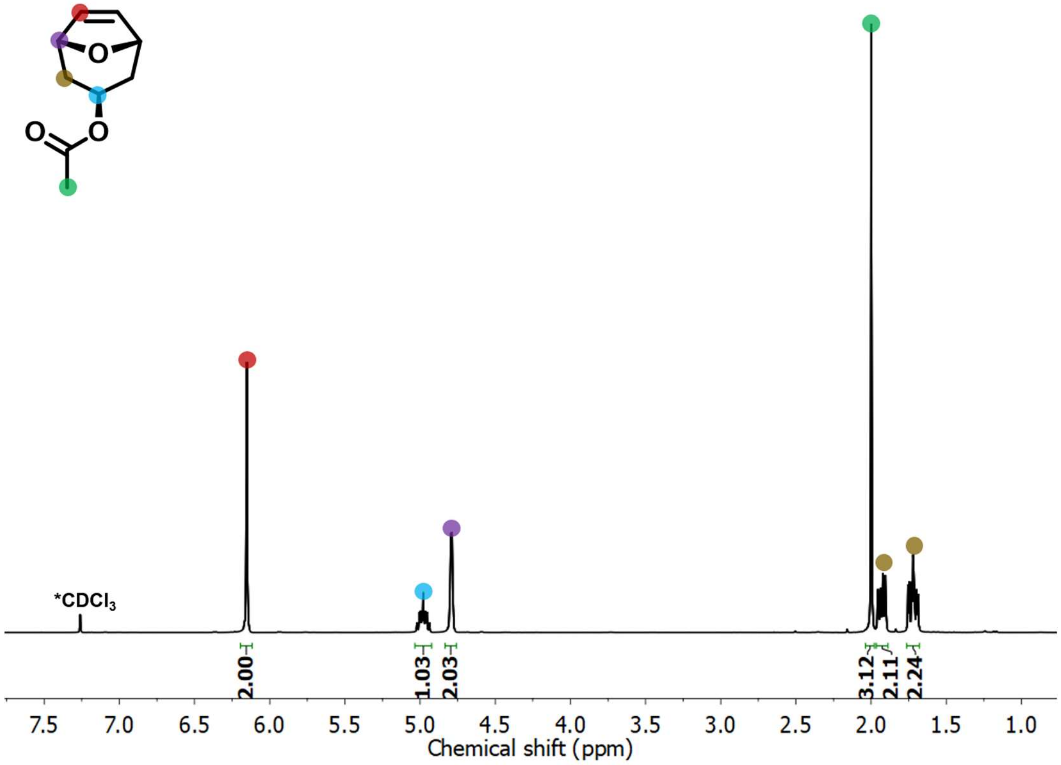

Figure S7. ^{1}H NMR spectrum of M3 in $CDCl_3$ at room temperature.

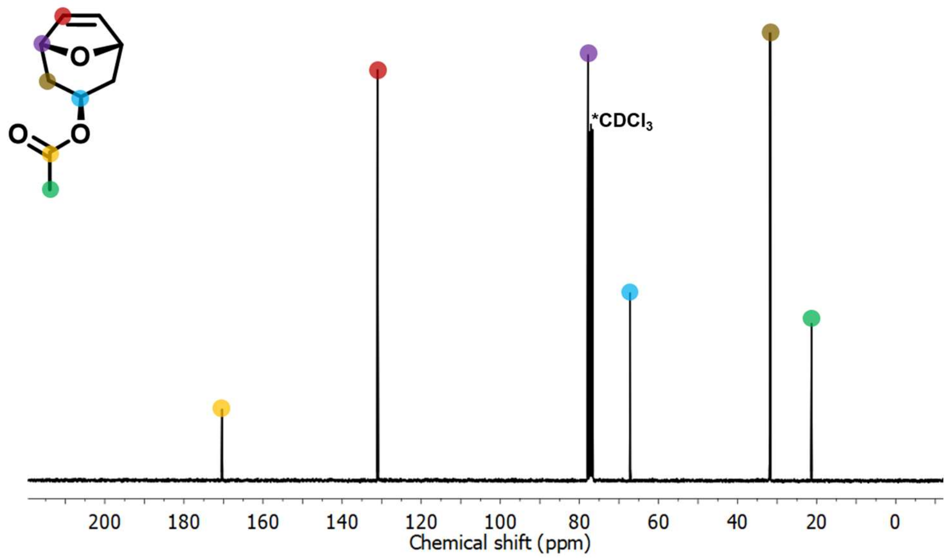

Figure S8. ^{13}C NMR spectrum of M3 in $CDCl_3$ at room temperature.

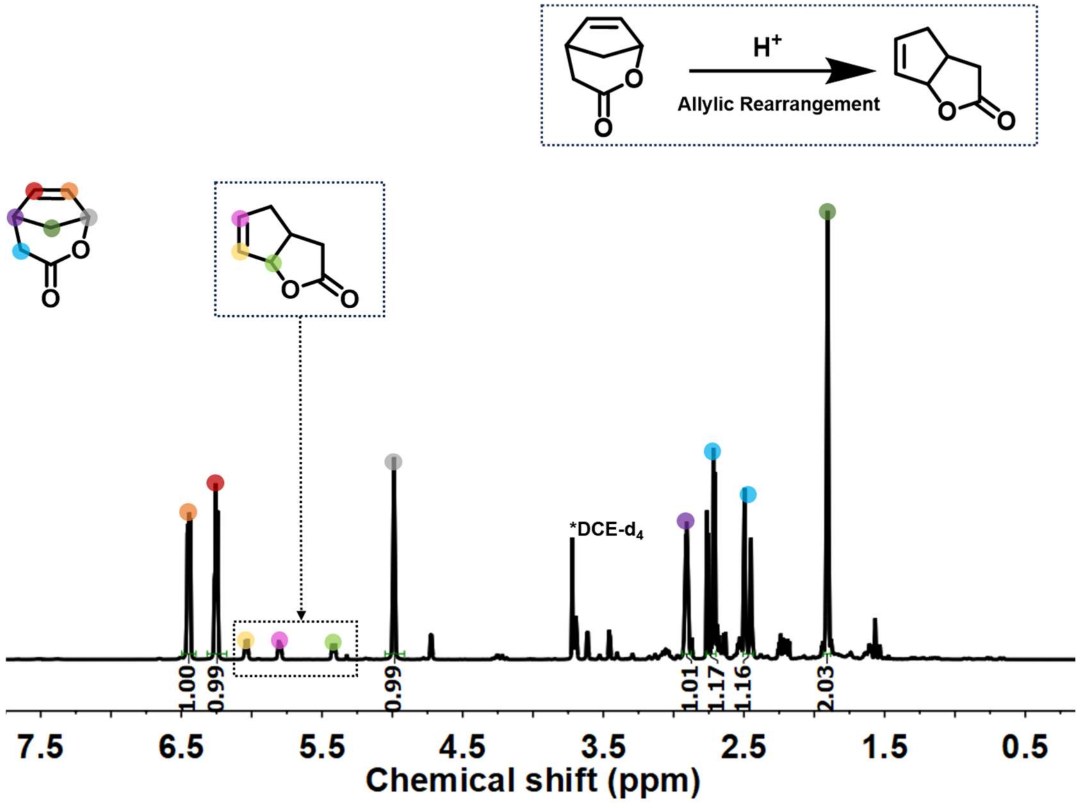

Figure S9. ^{1}H NMR spectrum of M4 in DCE-d_4 at room temperature. M4 is unstable under acidic conditions and undergoes allylic rearrangement to form a fused-ring cyclopentene side product. Nevertheless, because this impurity is inert to the Grubbs catalyst, the crude M4 was used directly for ROMP.

Table S1. ROMP of M1 at various temperatures (initial concentration = 300 mM)

Entry	M1/G3	Temperature (K)	1/T (K^{-1})	Time (h)	Conv. (%)	$[M]_{eq}$ (M)	$ln[M]_{eq}$
1	100	273.15	0.00366099	12	87.9	0.0363	-3.31594
2	100	283.15	0.00353170	12	81.8	0.0545	-2.90873
3	100	293.15	0.00341122	12	73.0	0.0810	-2.51331
4	100	303.15	0.0032987	12	64.5	0.107	-2.23961

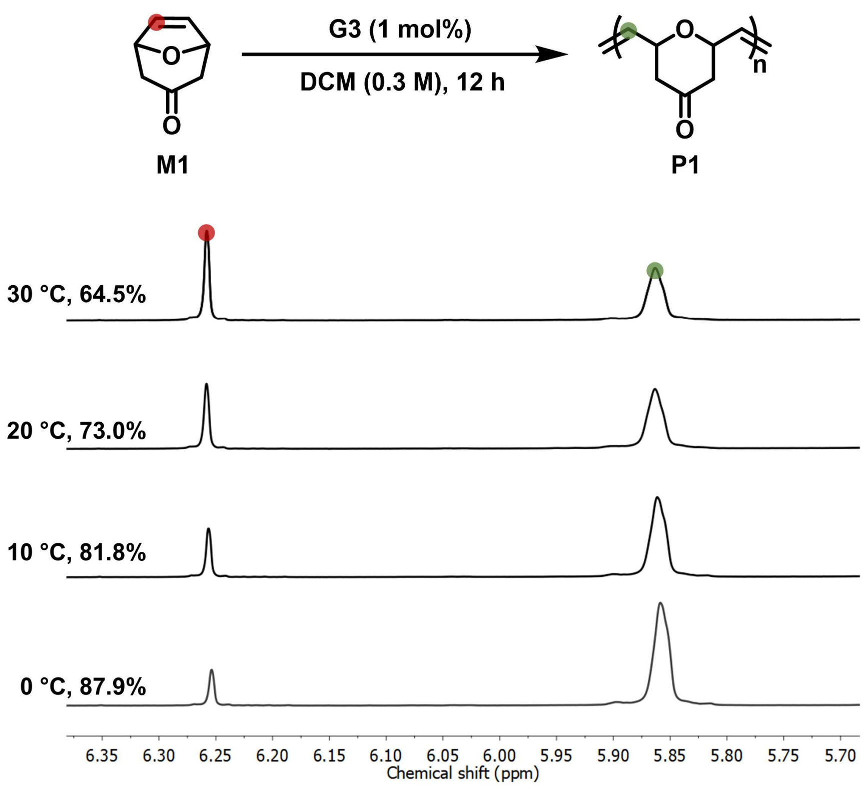

Figure S10. Partial ^{1}H NMR spectra for the thermodynamic study of the polymerization of M1.

Table S2. ROMP of M2 at various temperatures (initial concentration = 25 mM)

Entry	M2/G3	Temperature (K)	1/T (K^{-1})	Time (h)	Conv. (%)	$[M]_{eq}$ (M)	$\ln[M]_{eq}$
1	100	273.15	0.00366099	12	93.6	0.0016	-6.43775
2	100	283.15	0.00353170	12	88.1	0.0030	-5.80914
3	100	293.15	0.00341122	12	79.0	0.0053	-5.24005
4	100	303.15	0.0032987	12	63.8	0.0091	-4.69948

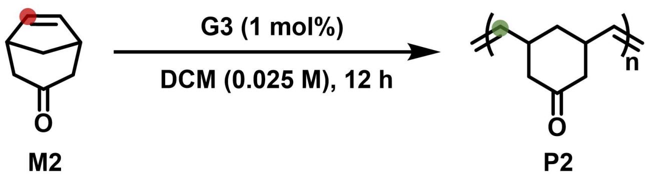

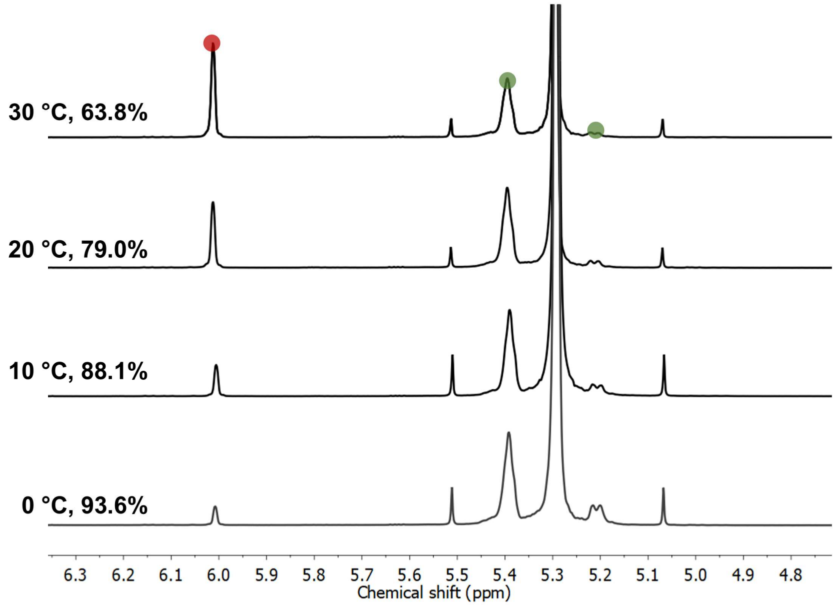

Figure S11. Partial ^{1}H NMR spectra for the thermodynamic study of the polymerization of M2.

Table S3. ROMP of M3 at various temperatures (initial concentration = 10 mM)

Entry	M3/G3	Temperature (K)	1/T (K^{-1})	Time (h)	Conv. (%)	$[M]_{eq}$ (M)	$\ln[M]_{eq}$
1	100	273.15	0.00366099	12	97.7	0.00023	-8.37743
2	100	283.15	0.00353170	12	95.4	0.00046	-7.68428
3	100	294.15	0.00339963	12	90.1	0.00099	-6.91781
4	100	305.15	0.00327708	12	81.4	0.00186	-6.28718

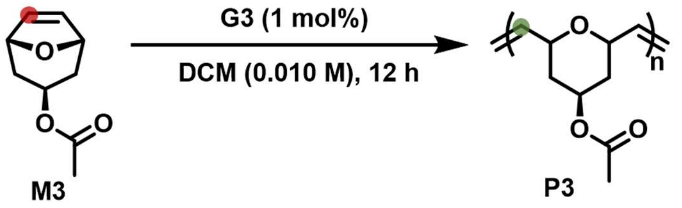

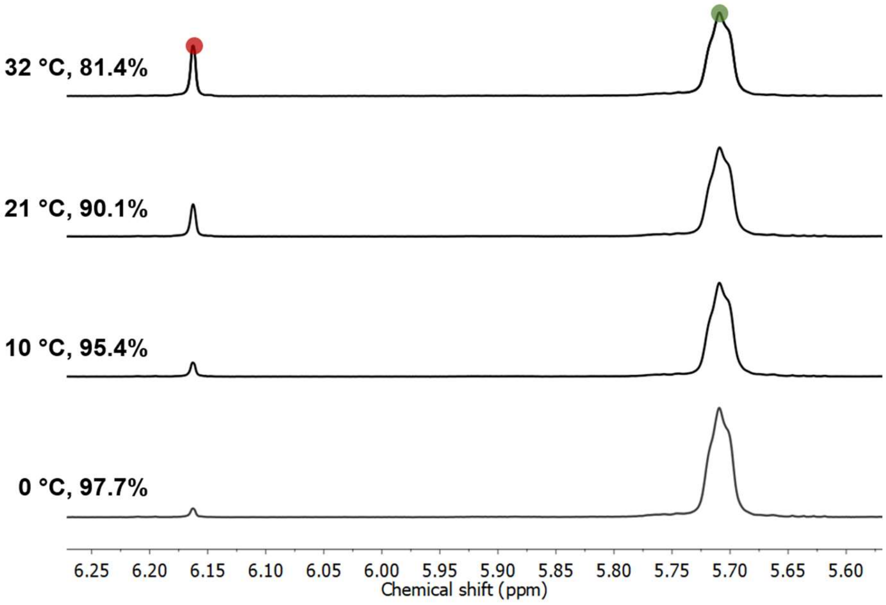

Figure S12. Partial ^{1}H NMR spectra for the thermodynamic study of the polymerization of M3.

Table S4. ROMP of M4 at various temperatures (initial concentration = 25 mM)

Entry	M4/G3	Temperature (K)	1/T (K^{-1})	Time (h)	Conv. (%)	$[M]_{eq}$ (M)	$ln[M]_{eq}$
1	100	273.15	0.00366099	12	88.1	0.00298	-5.9158320
2	100	283.15	0.00353170	12	80.8	0.00480	-5.3391394
3	100	293.15	0.00341122	12	69.3	0.00768	-4.8691357
4	100	303.15	0.0032987	12	52.4	0.0119	-4.4312169

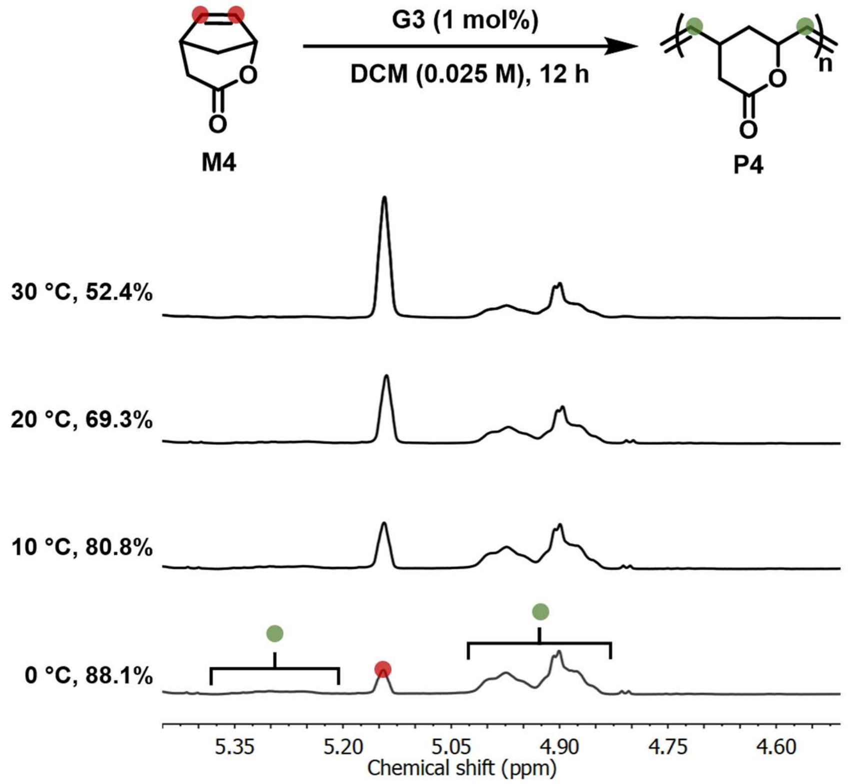

Figure S13. Partial ^{1}H NMR spectra for the thermodynamic study of the polymerization of M4.

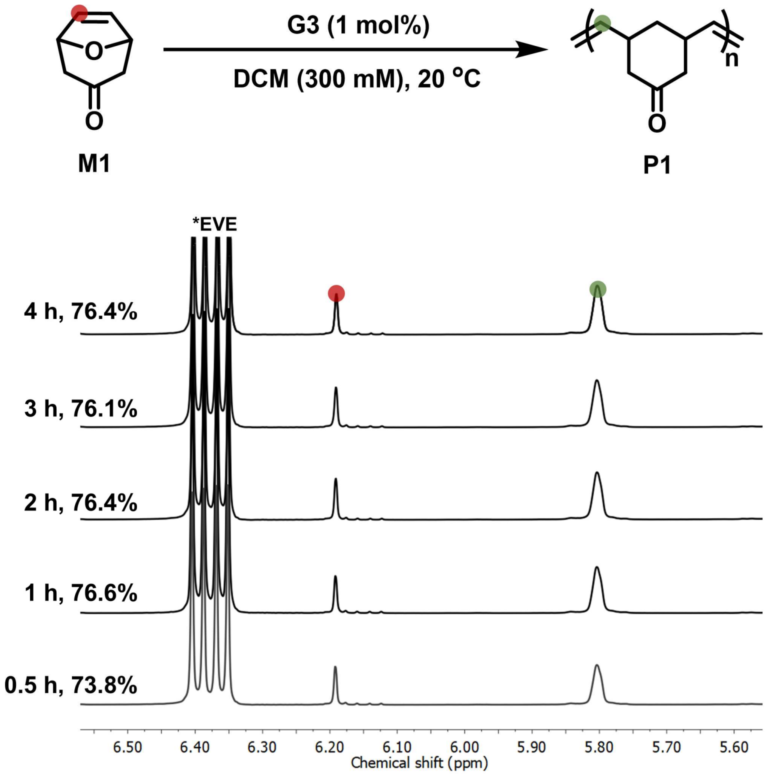

Figure S14. Partial ^{1}H NMR spectra for the polymerization kinetics of M1(300 mM) in DCM at 20 °C. The polymerization reached equilibrium within 1 h.

Table S5. Polymerization kinetics of M1 in DCM at 20 °C

Entry	M1/G3	$[M]_0$ (mM)	Temperature (°C)	Time (h)	Conv. (%)
1	100	300	20	0.5	73.8
2	100	300	20	1	76.6
3	100	300	20	2	76.4
4	100	300	20	3	76.1
5	100	300	20	4	76.4

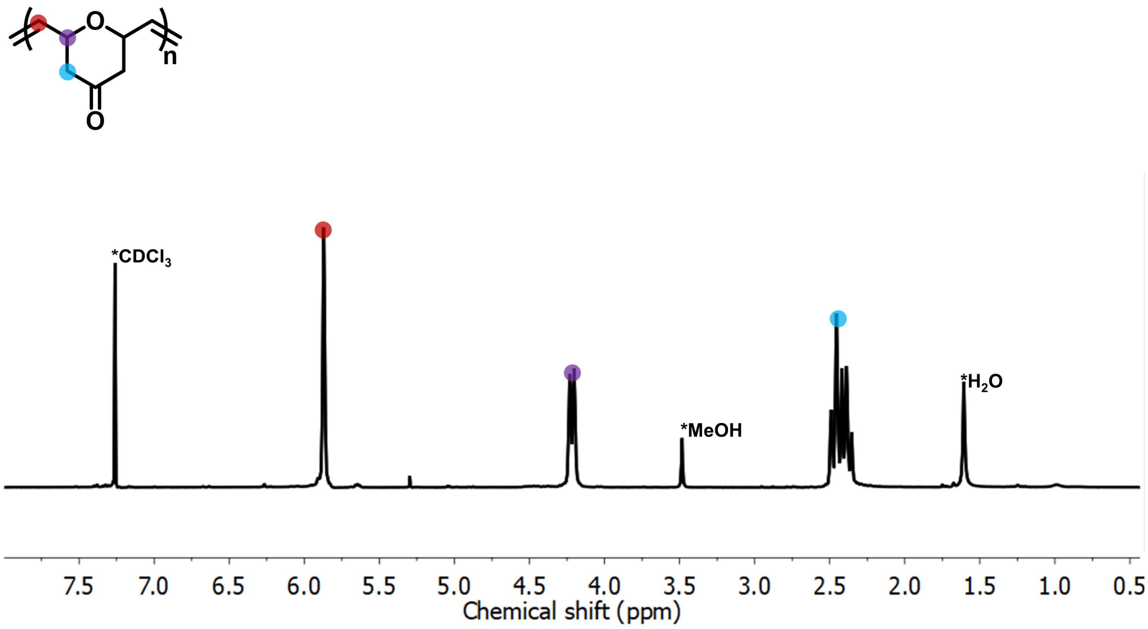

Figure S15. ^{1}H NMR spectrum of P1 in $CDCl_3$ at room temperature.

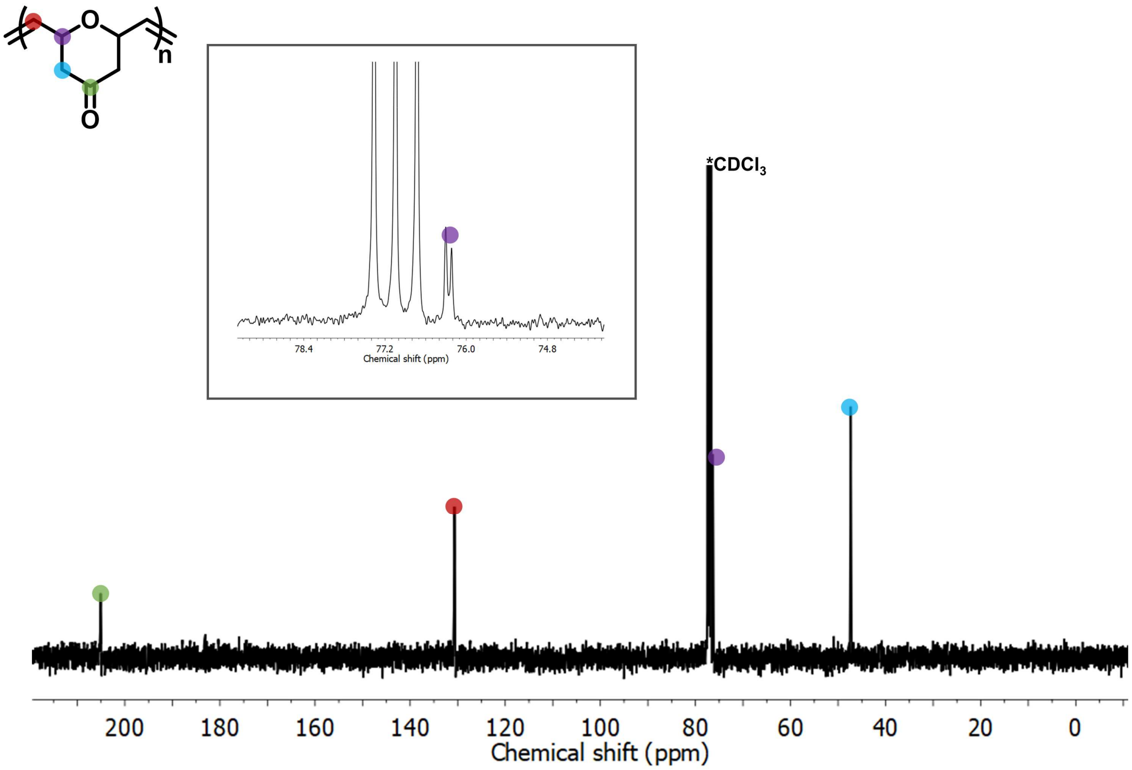

Figure S16. ^{13}C NMR spectrum of P1 in $CDCl_3$ at room temperature.

Table S6. Polymer information for P1

Entry	M1/G3	$[M]_0$ (mM)	Temperature (°C)	Time (h)	Conv. (%)	$M_{n,SEC}$ (kDa)	$Đ$
**1**	60	300	20	12	74.2	3.0	1.51
**2**	100	300	20	12	76.4	4.6	1.52
**3**	150	300	20	12	70.8	6.5	1.43
**4**	60	300	0	12	87.4	4.1	1.65
**5**	100	300	0	12	88.9	6.0	1.79
**6**	150	300	0	12	88.0	8.7	1.80

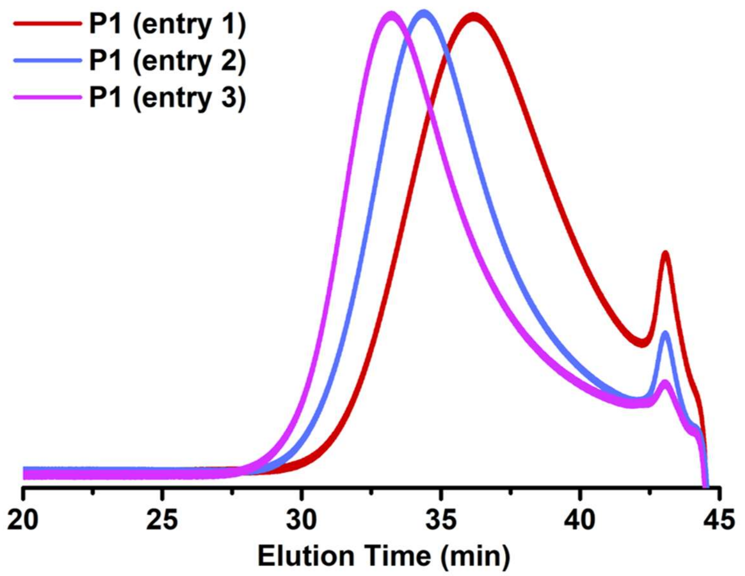

Figure S17. SEC traces of P1 (entries 1–3 in Table S2). $CHCl_3$ was used as the mobile phase.

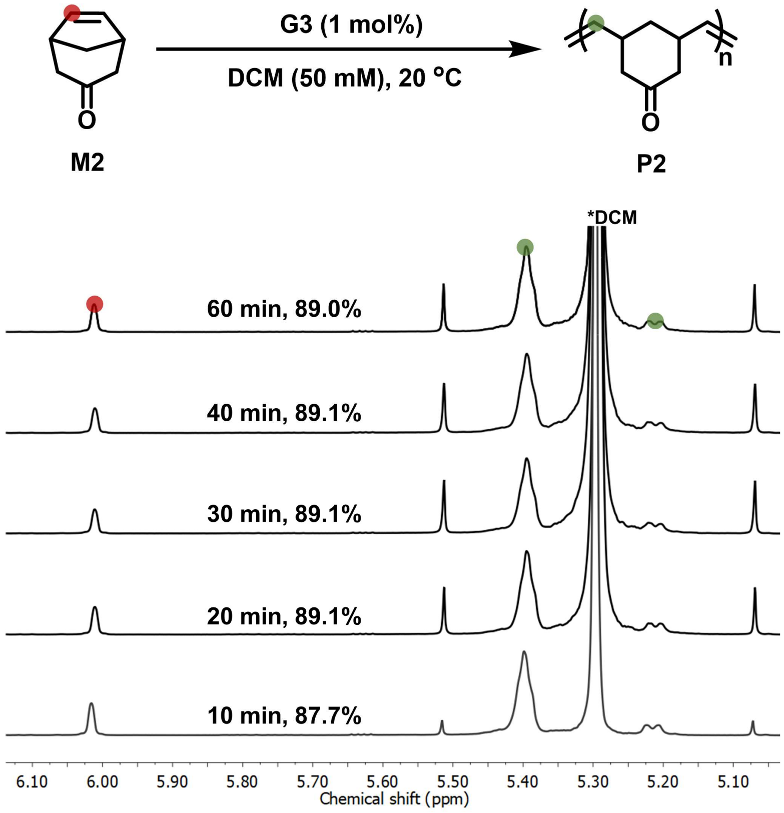

Figure S18. Partial ^{1}H NMR spectra for the polymerization kinetics of M2 (50 mM) in DCM at 20 °C. The polymerization reached equilibrium within 20 min.

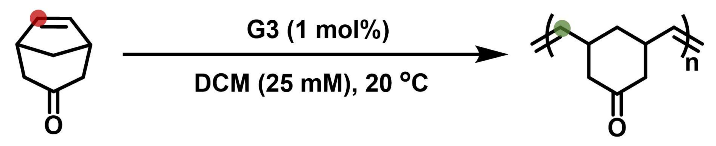

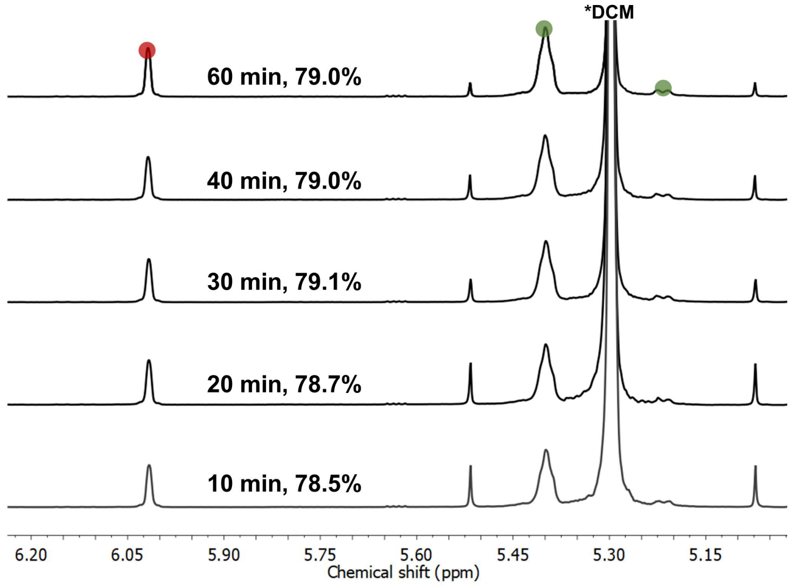

Figure S19. Partial ^{1}H NMR spectra for the polymerization kinetics of M2 (25 mM) in DCM at 20 °C. The polymerization reached equilibrium within 30 min.

Table S7. Polymerization kinetics of M2 in DCM at 20 °C

Entry	M2/G3	$[M]_0$ (mM)	Temperature (°C)	Time (min)	Conv. (%)
1	100	50	20	10	87.7
2	100	50	20	20	89.1
3	100	50	20	30	89.1
4	100	50	20	40	89.1
5	100	50	20	60	89.0
6	100	25	20	10	78.5
7	100	25	20	20	78.7
8	100	25	20	30	79.1
9	100	25	20	40	79.0
10	100	25	20	60	79.0

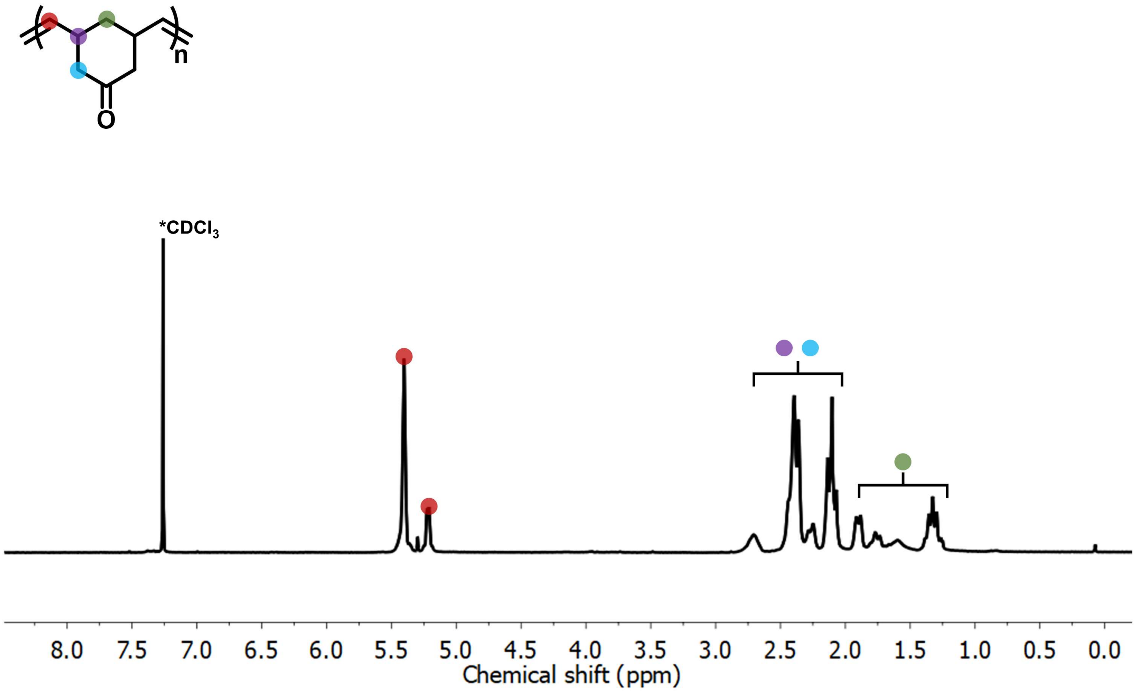

Figure S20. ^{1}H NMR spectrum of P2 in $CDCl_3$ at room temperature.

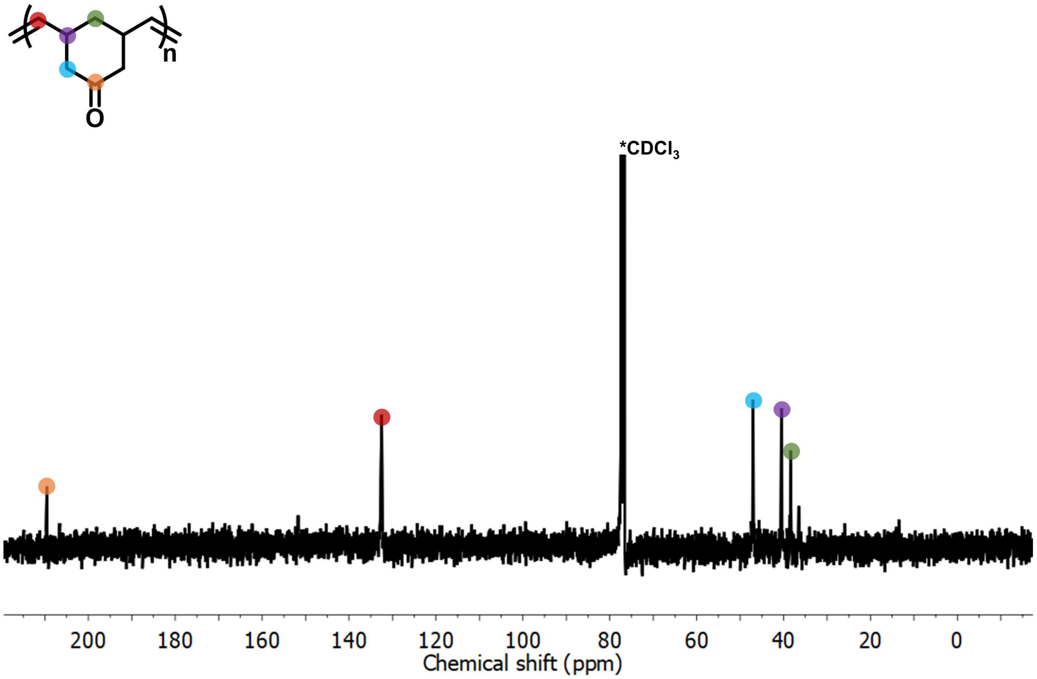

Figure S21. ^{13}C NMR spectrum of P2 in $CDCl_3$ at room temperature.

Table S8. Polymer information for P2

Entry	M2/G3	$[M]_0$ (mM)	Temperature (°C)	Time (h)	Conv. (%)	$M_{n,SEC}$ (kDa)	*Đ*
**1**	60	300	20	12	97.5	5.8	1.94
**2**	100	25	20	12	79.0	N.D.	N.D.
**3**	100	50	20	12	89.3	N.D.	N.D.
**4**	100	100	20	12	94.4	N.D.	N.D.
**5**	100	300	20	12	97.9	9.4	1.95
**6**	100	1000	20	12	>99	N.D.	N.D.
**7**	150	300	20	12	98.5	13.4	1.83

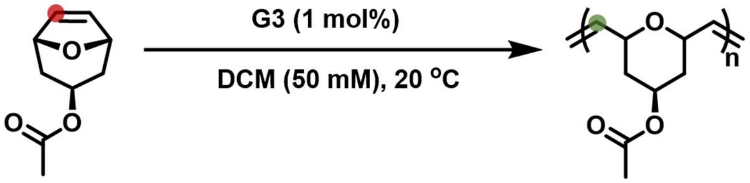

M3 P3

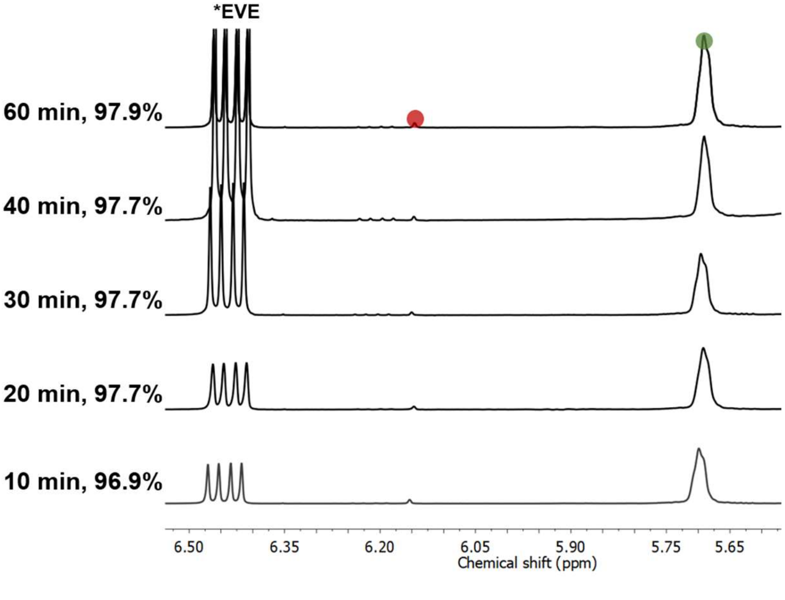

Figure S22. Partial ^{1}H NMR spectra for the polymerization kinetics of M3 (50 mM) in DCM at 20 °C. The polymerization reached equilibrium within 20 min.

Table S9. Polymerization kinetics of M3 at 20 °C

Entry	M3/G3	$[M]_0$ (mM)	Temperature (°C)	Time (min)	Conv. (%)
1	100	50	20	10	96.9
2	100	50	20	20	97.7
3	100	50	20	30	97.7
4	100	50	20	40	97.7
5	100	50	20	60	97.9

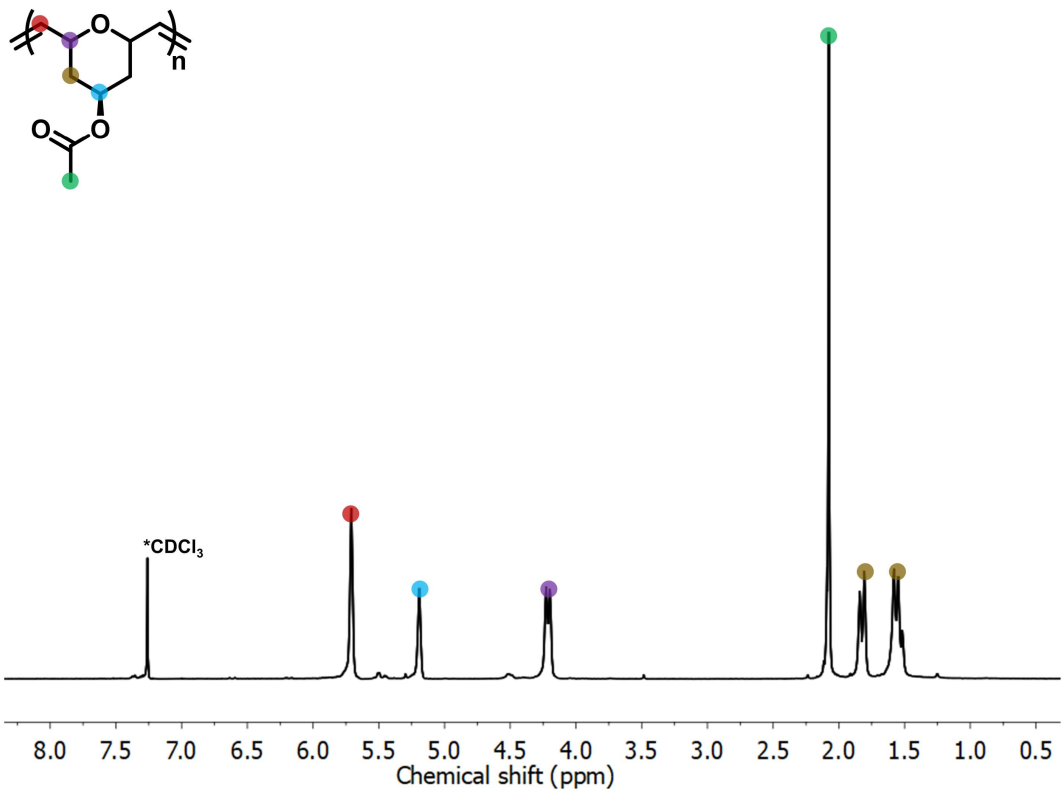

Figure S23. ^{1}H NMR spectrum of P3 in $CDCl_3$ at room temperature.

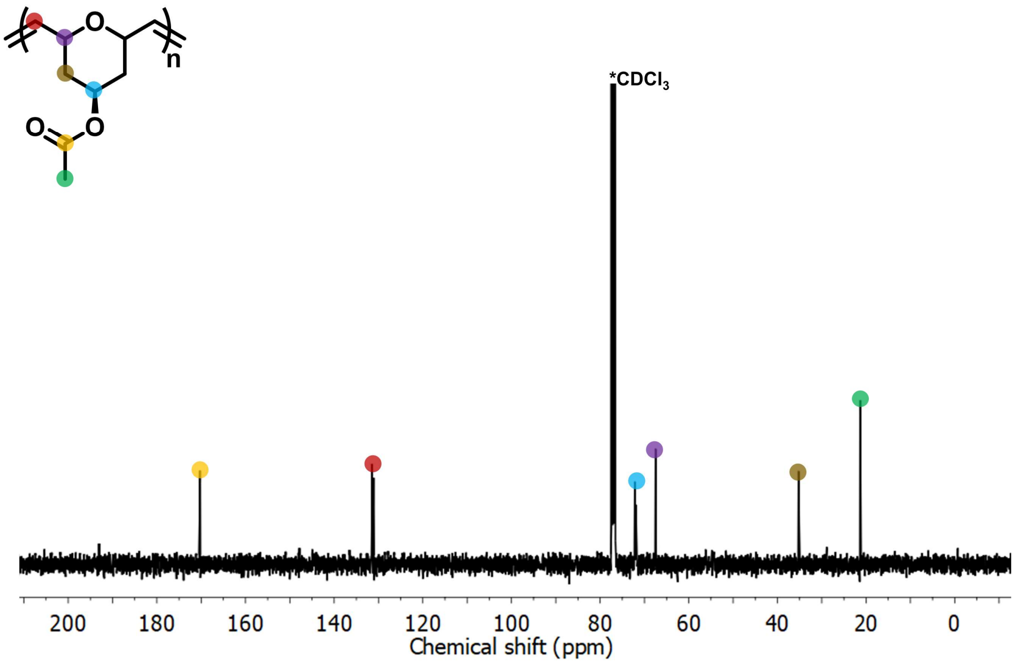

Figure S24. ^{13}C NMR spectrum of P3 in $CDCl_3$ at room temperature.

Table S10. Polymer information for P3

Entry	M3/G3	$[M]_0$ (mM)	Temperature (°C)	Time (h)	Conv. (%)	$M_{n,SEC}$ (kDa)	Đ
**1**	60	300	20	12	>99	15.1	1.05
**2**	180	300	20	12	>99	57.4	1.12
**3**	120	50	20	12	>98	29.5	1.10
**4**	180	50	20	12	>97	53.0	1.04
**5**	60	20	20	12	>94	14.3	1.09
**6**	500	50	20	12	>98	113	1.16

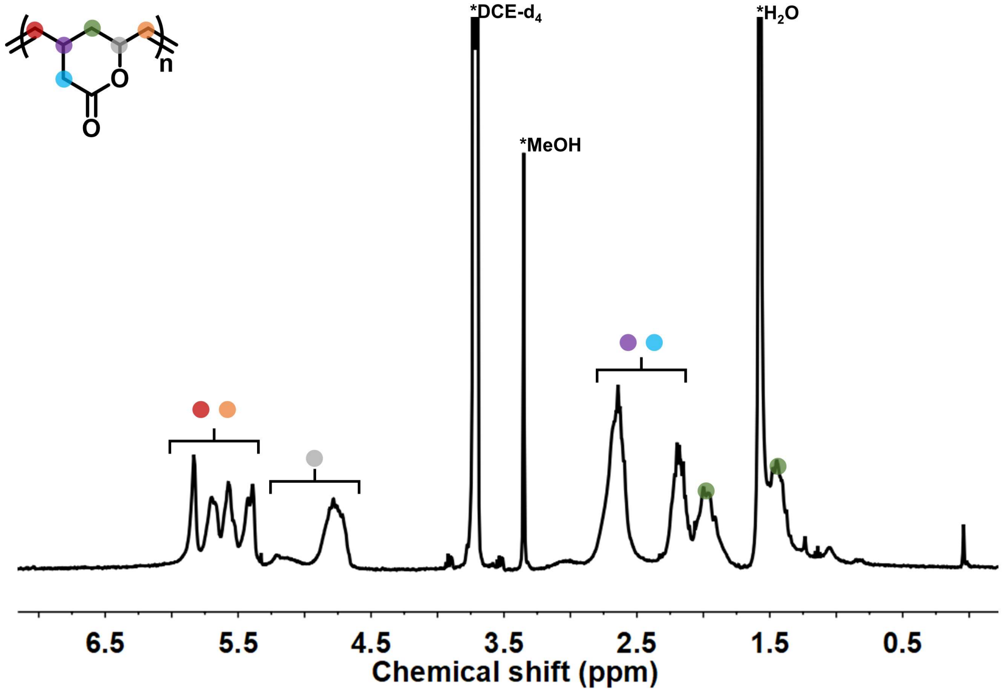

Figure S25. ^{1}H NMR spectrum of P4 in DCE-d4 at room temperature.

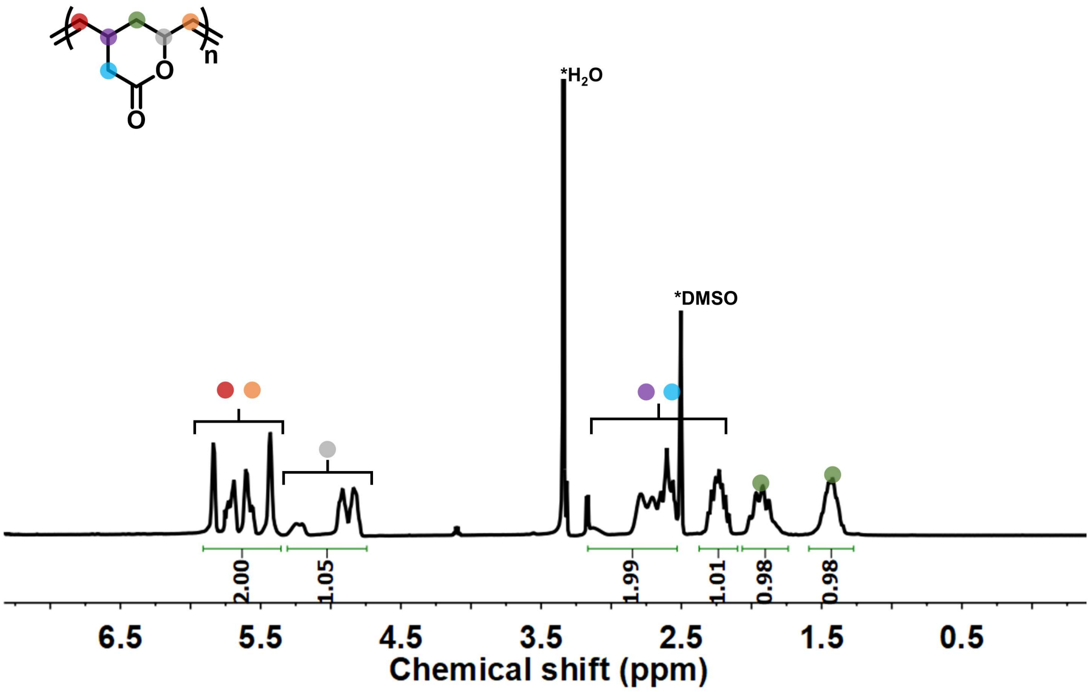

Figure S26. ^{1}H NMR spectrum of P4 in DMSO-d6 at room temperature.

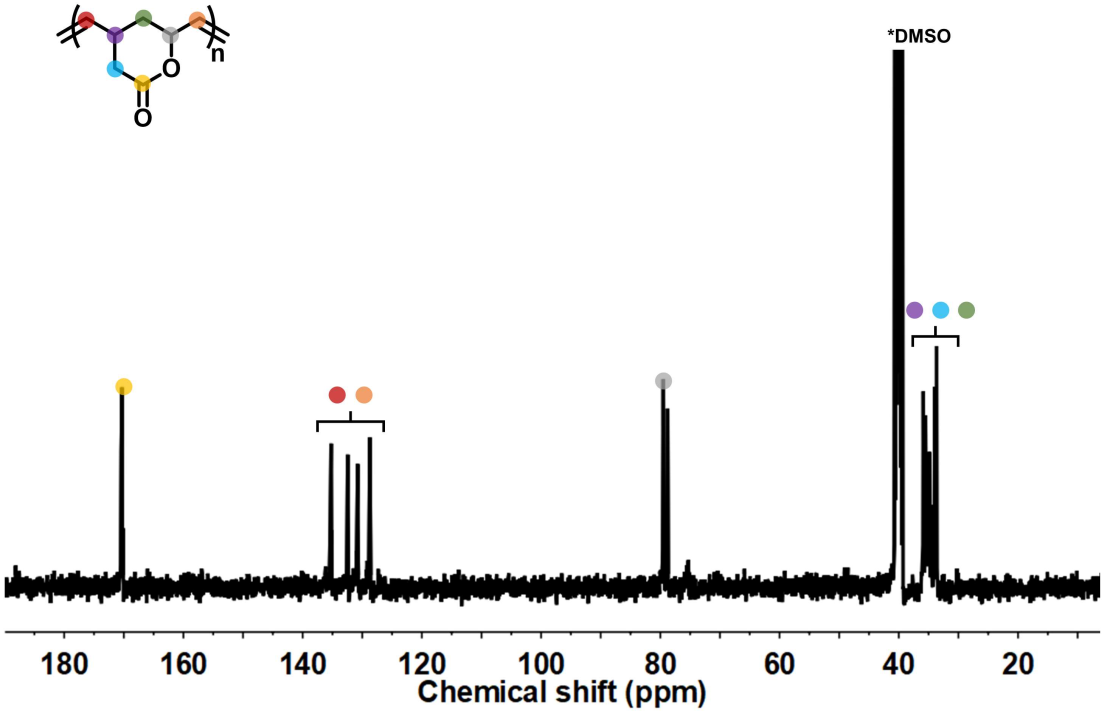

Figure S27. ^{13}C NMR spectrum of P4 in DMSO-d_6 at room temperature.

Table S11. Polymer information for P4

Entry	M4/G3	$[M]_0$ (mM)	Temperature (°C)	Time (h)	Conv. (%)	$M_{n,SEC}$ (kDa)	*Đ*
**1**	100	150	20	12	95	13.8	1.13
**2**	150	150	20	12	93	19.3	1.36
**3**	500	150	20	12	97	47.5	1.21

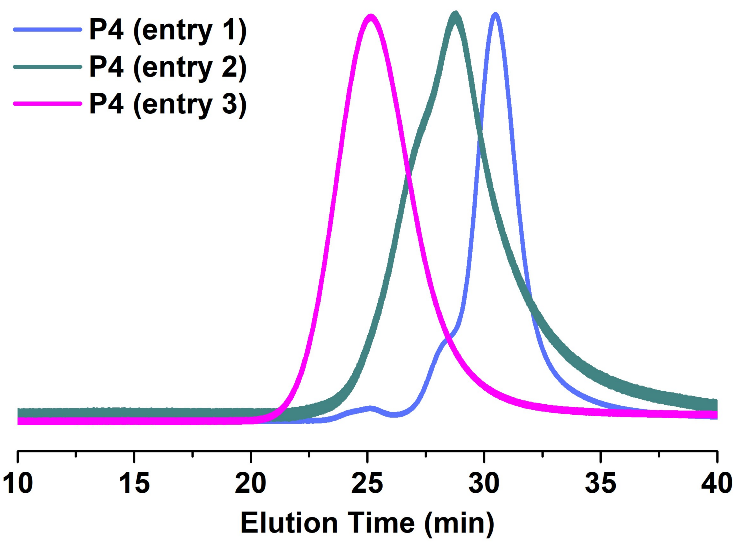

Figure S28. SEC traces of P4 (entries 1–3 in Table S7). DMF was used as the mobile phase.

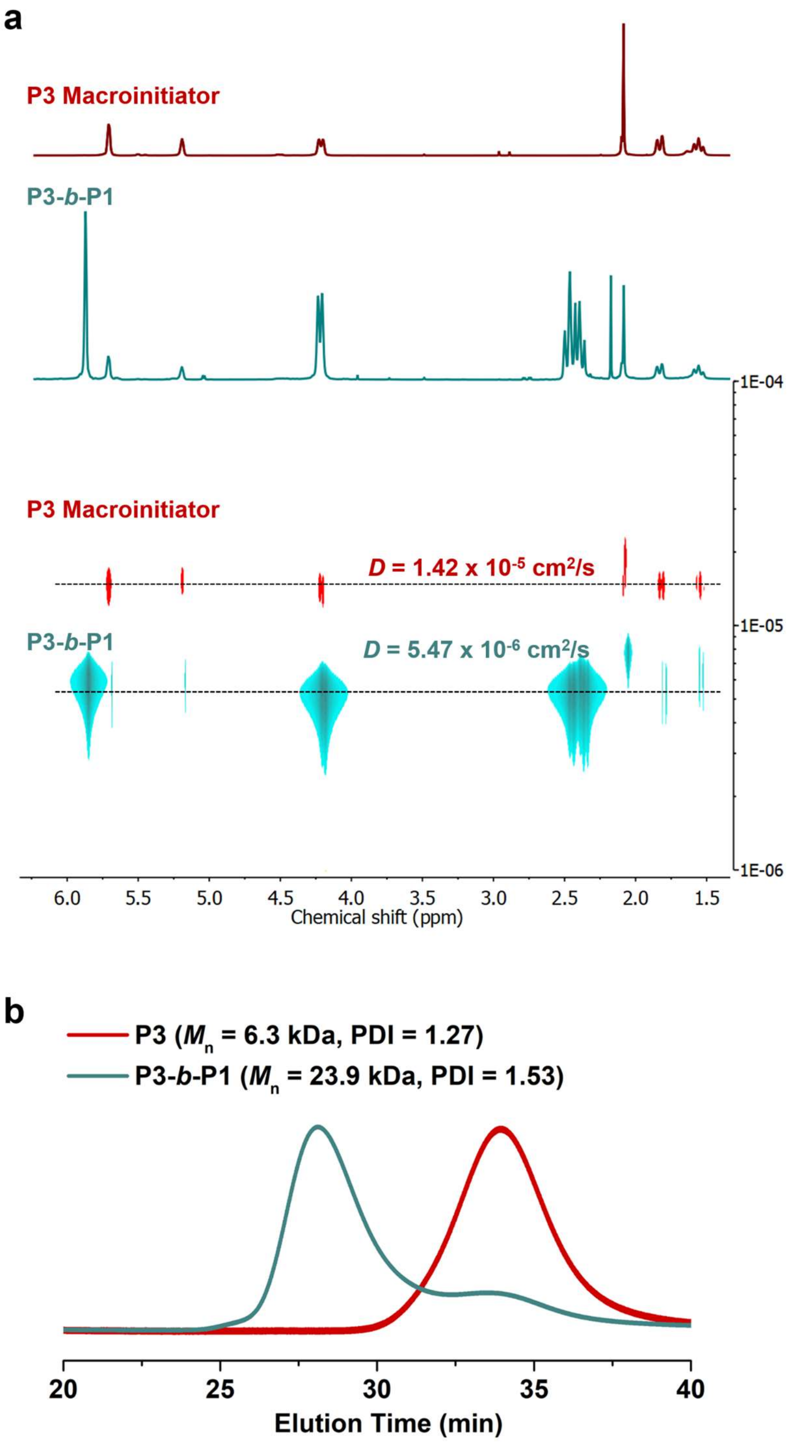

Figure S29. (**a**) DOSY NMR spectra of P3-*b*-P1 (Green line) and P3 macroinitiator (red line) in $CDCl_3$ at room temperature. After chain extension, P3 and P1 segments share the same diffusion coefficient (5.47×10^{-6} cm^2/s), which is smaller than that of P3 macroinitiator (1.42×10^{-5} cm^2/s), confirming that P1 is chemically bound to P3 rather than being physically mixed. (**b**) SEC traces of P3 macroinitiator and diblock copolymer P3-*b*-P1. $CHCl_3$ was used as the mobile phase.

Table S12. Depolymerization study of P1

Entry	Polymer	G2 loading	[Olefin] (mM)	Temperature (°C)	Time (h)	Conv. (%)
1	P1a	5 mol%	20	55	12	99
2	P1b	5 mol%	20	55	12	99
3	P1c	5 mol%	20	20	12	30
4	P1c	5 mol%	20	35	12	81
5	P1c	5 mol%	20	55	12	99
6	P1c	2 mol%	20	55	12	75
7	P1c	5 mol%	50	55	12	99
8	P1c	5 mol%	100	55	12	99
9	P1c	5 mol%	200	55	12	99
10	P1c	5 mol%	300	55	12	65

Note: P1a, P1b, and P1c correspond to entries 4–6 in Table S6, respectively.

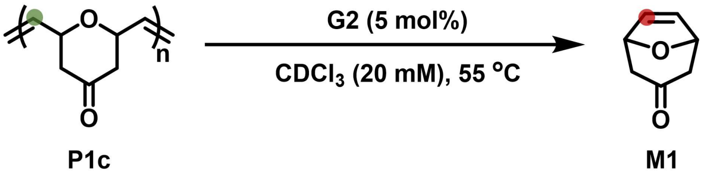

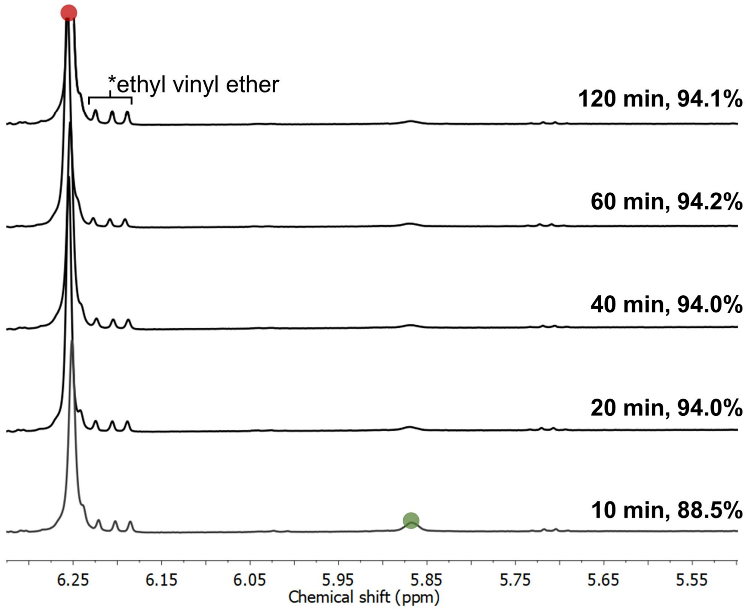

Figure S30. Partial ^{1}H NMR spectra for the depolymerization kinetics study of P1c in $CDCl_3$. The depolymerization reached equilibrium within 20 min.

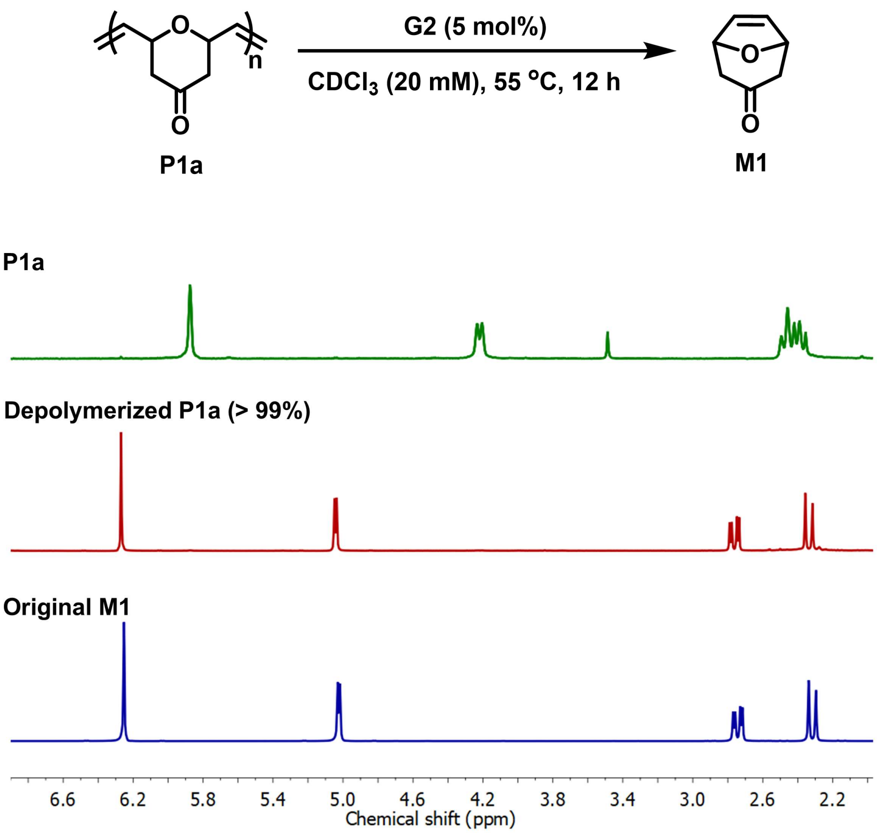

Figure S31. ^{1}H NMR spectra of P1a, depolymerized P1a, and the original monomer M1 in $CDCl_3$. Depolymerization was carried out using 5 mol% G2 at 20 mM olefins and 55 °C for 12 h (entry 1 in Table S12).

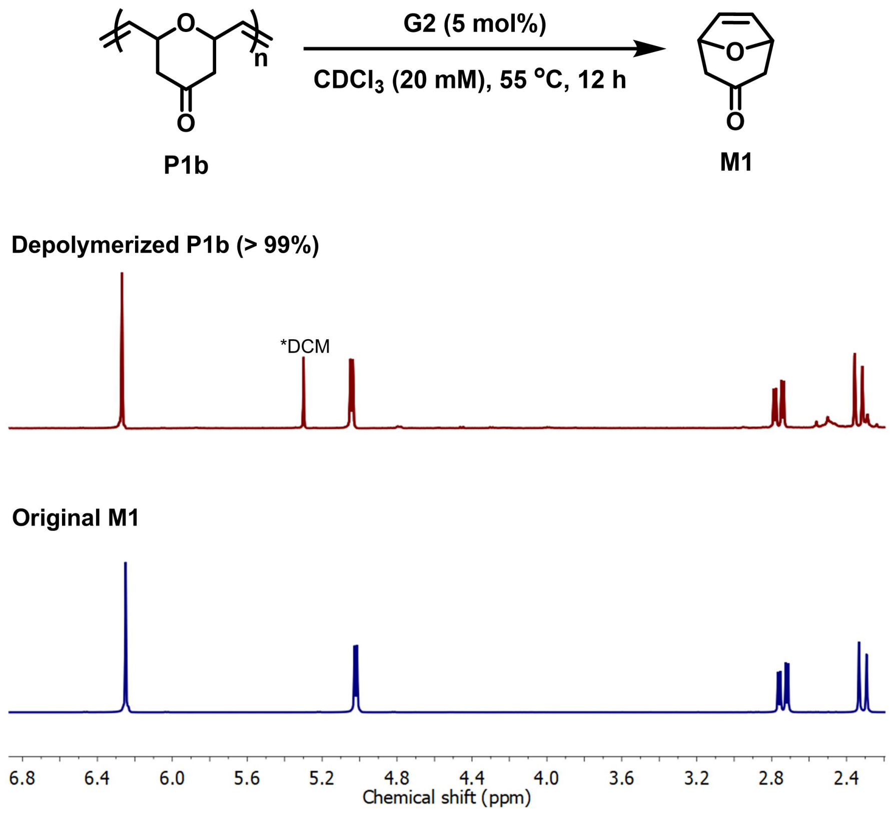

Figure S32. ^{1}H NMR spectra of depolymerized P1b and the original monomer M1 in $CDCl_3$. Depolymerization was carried out using 5 mol% G2 at 20 mM olefins and 55 °C for 12 h (entry 2 in Table S12).

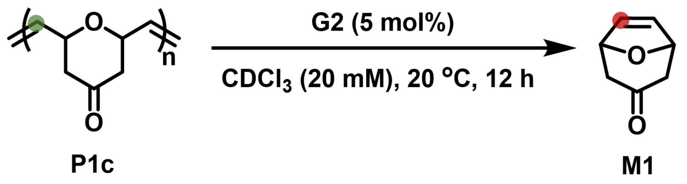

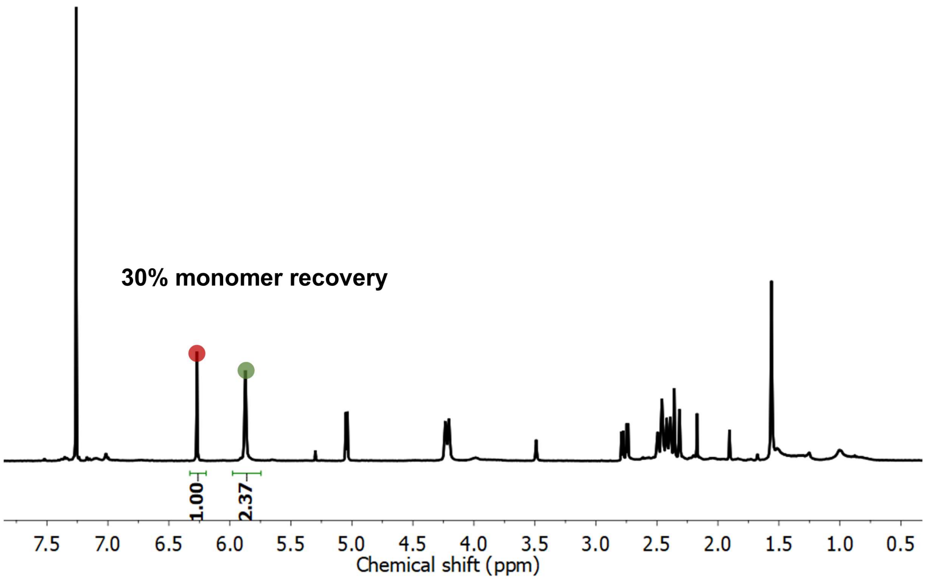

Figure S33. ^{1}H NMR spectrum of depolymerized P1c in $CDCl_3$. Depolymerization was carried out using 5 mol% G2 at 20 mM olefins and 20 °C for 12 h (entry 3 in Table S12).

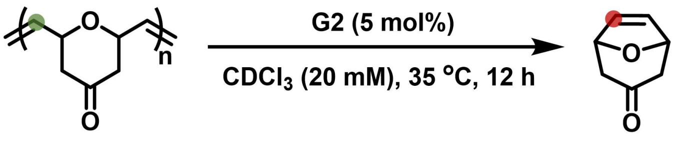

P1c **M1**

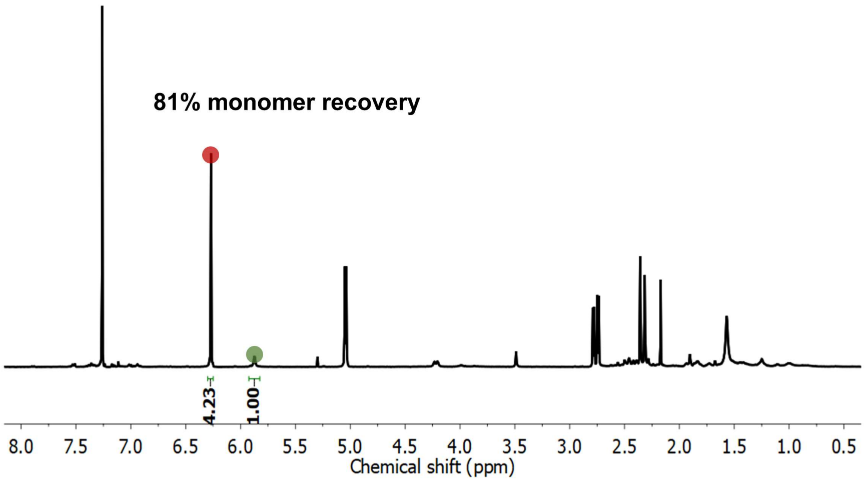

Figure S34. ^{1}H NMR spectrum of depolymerized P1c in $CDCl_3$. Depolymerization was carried out using 5 mol% G2 at 20 mM olefins and 35 °C for 12 h (entry 4 in Table S12).

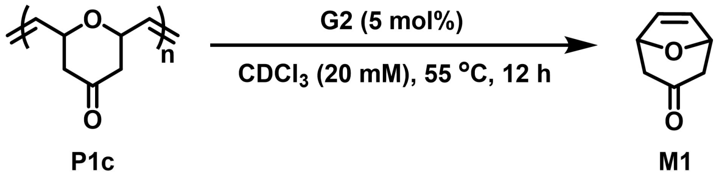

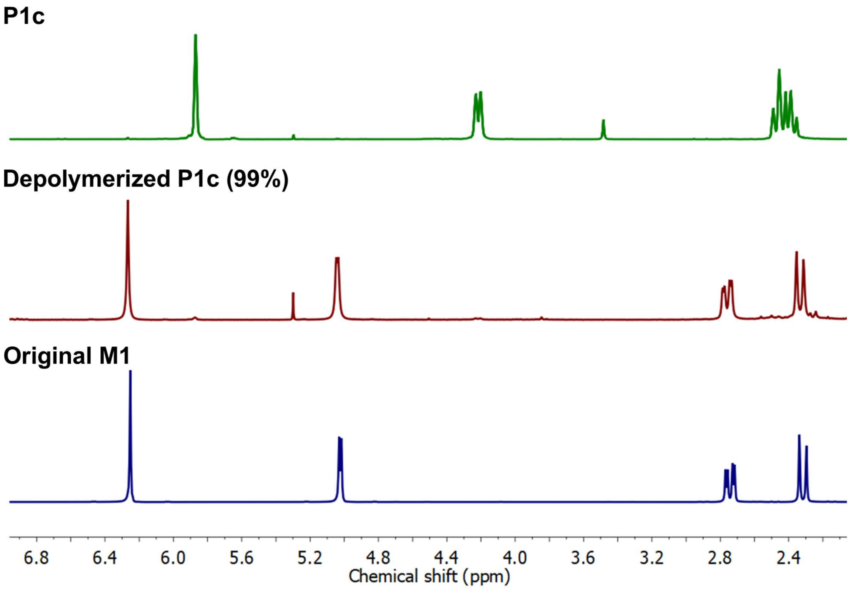

Figure S35. ^{1}H NMR spectra of P1c, depolymerized P1c, and the original monomer M1 in $CDCl_3$. Depolymerization was carried out using 5 mol% G2 at 20 mM olefins and 55 °C for 12 h (entry 5 in Table S12).

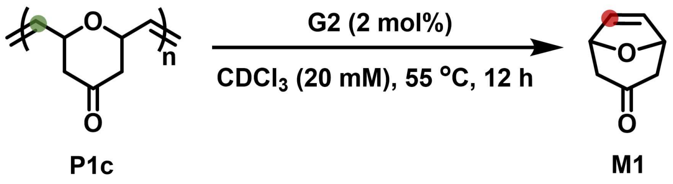

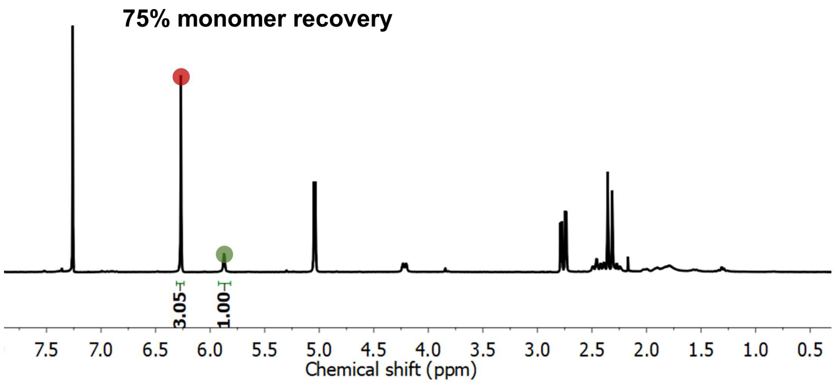

Figure S36. ^{1}H NMR spectrum of depolymerized P1c in $CDCl_3$. Depolymerization was carried out using 2 mol% G2 at 20 mM olefins and 55 °C for 12 h (entry 6 in Table S12).

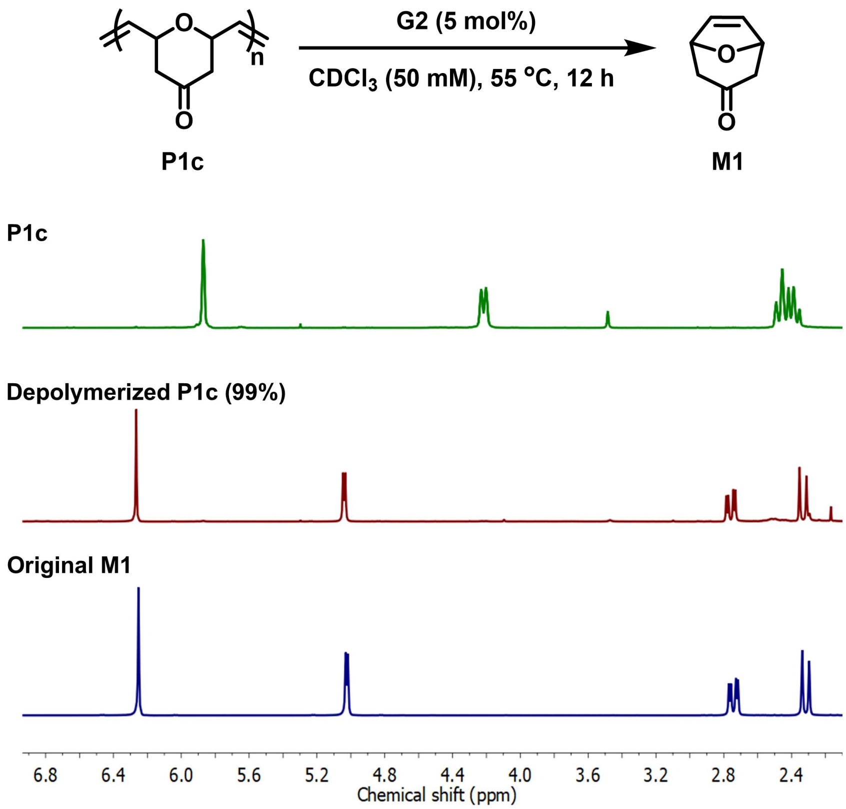

Figure S37. ^{1}H NMR spectra of P1c, depolymerized P1c, and the original monomer M1 in $CDCl_3$. Depolymerization was carried out using 5 mol% G2 at 50 mM olefins and 55 °C for 12 h (entry 7 in Table S12).

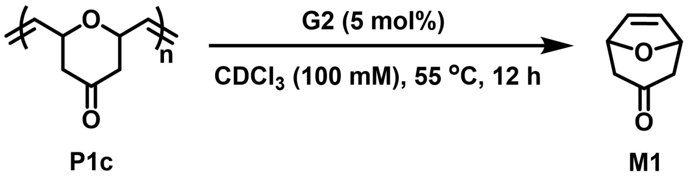

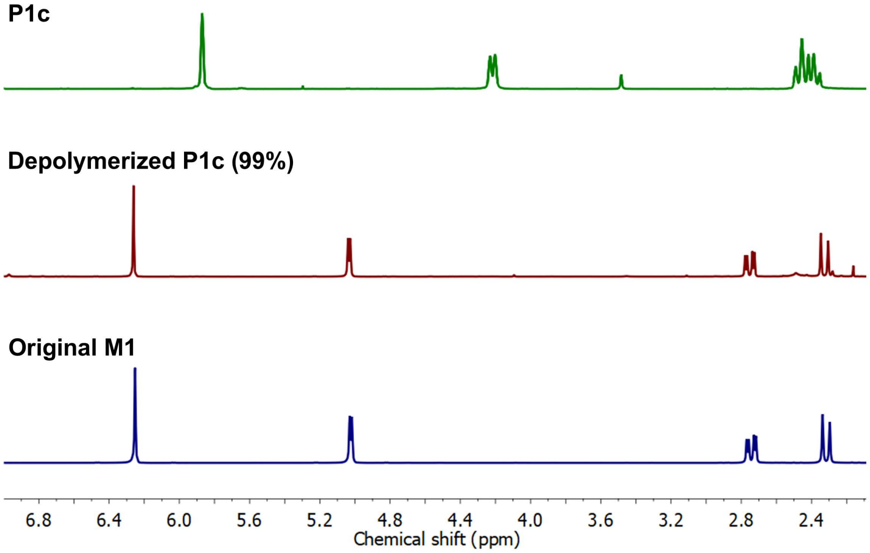

Figure S38. ^{1}H NMR spectra of P1c, depolymerized P1c, and the original monomer M1 in $CDCl_3$. Depolymerization was carried out using 5 mol% G2 at 100 mM olefins and 55 °C for 12 h (entry 8 in Table S12).

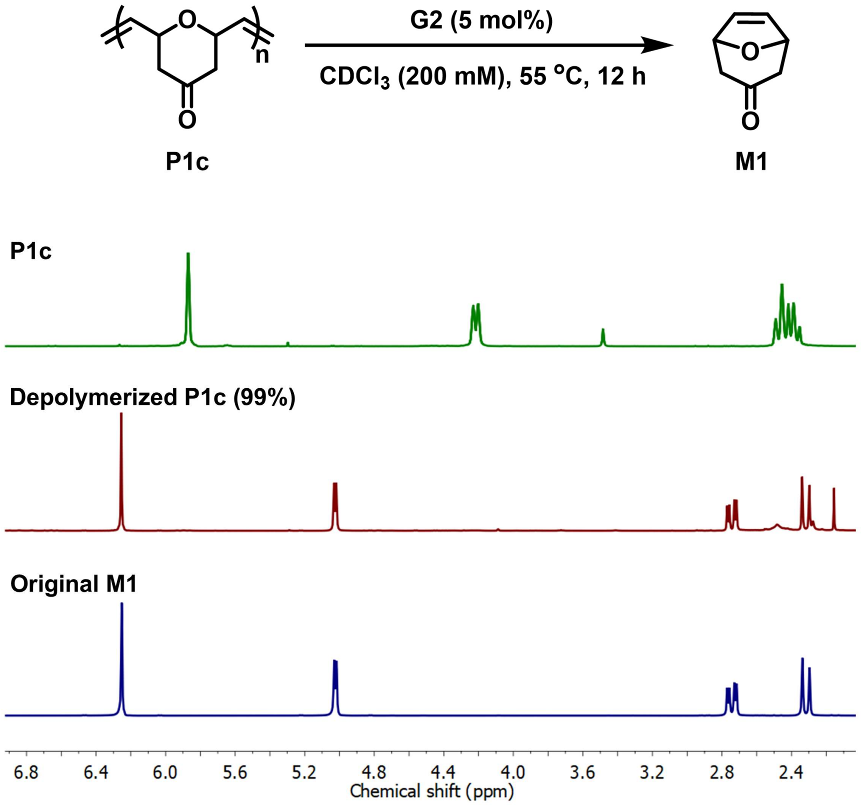

Figure S39. ^{1}H NMR spectra of P1c, depolymerized P1c, and the original monomer M1 in $CDCl_3$. Depolymerization was carried out using 5 mol% G2 at 200 mM olefins and 55 °C for 12 h (entry 9 in Table S12).

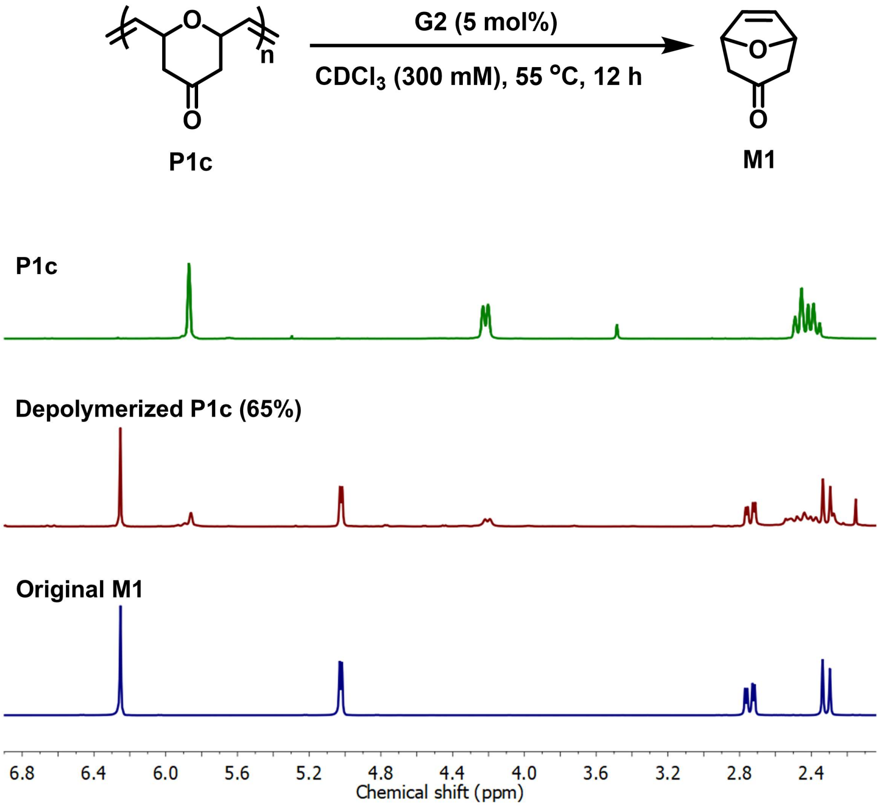

Figure S40. ^{1}H NMR spectra of P1c, depolymerized P1c, and the original monomer M1 in $CDCl_3$. Depolymerization was carried out using 5 mol% G2 at 300 mM olefins and 55 °C for 12 h (entry 10 in Table S12).

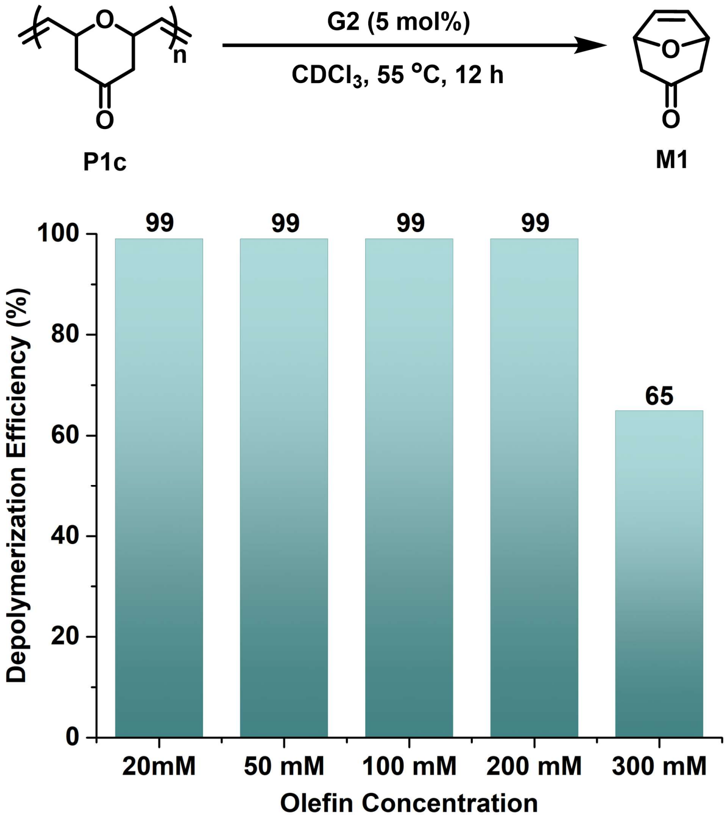

Figure S41. Depolymerization efficiencies of P1c at various concentrations (entries 5, 7–10 in Table S12).

Supplementary Table 13. Depolymerization study of P2

Entry	Polymer	G2 loading	[Olefin] (mM)	Temperature (°C)	Time (h)	Conv. (%)
1	P2	5 mol%	50	55	12	45
2	P2	5 mol%	20	20	12	6
3	P2	2 mol%	20	20	12	5
4	P2	2 mol%	20	55	12	28
5	P2	5 mol%	20	55	12	76
6	P2	5 mol%	10	55	12	40
7	P2	10 mol%	10	55	12	63
8	P2	5 mol%	5	55	12	22
9	P2	20 mol%	5	55	12	65

Note: P2 corresponds to entry 7 in Table S8.

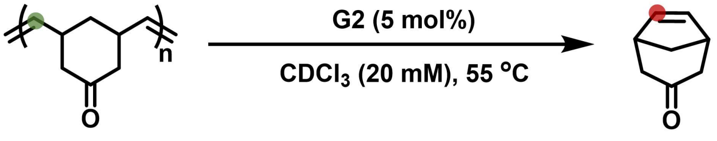

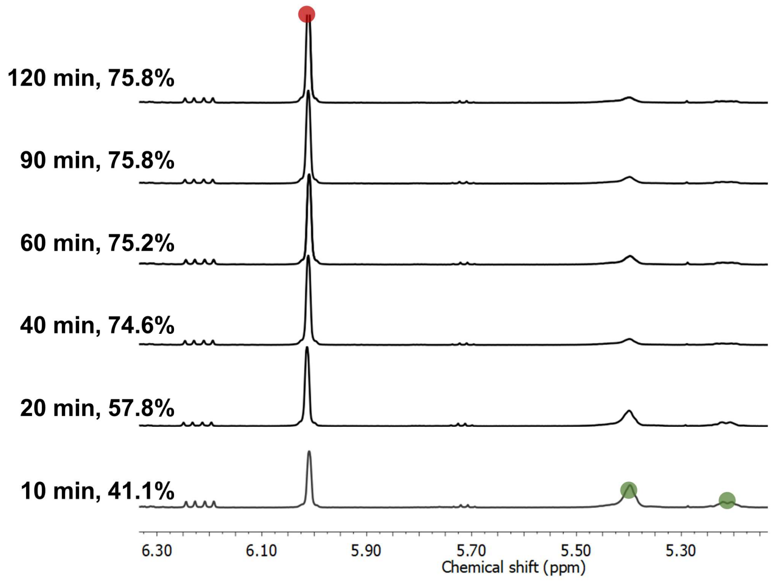

Figure S42. Partial ^{1}H NMR spectra for the depolymerization kinetics study of P2 in $CDCl_3$. The depolymerization reached equilibrium within 90 min.

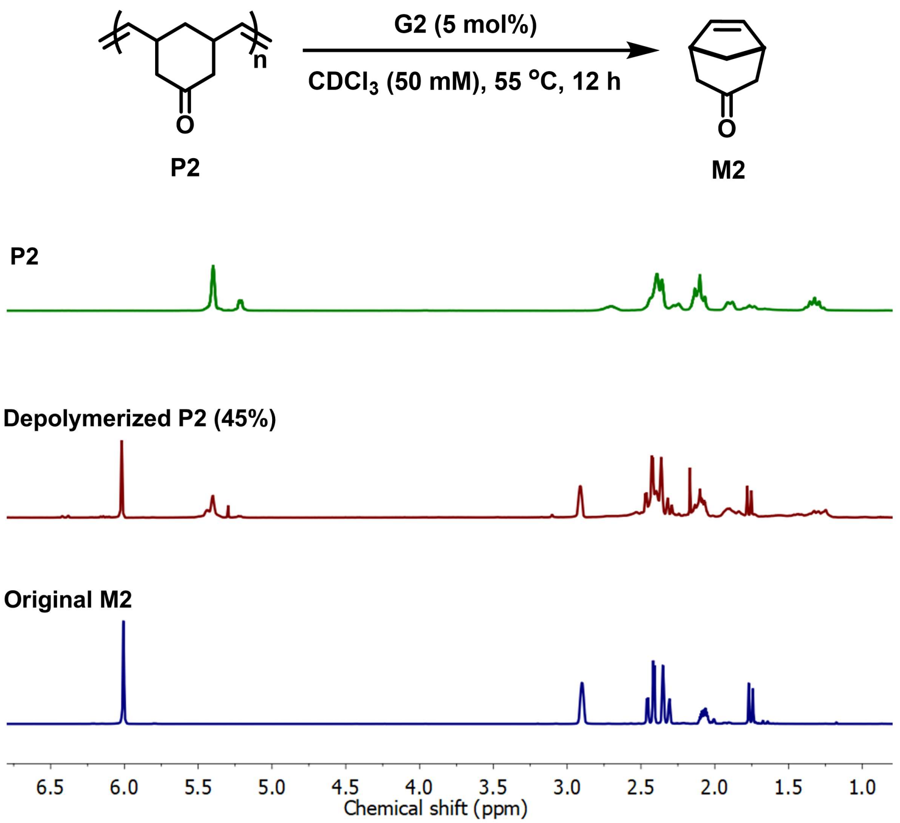

Figure S43. ^{1}H NMR spectra of P2, depolymerized P2, and the original monomer M2 in $CDCl_3$. Depolymerization was carried out using 5 mol% G2 at 50 mM olefins and 55 °C for 12 h (entry 1 in Table S13).

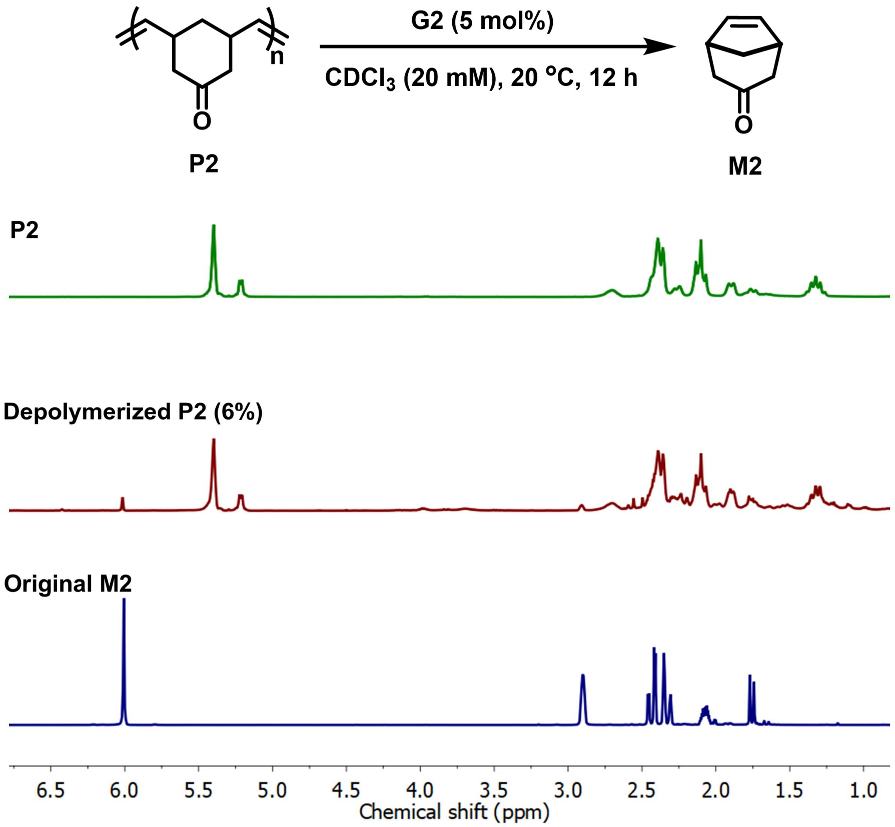

Figure S44. ^{1}H NMR spectra of P2, depolymerized P2, and the original monomer M2 in $CDCl_3$. Depolymerization was carried out using 5 mol% G2 at 20 mM olefins and 20 °C for 12 h (entry 2 in Table S13).

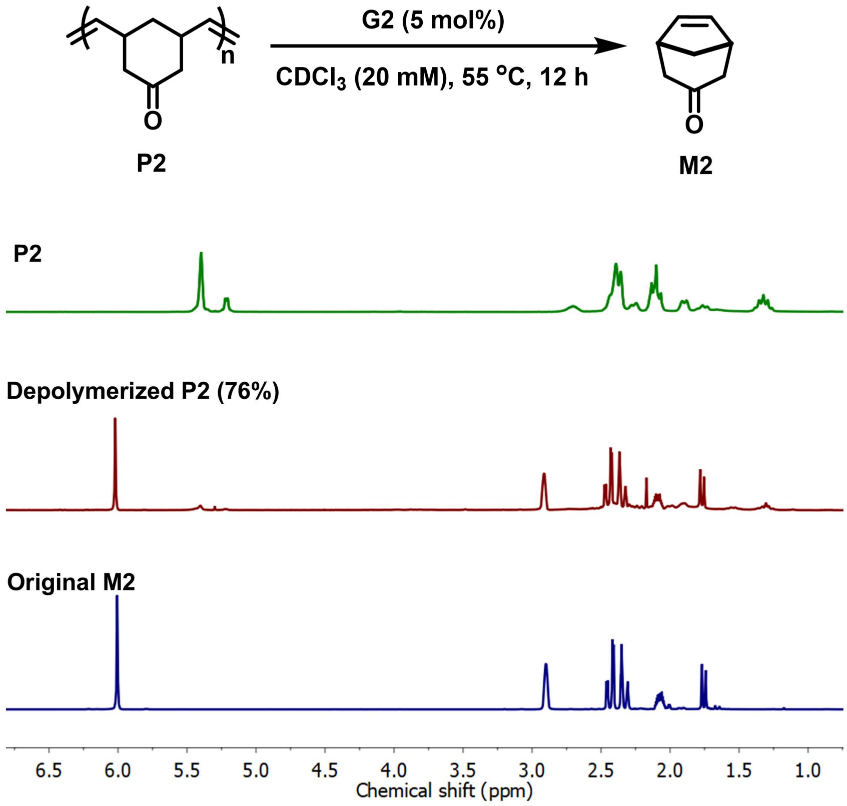

Figure S45. ^{1}H NMR spectra of P2, depolymerized P2, and the original monomer M2 in $CDCl_3$. Depolymerization was carried out using 5 mol% G2 at 20 mM olefins and 55 °C for 12 h (entry 5 in Table S13).

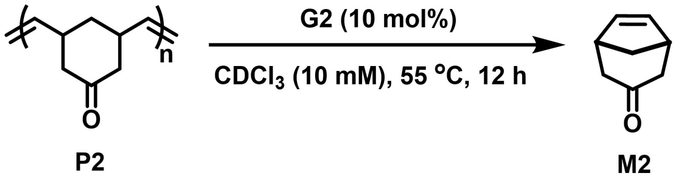

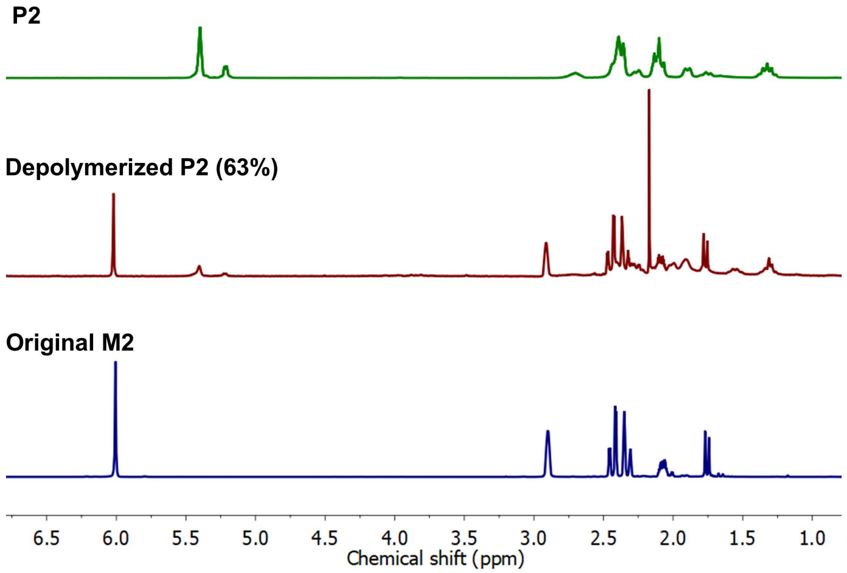

Figure S46. ^{1}H NMR spectra of P2, depolymerized P2, and the original monomer M2 in $CDCl_3$. Depolymerization was carried out using 10 mol% G2 at 10 mM olefins and 55 °C for 12 h (entry 7 in Table S13).

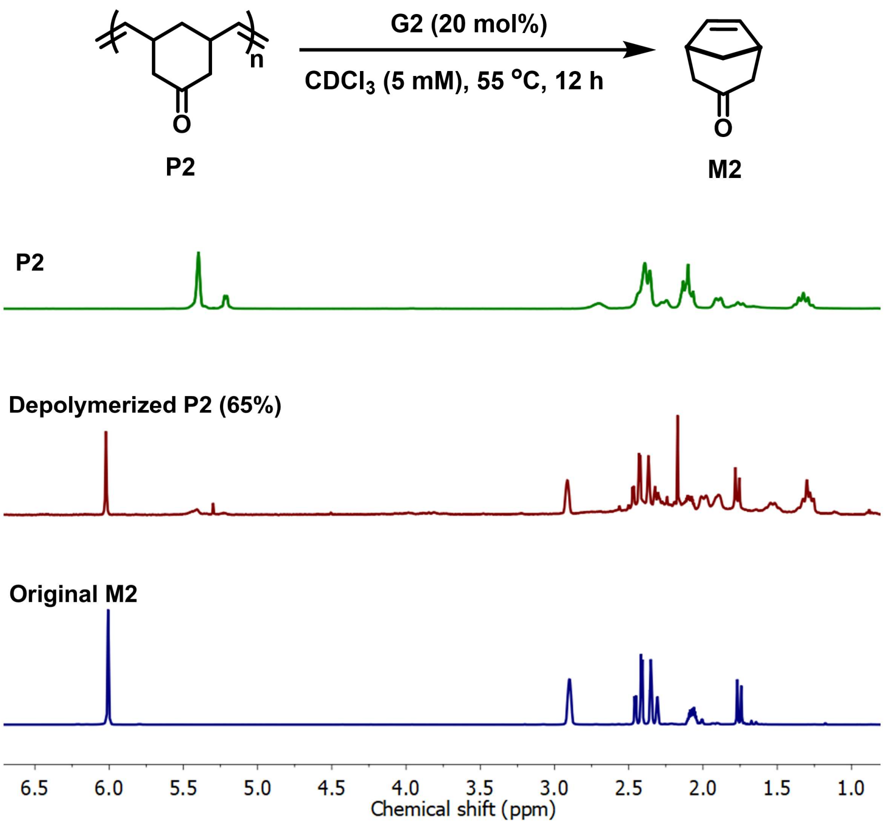

Figure S47. ^{1}H NMR spectra of P2, depolymerized P2, and the original monomer M2 in $CDCl_3$. Depolymerization was carried out using 20 mol% G2 at 5 mM olefins and 55 °C for 12 h (entry 9 in Table S13).

Table S14. Depolymerization study of P3

Entry	Polymer	G2 loading	[Olefin] (mM)	Temperature (°C)	Time (h)	Conv. (%)
1	P3a	5 mol%	20	55	12	31
2	P3a	5 mol%	10	55	12	35
3	P3a	5 mol%	5	55	12	70
4	P3b	5 mol%	20	55	12	33
5	P3b	5 mol%	10	55	12	53
6	P3b	5 mol%	5	55	12	74

Note: P3a and P3b correspond to entries 5 and 4 in Table S10, respectively.

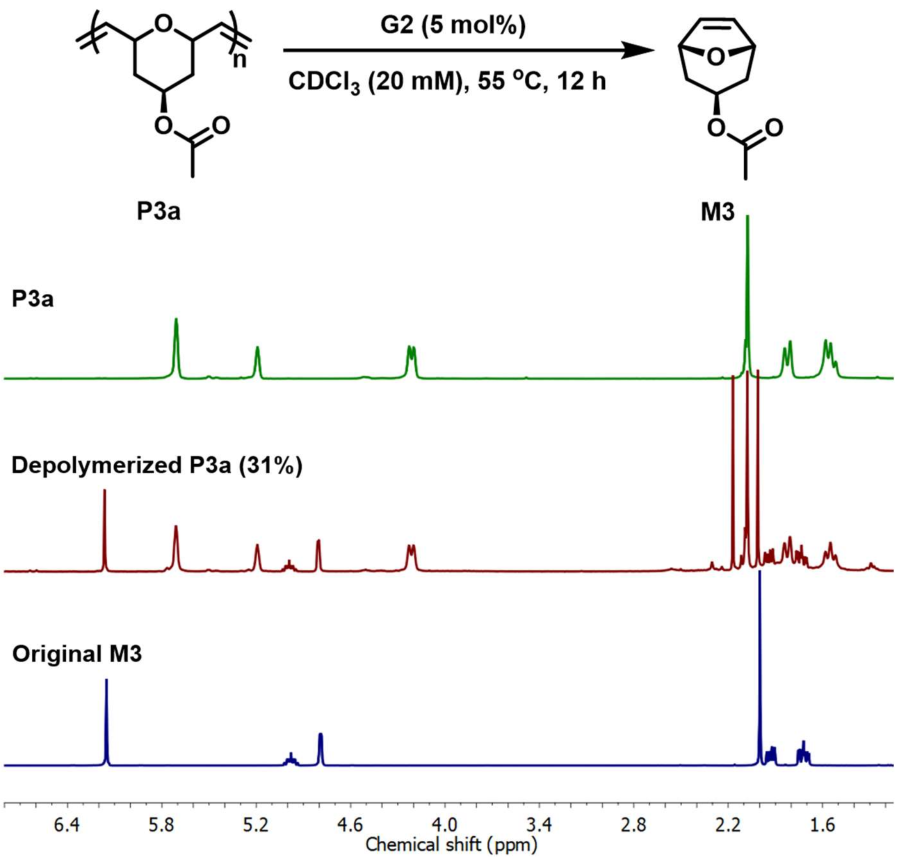

Figure S48. ^{1}H NMR spectra of P3a, depolymerized P3a, and the original monomer M3 in $CDCl_3$. Depolymerization was carried out using 5 mol% G2 at 20 mM olefins and 55 °C for 12 h (entry 1 in Table S14).

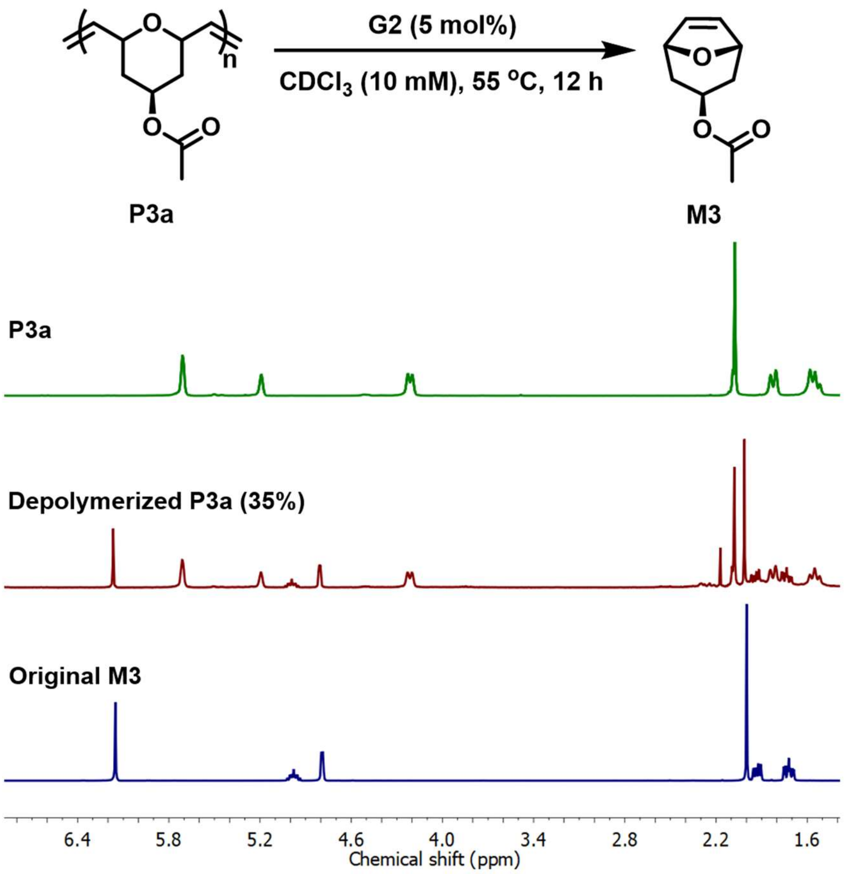

Figure S49. ^{1}H NMR spectra of P3a, depolymerized P3a, and the original monomer M3 in $CDCl_3$. Depolymerization was carried out using 5 mol% G2 at 10 mM olefins and 55 °C for 12 h (entry 2 in Table S14).

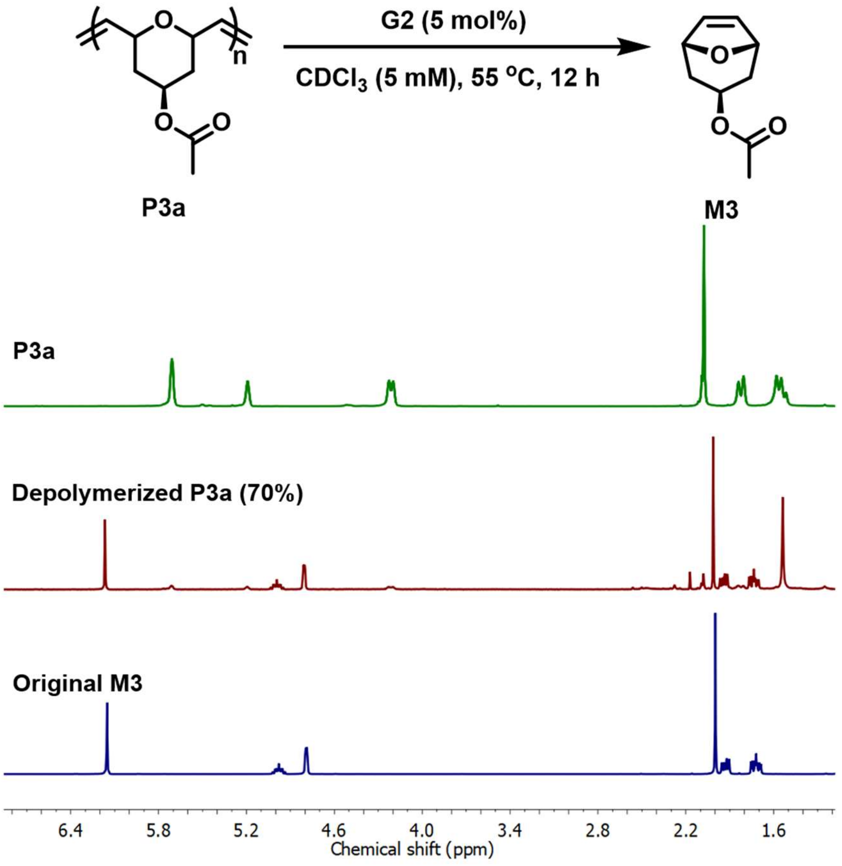

Figure S50. ^{1}H NMR spectra of P3a, depolymerized P3a, and the original monomer M3 in $CDCl_3$. Depolymerization was carried out using 5 mol% G2 at 5 mM olefins and 55 °C for 12 h (entry 3 in Table S14).

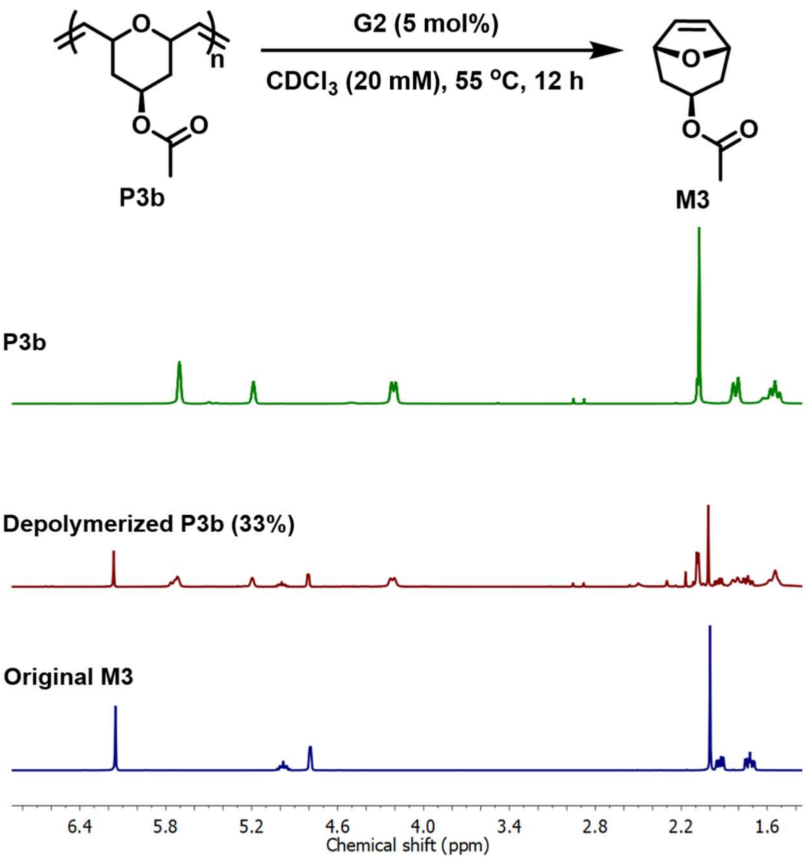

Figure S51. ^{1}H NMR spectra of P3b, depolymerized P3b, and the original monomer M3 in $CDCl_3$. Depolymerization was carried out using 5 mol% G2 at 20 mM olefins and 55 °C for 12 h (entry 4 in Table S14).

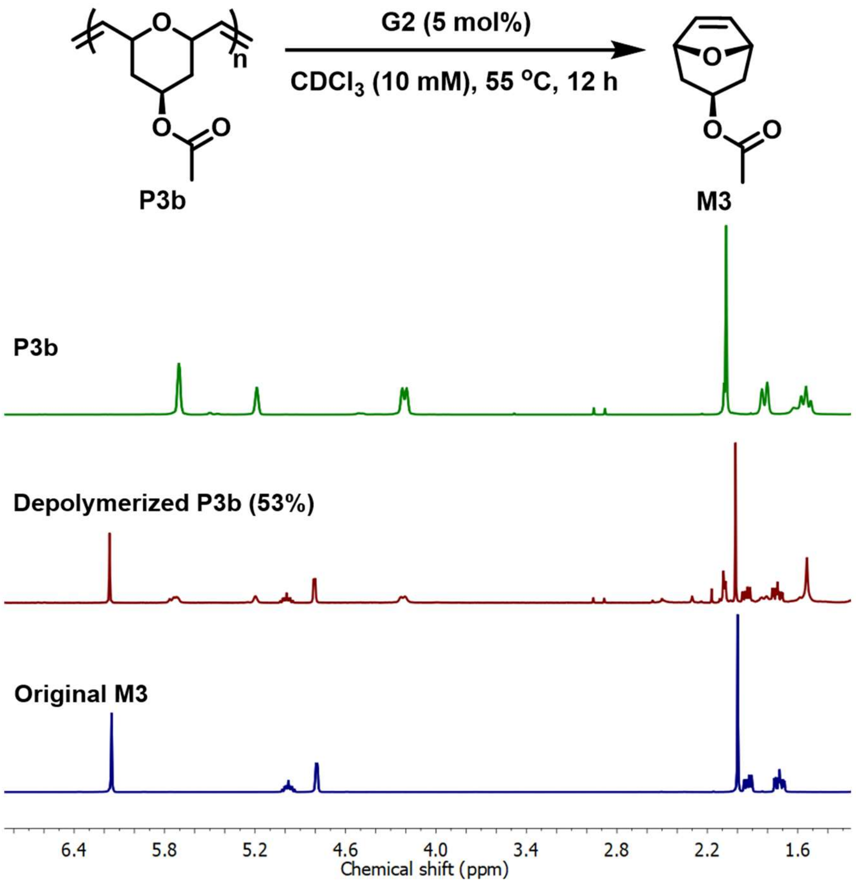

Figure S52. ^{1}H NMR spectra of P3b, depolymerized P3b, and the original monomer M3 in $CDCl_3$. Depolymerization was carried out using 5 mol% G2 at 10 mM olefins and 55 °C for 12 h (entry 5 in Table S14).

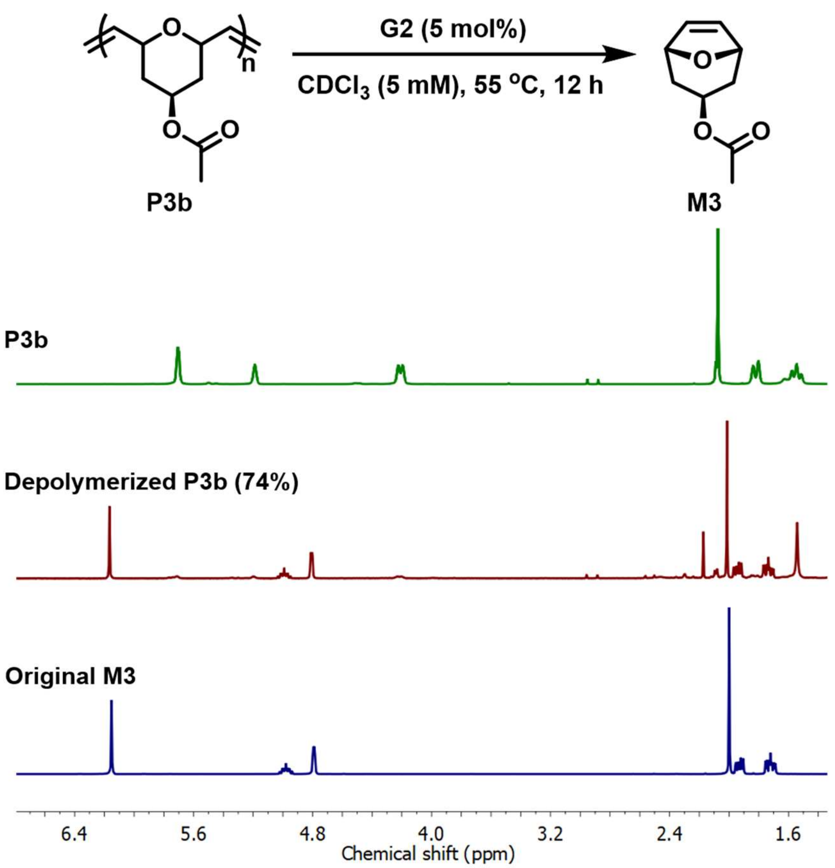

Figure S53. ^{1}H NMR spectra of P3b, depolymerized P3b, and the original monomer M3 in $CDCl_3$. Depolymerization was carried out using 5 mol% G2 at 5 mM olefins and 55 °C for 12 h (entry 6 in Table S14).

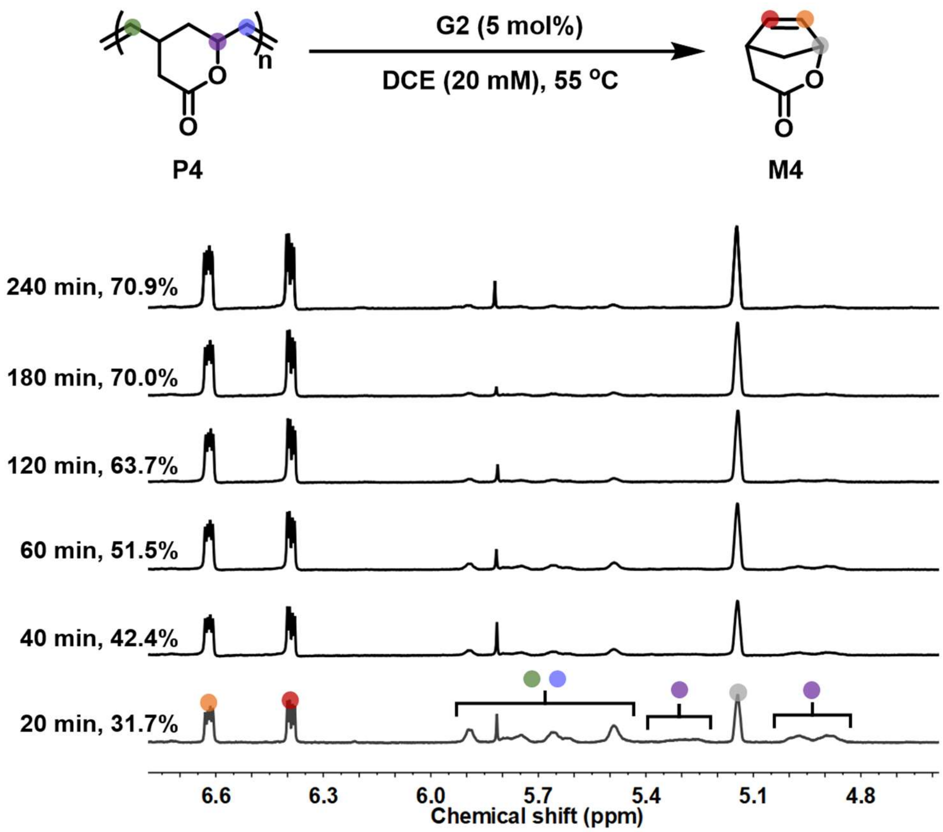

Figure S54. Partial ^{1}H NMR spectra for the depolymerization kinetics study of P4 (entry 2 in Table S7).

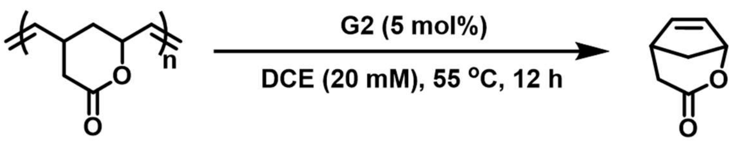

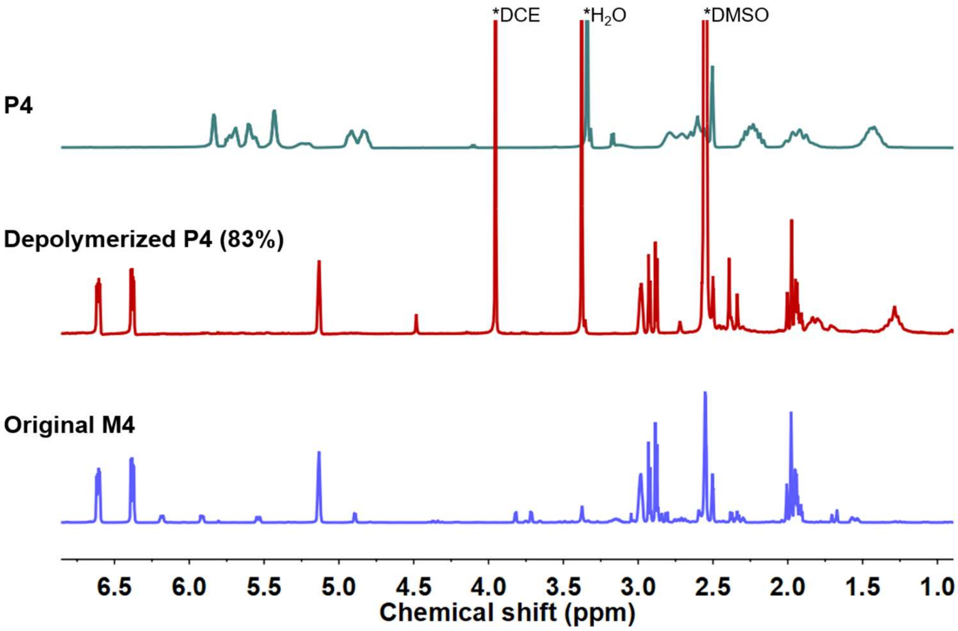

Figure S55. ^{1}H NMR spectra of P4 (entry 2 in Table S7), depolymerized P4, and the original monomer M4 in DMSO-d$_6$. Depolymerization was carried out using 5 mol% G2 at 20 mM olefins and 55 °C for 12 h.

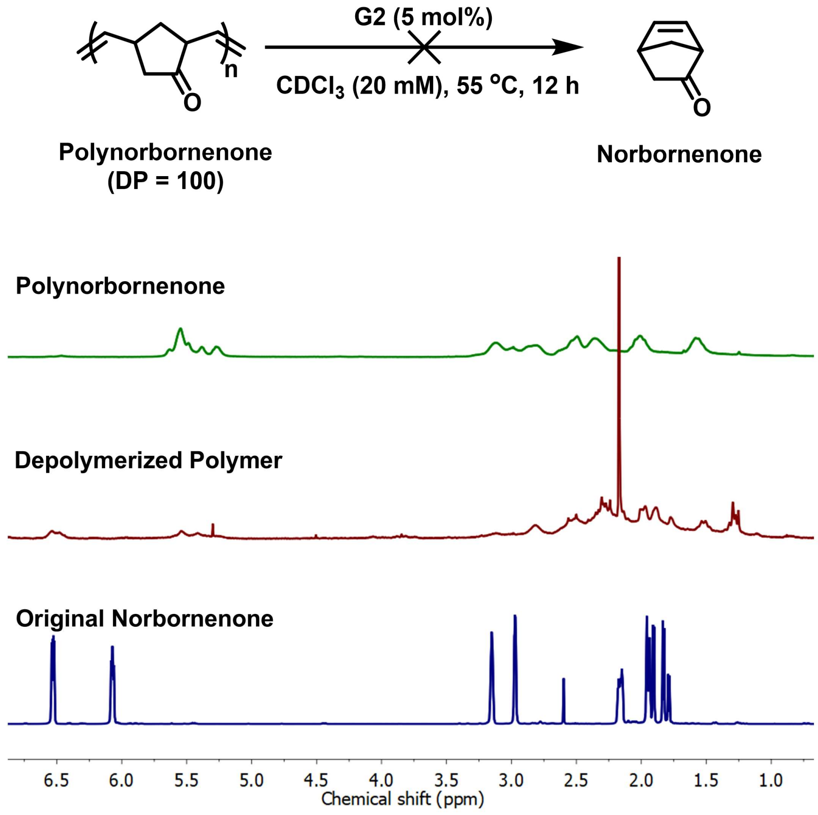

Figure S56. ^{1}H NMR spectra of polynorbornenone, depolymerized polynorbornenone, and the original monomer in $CDCl_3$. Depolymerization was carried out using 5 mol% G2 at 20 mM olefins and 55 °C for 12 h. No monomer was regenerated under the investigated depolymerization conditions.

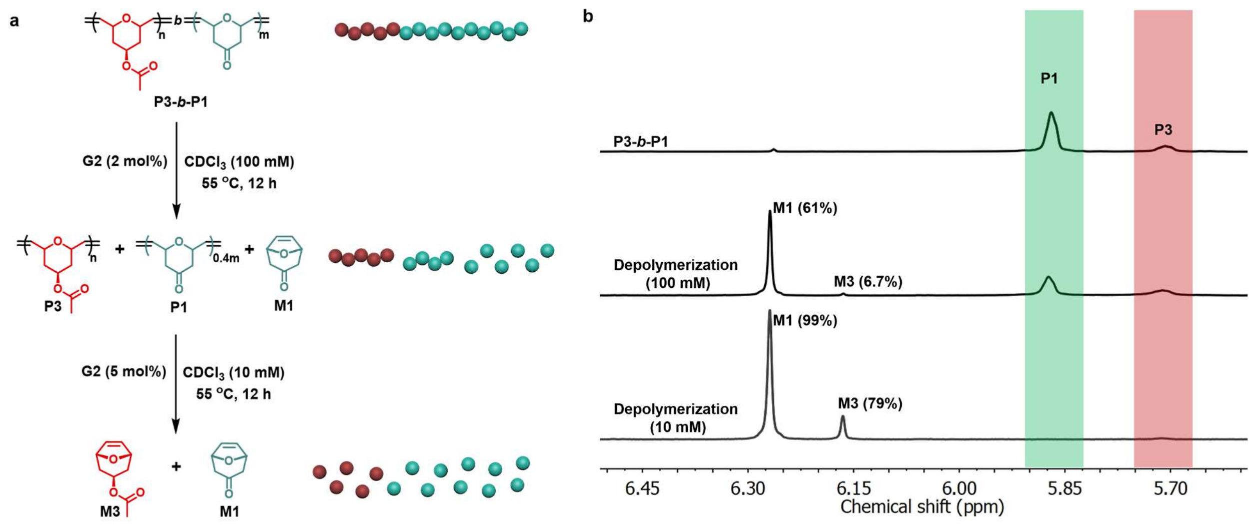

Figure S57. (a) Schematic illustration of the sequential depolymerization of diblock copolymer P3-*b*-P1. (b) Partial ¹H NMR spectra of P3-*b*-P1 (top), the products after the initial depolymerization at 100 mM olefins (middle), and the products after the second depolymerization at 10 mM olefins (bottom).

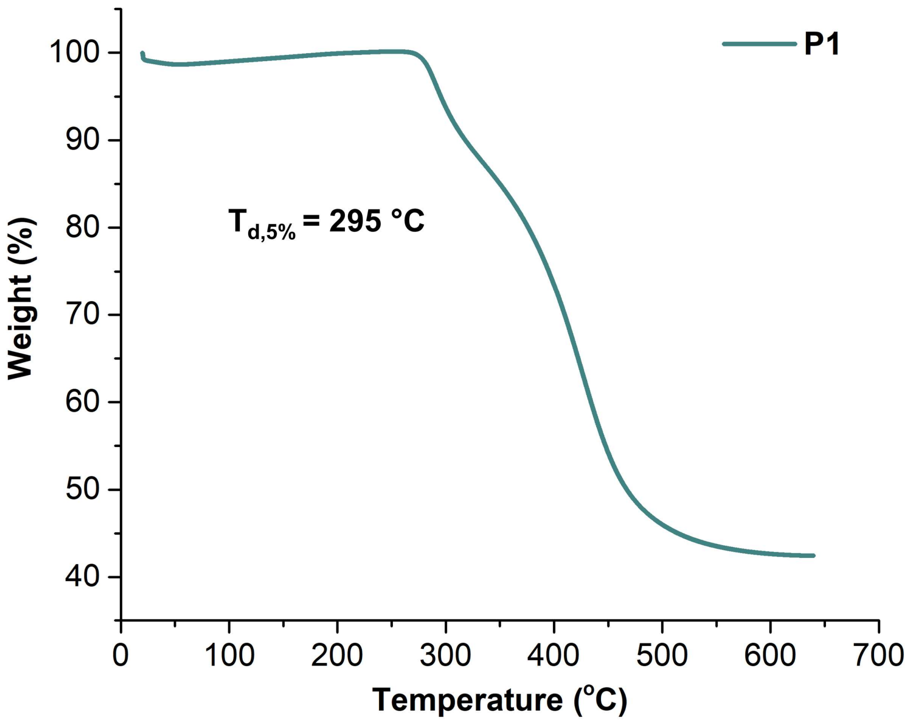

Figure S58. Thermogravimetric analysis of P1.

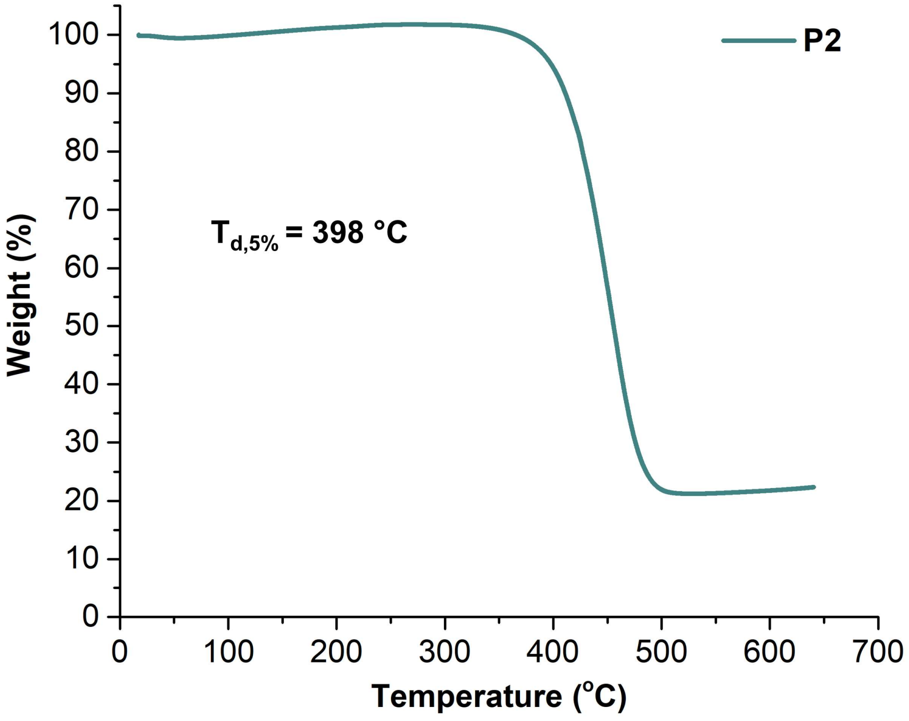

Figure S59. Thermogravimetric analysis of P2.

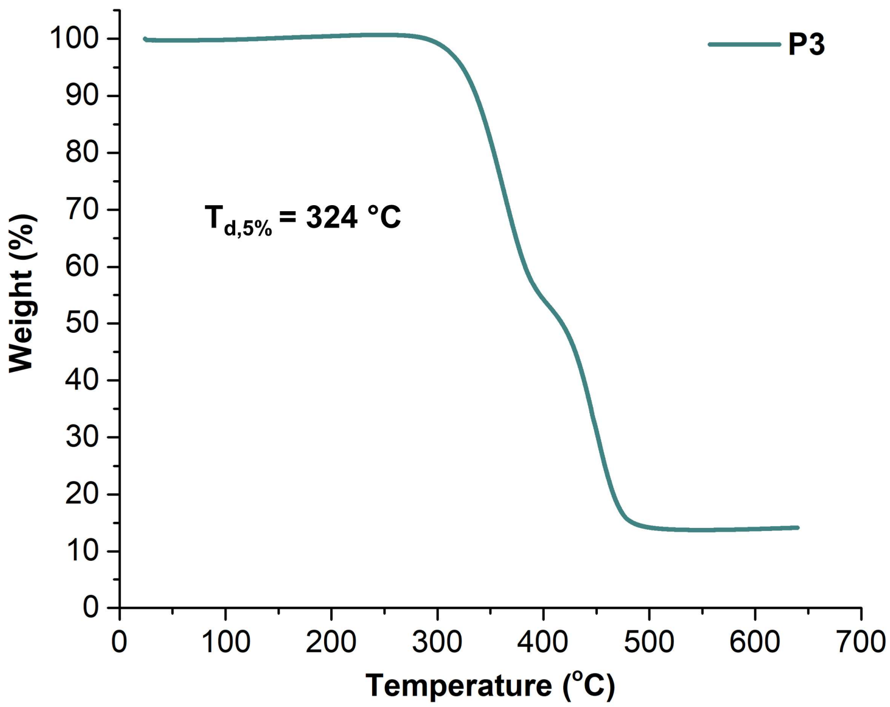

Figure S60. Thermogravimetric analysis of P3.

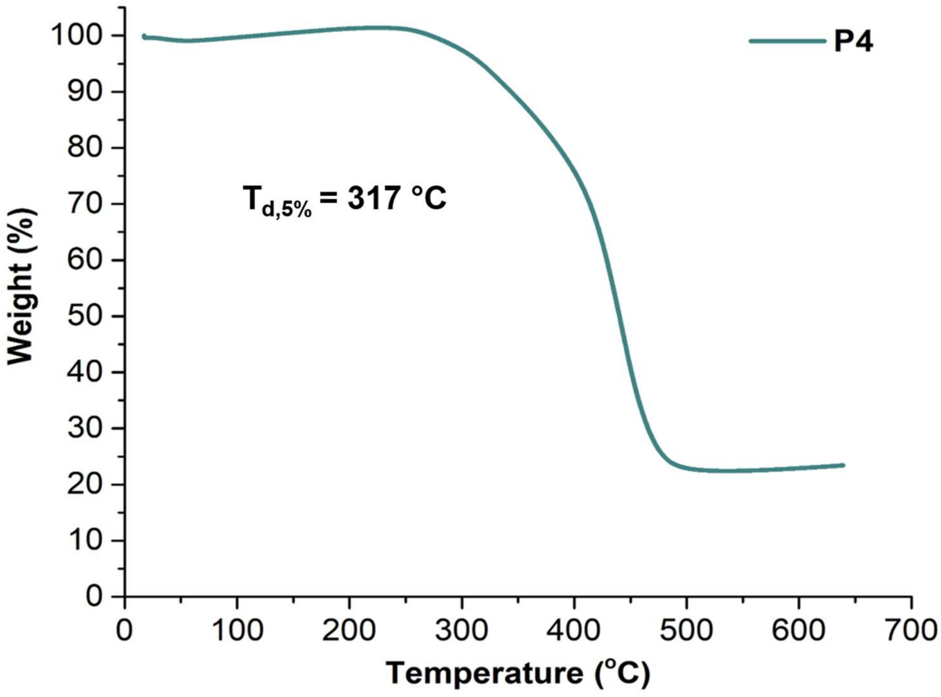

Figure S61. Thermogravimetric analysis of P4.

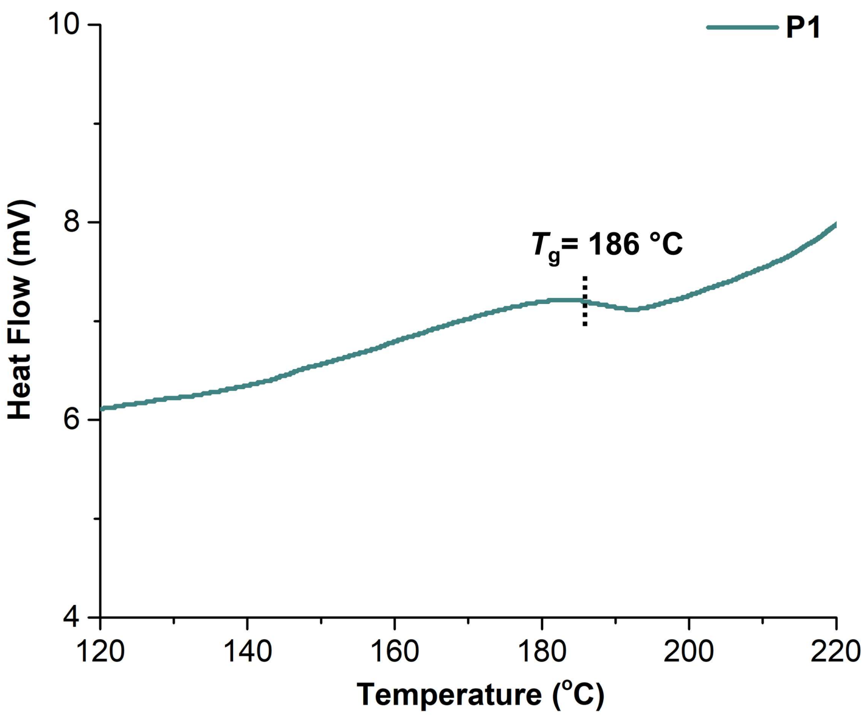

Figure S62. Differential scanning calorimetry thermogram of P1.

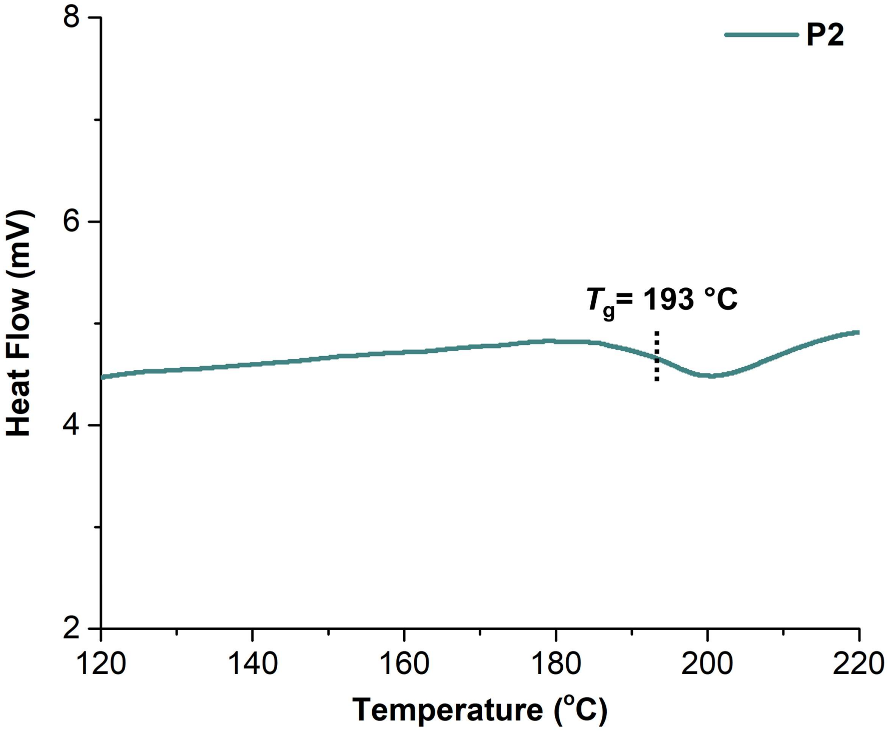

Figure S63. Differential scanning calorimetry thermogram of P2.

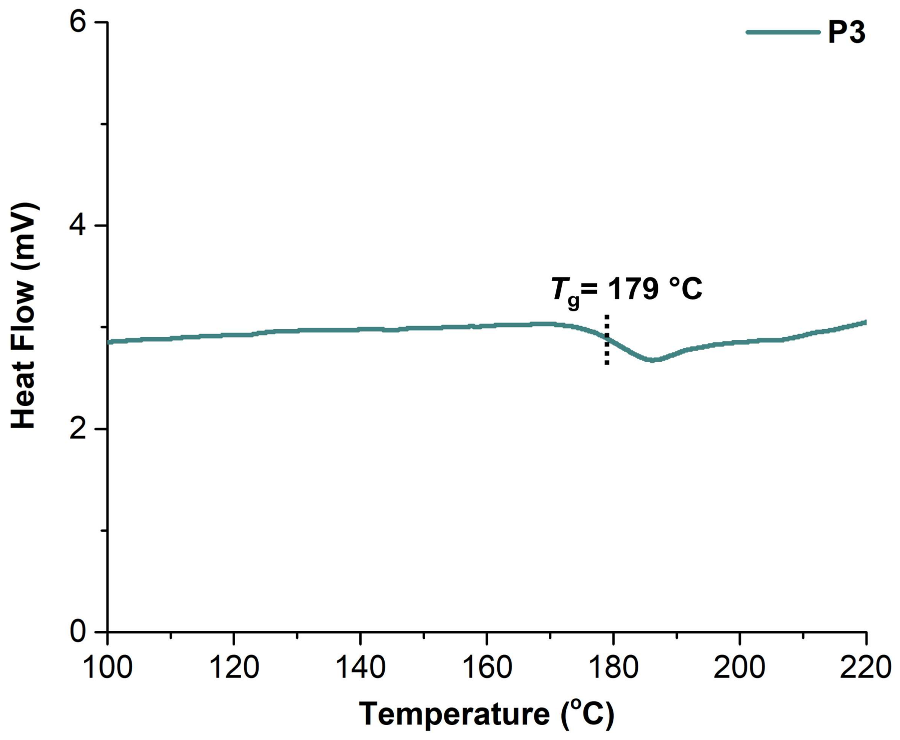

Figure S64. Differential scanning calorimetry thermogram of P3.

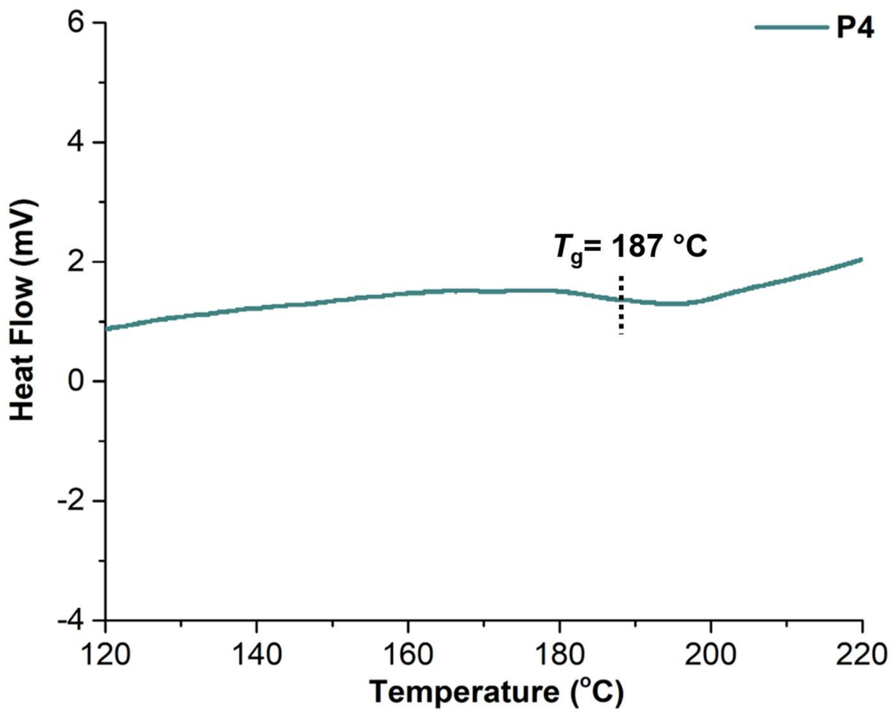

Figure S65. Differential scanning calorimetry thermogram of P4.

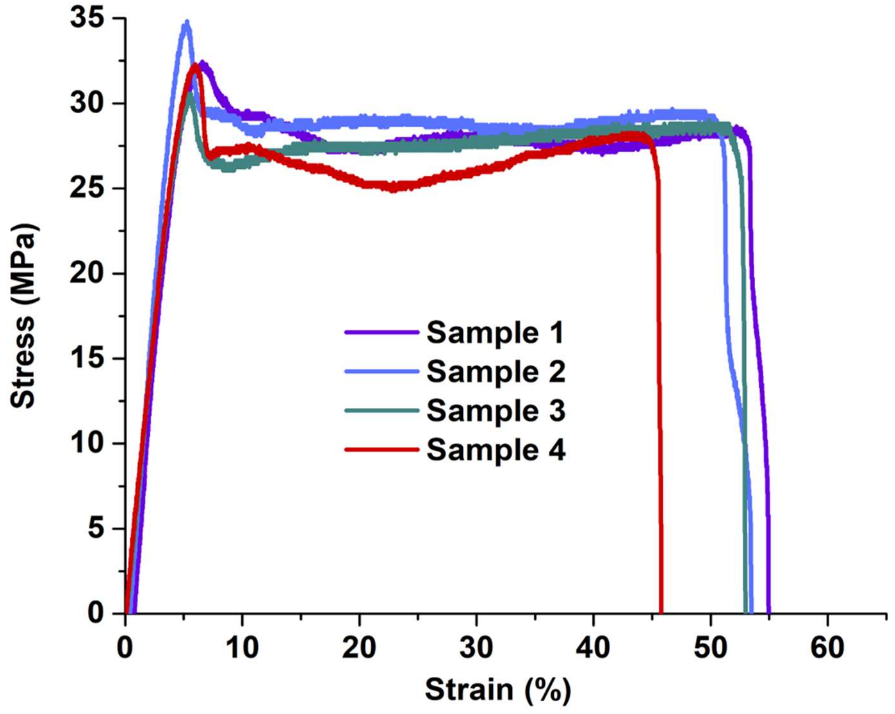

Figure S66. Stress–strain curves obtained for the P4 samples.

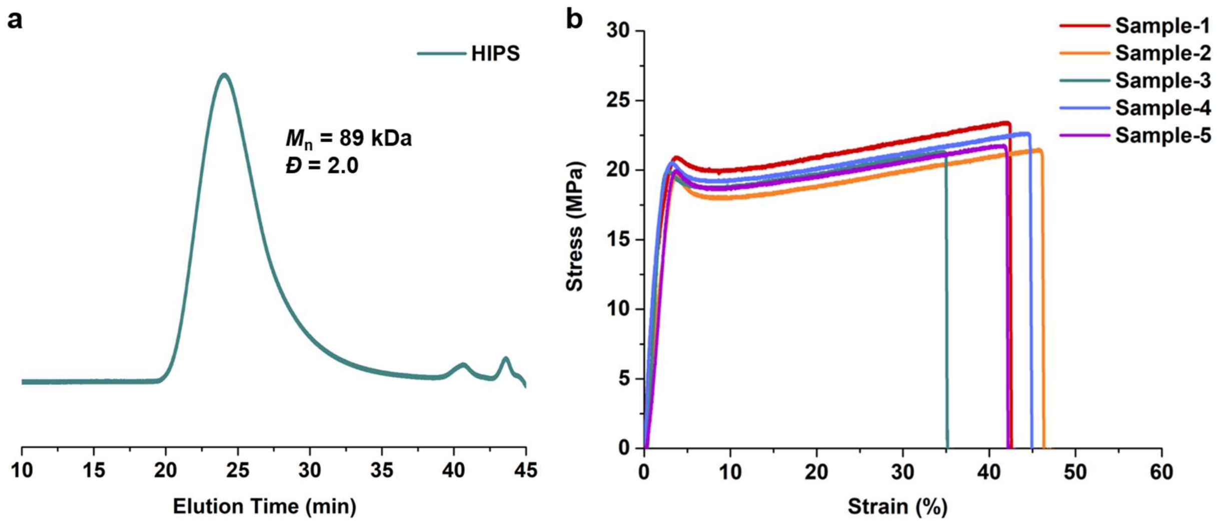

Figure S67. (a) SEC trace of HIPS. THF was used as the mobile phase. (b) Stress–strain curves obtained for the high-impact polystyrene samples.

Table S15. Computed RSE values of monomers

Monomer	RSE Value (kcal/mol)
M1	9.6
M2	10.8
M3	11.9
M4	10.2
Oxabicyclo[3.2.1]octene	12.1
Bicyclo[3.2.1]octene	13.7
Norbornenone	17.4
Norbornene	16.7
Oxanorbornene	18.7
Thianorbornene	20.4
Cyclobutene	30.1
Cyclopropene	54.4

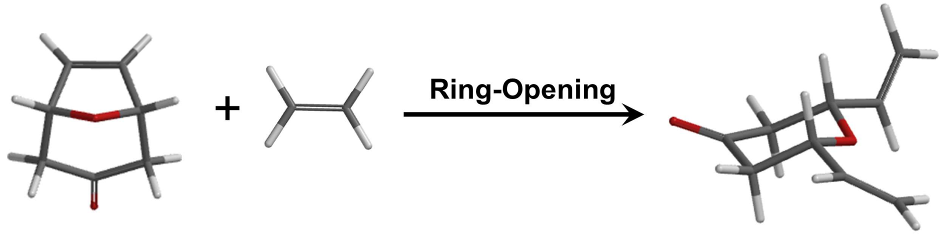

M1 **Ethylene** **Product**

**Chemical Structure**	**Enthalpy (Hartree)**
M1	-421.817640
Ethylene	-78.5322597
Ring-Opened Product	-500.365150

RSE = -ΔH = 500.365150 - 500.349899 = 0.015251 Hartree = 9.57 kcal/mol

Figure S68. DFT calculation of ethenolysis ring strain energy (RSE) of M1. A B3LYP/6-31G* level of theory was applied for the geometry optimization and energy calculation of conformers in vacuum. The RSE was estimated as the enthalpy difference between the ring-opened product and the total enthalpy of the isolated reactants (M1 + ethylene), using their lowest-energy conformers.

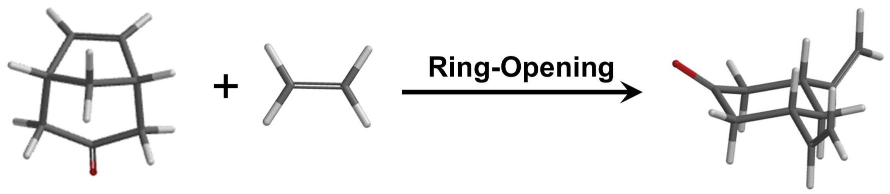

M2 **Ethylene** **Product**

Chemical Structure	Enthalpy (Hartree)
M2	-385.897259
Ethylene	-78.5322597
Ring-Opened Product	-464.446771

RSE = -ΔH = 464.446771 - 464.4295187 = 0.017252 Hartree = 10.82 kcal/mol

Figure S69. DFT calculation of ethenolysis ring strain energy (RSE) of M2. A B3LYP/6-31G* level of theory was applied for the geometry optimization and energy calculation of conformers in vacuum. The RSE was estimated as the enthalpy difference between the ring-opened product and the total enthalpy of the isolated reactants (M2 + ethylene), using their lowest-energy conformers.

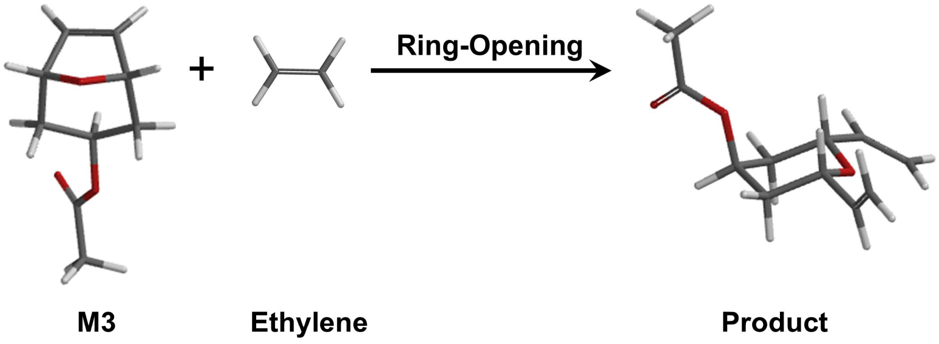

Chemical Structure	Enthalpy (Hartree)
M3	-575.619913
Ethylene	-78.5322597
Ring-Opened Product	-654.171209

RSE = -ΔH = 654.171209 – 654.152173 = 0.019036 Hartree = 11.94 kcal/mol

Figure S70. DFT calculation of ethenolysis ring strain energy (RSE) of M3. A B3LYP/6-31G* level of theory was applied for the geometry optimization and energy calculation of conformers in vacuum. The RSE was estimated as the enthalpy difference between the ring-opened product and the total enthalpy of the isolated reactants (M3 + ethylene), using their lowest-energy conformers.

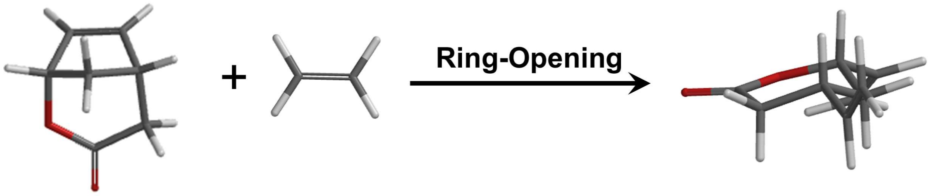

M4 **Ethylene** **Product**

Chemical Structure	Enthalpy (Hartree)
M4	-421.836727
Ethylene	-78.5322597
Ring-Opened Product	-500.385302

RSE = -ΔH = 500.385302 - 500.368987 = 0.016315 Hartree = 10.23 kcal/mol

Figure S71. DFT calculation of ethenolysis ring strain energy (RSE) of M4. A B3LYP/6-31G* level of theory was applied for the geometry optimization and energy calculation of conformers in vacuum. The RSE was estimated as the enthalpy difference between the ring-opened product and the total enthalpy of the isolated reactants (M4 + ethylene), using their lowest-energy conformers.

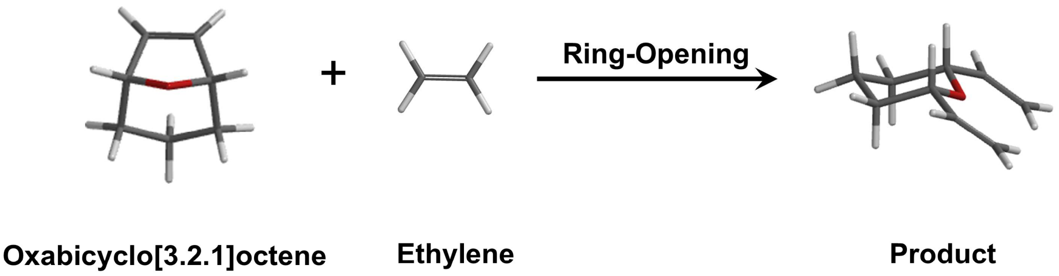

Chemical Structure	Enthalpy (Hartree)
Oxabicyclo[3.2.1]octene	-347.784707
Ethylene	-78.5322597
Ring-Opened Product	-426.336271

RSE = -ΔH = 426.336271 - 426.316967 = 0.019304 Hartree = 12.11 kcal/mol

Figure S72. DFT calculation of ethenolysis ring strain energy (RSE) of oxabicyclo[3.2.1]octene. A B3LYP/6-31G* level of theory was applied for the geometry optimization and energy calculation of conformers in vacuum. The RSE was estimated as the enthalpy difference between the ring-opened product and the total enthalpy of the isolated reactants (monomer + ethylene), using their lowest-energy conformers.

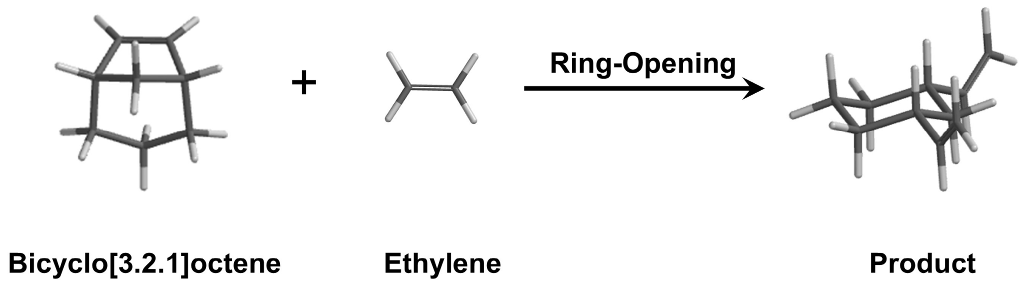

Chemical Structure	Enthalpy (Hartree)
Bicyclo[3.2.1]octene	-311.863207
Ethylene	-78.5322597
Ring-Opened Product	-390.417225

RSE = -ΔH = 390.417225 - 390.395467 = 0.021758 Hartree = 13.65 kcal/mol

Figure S73. DFT calculation of ethenolysis ring strain energy (RSE) of bicyclo[3.2.1]octene. A B3LYP/6-31G* level of theory was applied for the geometry optimization and energy calculation of conformers in vacuum. The RSE was estimated as the enthalpy difference between the ring-opened product and the total enthalpy of the isolated reactants (monomer + ethylene), using their lowest-energy conformers.

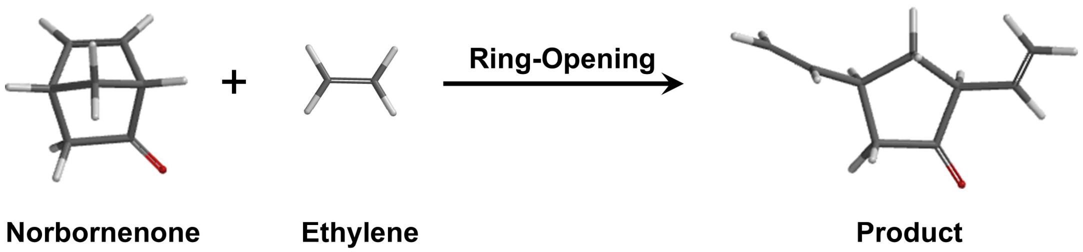

Chemical Structure	Enthalpy (Hartree)
Norbornenone	-346.599635
Ethylene	-78.5322597
Ring-Opened Product	-425.159637

RSE = -ΔH = 425.159637 – 425.131895 = 0.027742 Hartree = 17.41 kcal/mol

Figure S74. DFT calculation of ethenolysis ring strain energy (RSE) of norbornenone. A B3LYP/6-31G* level of theory was applied for the geometry optimization and energy calculation of conformers in vacuum. The RSE was estimated as the enthalpy difference between the ring-opened product and the total enthalpy of the isolated reactants (monomer + ethylene), using their lowest-energy conformers.

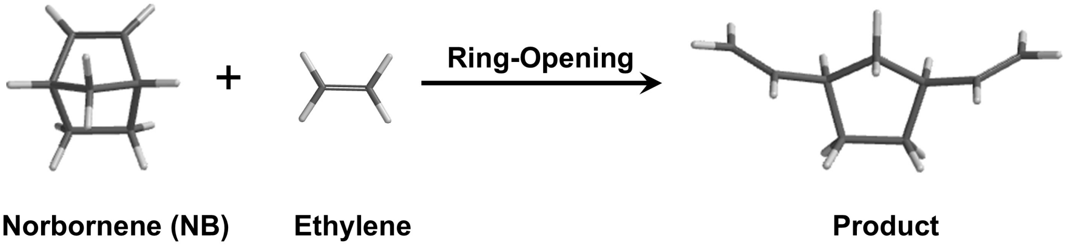

Chemical Structure	Enthalpy (Hartree)
NB	-272.567617
Ethylene	-78.5322597
Ring-Opened Product	-351.126493

RSE = -ΔH = 351.126493 - 351.099877 = 0.026616 Hartree = 16.70 kcal/mol

Figure S75. DFT calculation of ethenolysis ring strain energy (RSE) of norbornene. A B3LYP/6-31G* level of theory was applied for the geometry optimization and energy calculation of conformers in vacuum. The RSE was estimated as the enthalpy difference between the ring-opened product and the total enthalpy of the isolated reactants (monomer + ethylene), using their lowest-energy conformers.

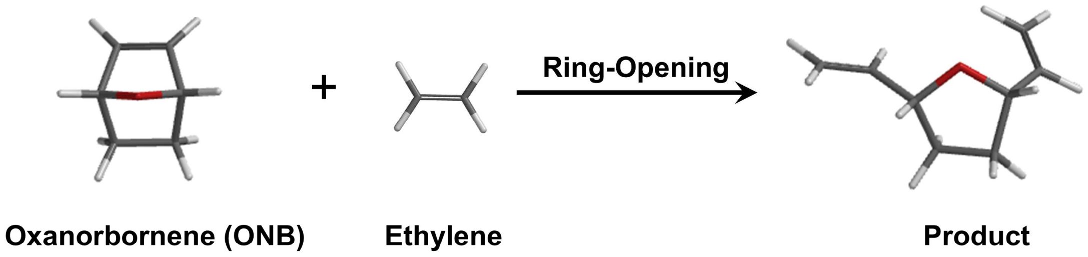

Chemical Structure	Enthalpy (Hartree)
ONB	-308.486453
Ethylene	-78.5322597
Ring-Opened Product	-387.048434

RSE = -ΔH = 387.048434 - 387.0187127 = 0.029721 Hartree = 18.65 kcal/mol

Figure S76. DFT calculation of ethenolysis ring strain energy (RSE) of oxanorbornene. A B3LYP/6-31G* level of theory was applied for the geometry optimization and energy calculation of conformers in vacuum. The RSE was estimated as the enthalpy difference between the ring-opened product and the total enthalpy of the isolated reactants (monomer + ethylene), using their lowest-energy conformers.

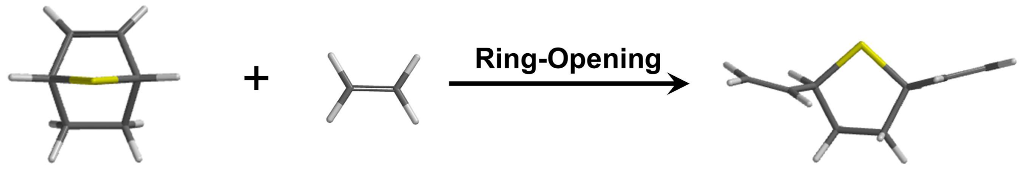

Thianorbornene (TNB) **Ethylene** **Product**

**Chemical Structure**	**Enthalpy (Hartree)**
TNB	-631.467363
Ethylene	-78.5322597
Ring-Opened Product	-710.032135

RSE = -ΔH = 710.032135 - 709.9996227 = 0.032512 Hartree = 20.40 kcal/mol

Figure S77. DFT calculation of ethenolysis ring strain energy (RSE) of thianorbornene. A B3LYP/6-31G* level of theory was applied for the geometry optimization and energy calculation of conformers in vacuum. The RSE was estimated as the enthalpy difference between the ring-opened product and the total enthalpy of the isolated reactants (monomer + ethylene), using their lowest-energy conformers.

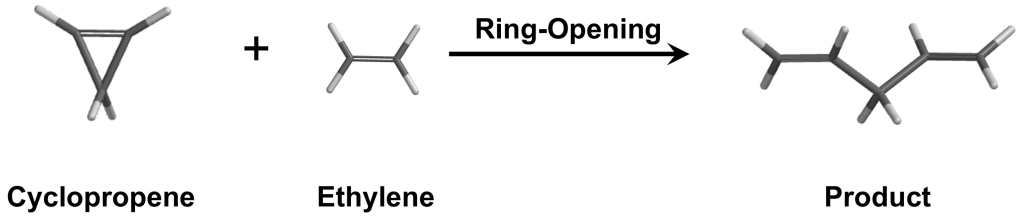

Chemical Structure	Enthalpy (Hartree)
Cyclopropene	-116.558484
Ethylene	-78.5322597
Ring-Opened Product	-195.177493

RSE = -ΔH = 195.177493 - 195.0907437 = 0.0867493 Hartree = 54.44 kcal/mol

Figure S78. DFT calculation of ethenolysis ring strain energy (RSE) of cyclopropene. A B3LYP/6-31G* level of theory was applied for the geometry optimization and energy calculation of conformers in vacuum. The RSE was estimated as the enthalpy difference between the ring-opened product and the total enthalpy of the isolated reactants (monomer + ethylene), using their lowest-energy conformers.

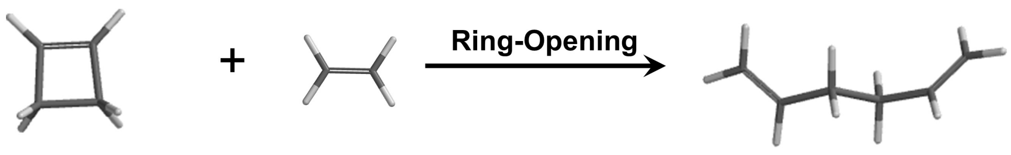

Cyclobutene **Ethylene** **Product**

Chemical Structure	Enthalpy (Hartree)
Cyclobutene	-155.881577
Ethylene	-78.5322597
Ring-Opened Product	-234.461860

RSE = -ΔH = 234.461840 - 234.4138367 = 0.0480033 Hartree = 30.12 kcal/mol

Figure S79. DFT calculation of ethenolysis ring strain energy (RSE) of cyclobutene. A B3LYP/6-31G* level of theory was applied for the geometry optimization and energy calculation of conformers in vacuum. The RSE was estimated as the enthalpy difference between the ring-opened product and the total enthalpy of the isolated reactants (monomer + ethylene), using their lowest-energy conformers.

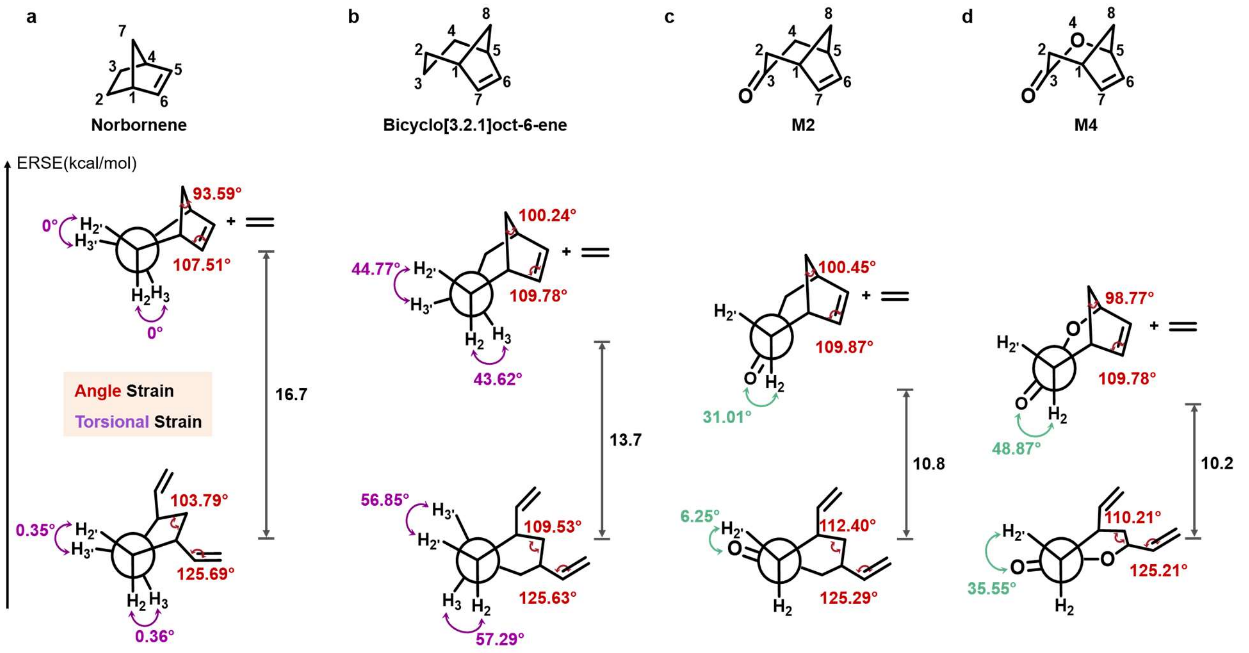

Figure S80. Conformational analysis of the monomers and the corresponding ring-opened products formed via ethylenolysis. Bicyclo[3.2.1]oct-6-ene (b), M2 (c), and M4 (d) exhibit substantially lower angle strain compared to norbornene (a). The geometries and energies of the monomers and their ring-opened products were optimized at the B3LYP/6-31G(d) level of theory in the gas phase.

6. Optimized Geometries of Monomers and Their Ring-Opened Structures from DFT Calculations

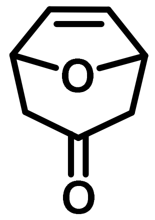

M1

C	1.296838	-1.055999	-0.666668
H	1.485155	-1.892143	-1.330926
C	0.845998	0.322186	-1.125868
H	1.369440	0.716035	-2.001787
C	-0.690550	0.358961	-1.308079
H	-0.985494	1.382606	-1.578467
H	-1.047170	-0.310976	-2.097429
C	-1.402338	-0.012338	0.000000
C	-0.690550	0.358961	1.308079
H	-0.985494	1.382606	1.578467
H	-1.047170	-0.310976	2.097429
C	0.845998	0.322186	1.125868
H	1.369440	0.716035	2.001787
C	1.296838	-1.055999	0.666668
H	1.485155	-1.892143	1.330926
O	1.155514	1.154910	0.000000
O	-2.487673	-0.557260	0.000000

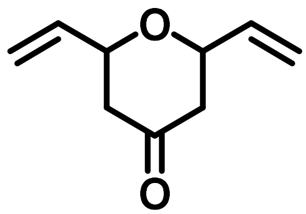

Ring-Opened M1

O	-0.134440	-0.892378	-0.068503
C	-1.364283	1.208642	-0.217547
C	1.166160	1.125390	-0.508952
C	-0.046552	1.960139	-0.127033
C	1.122033	-0.261152	0.183756
C	-1.240829	-0.180213	0.464781
O	0.034417	3.115491	0.242117
H	-2.158051	1.811718	0.233219
H	-1.608023	1.039067	-1.275036
H	2.080858	1.666492	-0.252518
H	1.149470	0.962755	-1.596019
H	-1.073785	-0.011254	1.545296
H	1.247229	-0.118527	1.269841
C	-2.495903	-0.991293	0.295241
H	-3.394070	-0.515594	0.688760
C	2.197065	-1.174763	-0.331244
H	2.063208	-1.507002	-1.360597
C	-2.564157	-2.188029	-0.284735
H	-3.509921	-2.714290	-0.377836
H	-1.678424	-2.677825	-0.675694
C	3.265261	-1.549194	0.370405
H	4.035193	-2.185463	-0.056928
H	3.413717	-1.232142	1.400567

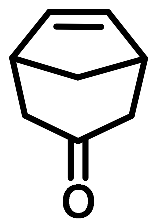

M2

C	1.276064	-1.091212	0.668943
C	1.276064	-1.091212	-0.668943
C	0.843875	0.275573	1.187072
C	0.843875	0.275573	-1.187072
C	1.250712	1.176178	0.000000
C	-0.699208	0.308599	1.320799
C	-0.699208	0.308599	-1.320799
C	-1.422725	0.002878	0.000000
O	-2.554658	-0.439598	0.000000
H	1.454123	-1.949406	1.310637
H	1.454123	-1.949406	-1.310637
H	1.311788	0.560056	2.135760
H	1.311788	0.560056	-2.135760
H	0.746303	2.150158	0.000000
H	2.333146	1.344639	0.000000
H	-1.070336	-0.402040	2.066956
H	-1.025010	1.307462	1.650320
H	-1.070336	-0.402040	-2.066956
H	-1.025010	1.307462	-1.650320

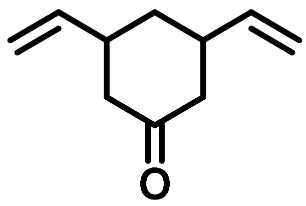

Ring-Opened M2

C	-0.329132	-1.043995	0.000000
C	-0.366311	1.146965	-1.286140
C	-0.366311	1.146965	1.286140
C	-0.008216	1.878176	0.000000
C	0.134493	-0.320899	1.282999
C	0.134493	-0.320899	-1.282999
C	-0.324663	-1.044301	-2.522298
C	-0.324663	-1.044301	2.522298
C	0.476690	-1.554952	3.457370
C	0.476690	-1.554952	-3.457370
O	0.532758	2.967503	0.000000
H	-1.427852	-1.119125	0.000000
H	0.053937	-2.070967	0.000000
H	-1.463778	1.144980	-1.382404
H	0.040242	1.705391	-2.133851
H	-1.463778	1.144980	1.382404
H	0.040242	1.705391	2.133851
H	1.233969	-0.302485	1.283678
H	1.233969	-0.302485	-1.283678
H	-1.407052	-1.139431	-2.636041
H	-1.407052	-1.139431	2.636041
H	0.083264	-2.066700	4.331618
H	1.560068	-1.480134	3.385545
H	1.560068	-1.480134	-3.385545
H	0.083264	-2.066700	-4.331618

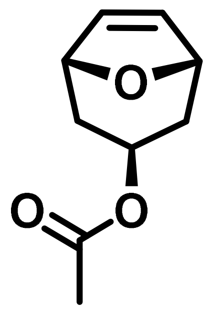

M3

C	2.289241	1.217529	-0.556720
H	2.367732	2.225840	-0.948643
C	1.822318	0.872447	0.852366
H	2.231645	1.506646	1.644365
C	0.280427	0.783002	0.934602
H	0.020070	0.384406	1.921631
H	-0.192680	1.765230	0.826429
C	-0.253912	-0.168875	-0.154096
C	0.609302	-1.430627	-0.328750
H	0.394072	-2.133821	0.483249
H	0.361834	-1.926832	-1.275587
C	2.110009	-1.060740	-0.252273
H	2.744964	-1.948244	-0.330829
C	2.460429	0.069568	-1.212776
H	2.711162	-0.067194	-2.259234
O	2.327772	-0.459770	1.031460
O	-1.592580	-0.620048	0.185017
H	-0.323515	0.373077	-1.098031
C	-2.622097	0.186430	-0.165919
C	-3.941577	-0.433443	0.234281
H	-4.066972	-1.403100	-0.259165
H	-4.756869	0.233633	-0.047210
H	-3.961513	-0.613100	1.313789
O	-2.492039	1.253783	-0.725859

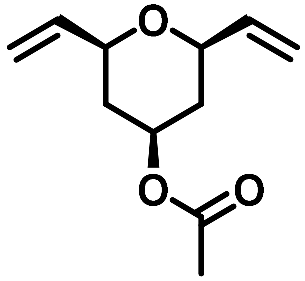

Ring-Opened M3

C	1.350780	2.634398	0.488123
C	0.737911	1.270007	0.328438
H	0.213213	1.007551	1.265609
C	-0.290567	1.238105	-0.815920
H	-1.109538	1.940359	-0.629074
H	0.207853	1.550338	-1.741946
C	-0.848314	-0.173807	-1.013030
C	0.281115	-1.198134	-1.107913
H	-0.127486	-2.214485	-1.106361
H	0.802679	-1.048755	-2.061987
C	1.294410	-1.036185	0.042783
H	0.797103	-1.285376	0.993426
C	2.495800	-1.917889	-0.145337
O	1.768268	0.314310	0.094166
O	-1.657954	-0.559435	0.137267
H	-1.492508	-0.207786	-1.895274
C	2.773446	-2.988082	0.597396
H	3.639640	-3.612896	0.397163
H	2.146214	-3.284397	1.436019
C	2.652016	2.906729	0.411213
H	3.023498	3.920793	0.529129
H	3.380191	2.122375	0.233514
H	0.629779	3.431756	0.670254
H	3.146114	-1.637813	-0.974348
C	-2.951832	-0.155911	0.142195

C	-3.660251	-0.651805	1.381861
H	-3.633822	-1.745935	1.418039
H	-4.694512	-0.306351	1.371534
H	-3.153006	-0.284027	2.279848
O	-3.458127	0.506387	-0.736982

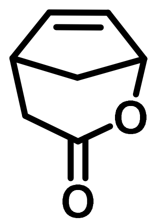

M4

C	1.466663	-0.571019	-0.993179
C	1.378372	0.762857	-0.949730
C	0.861959	-1.168846	0.269990
C	0.709322	1.158200	0.354679
C	1.070458	-0.013430	1.271473
C	-0.665974	-1.309182	0.077070
O	-0.744197	1.193646	0.161262
C	-1.412053	0.027874	-0.068486
O	-2.588182	0.067465	-0.335243
H	1.814717	-1.166406	-1.832325
H	1.629184	1.464916	-1.737472
H	1.305921	-2.122051	0.573332
H	0.949500	2.151578	0.735109
H	0.430748	-0.064755	2.158921
H	2.116190	0.056696	1.586925
H	-0.921529	-1.910045	-0.802258
H	-1.118174	-1.817548	0.938717

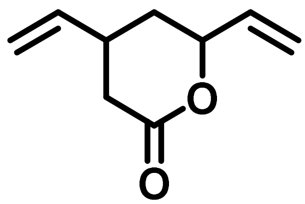

Ring-Opened M4

C	0.247948	-1.131457	0.174906
O	-1.391311	0.672040	-0.257352
C	0.963761	1.245473	0.284784
C	-0.468615	1.648094	-0.042967
C	1.389300	-0.161857	-0.171556
C	-1.042365	-0.702569	-0.528996
C	2.696227	-0.570463	0.455427
C	-2.212490	-1.573404	-0.158367
C	3.809987	-0.868179	-0.213859
C	-3.331480	-1.161389	0.433917
O	-0.830476	2.799349	-0.042326
H	0.080539	-1.153389	1.260164
H	0.498078	-2.152165	-0.136506
H	1.044405	1.311559	1.379878
H	1.619800	2.022677	-0.116321
H	1.515461	-0.157995	-1.263908
H	-0.879801	-0.775965	-1.616562
H	2.699192	-0.617898	1.546674
H	-2.077052	-2.624933	-0.412161
H	3.853184	-0.830809	-1.300740
H	4.723537	-1.159063	0.297382
H	-4.126198	-1.861091	0.676073
H	-3.490491	-0.117537	0.683713

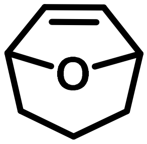

Oxabicyclo[3.2.1]octene

C	1.304321	0.774037	0.666543
C	1.304321	0.774037	-0.666543
C	0.470601	-0.415884	1.125178
C	0.470601	-0.415884	-1.125178
O	0.551373	-1.306266	0.000000
C	-1.018175	-0.036962	1.289827
C	-1.551604	0.633212	0.000000
C	-1.018175	-0.036962	-1.289827
H	1.732101	1.518420	1.330025
H	1.732101	1.518420	-1.330025
H	0.859738	-0.939214	2.004698
H	0.859738	-0.939214	-2.004698
H	-1.567186	-0.964208	1.494886
H	-1.165774	0.627708	2.151453
H	-2.648030	0.624199	0.000000
H	-1.252064	1.686945	0.000000
H	-1.567186	-0.964208	-1.494886
H	-1.165774	0.627708	-2.151453

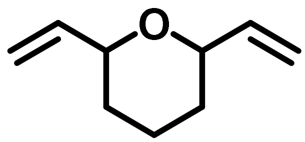

Ring-Opened Oxabicyclo[3.2.1]octene

O	0.130716	-0.549706	0.000000
C	-0.343955	1.482380	-1.259300
C	-0.343955	1.482380	1.259300
C	-0.093129	2.321762	0.000000
C	0.448649	0.162668	1.190665
C	0.448649	0.162668	-1.190665
C	0.192193	-0.714867	-2.385416
C	0.192193	-0.714867	2.385416
C	-0.277280	-1.960528	-2.348051
C	-0.277280	-1.960528	2.348051
H	-1.409889	1.236126	-1.346818
H	-0.060777	2.038426	-2.162272
H	-1.409889	1.236126	1.346818
H	-0.060777	2.038426	2.162272
H	-0.728948	3.215103	0.000000
H	0.948645	2.675277	0.000000
H	1.527228	0.417510	1.171984
H	1.527228	0.417510	-1.171984
H	0.419297	-0.243584	-3.342199
H	0.419297	-0.243584	3.342199
H	-0.505177	-2.449666	-1.407060
H	-0.441646	-2.528382	-3.259657
H	-0.441646	-2.528382	3.259657
H	-0.505177	-2.449666	1.407060

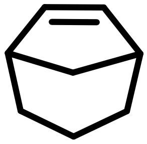

Bicyclo[3.2.1]octene

C	1.227221	-0.876383	0.669037
C	1.227221	-0.876383	-0.669037
C	0.490577	0.356391	1.185485
C	0.490577	0.356391	-1.185485
C	0.707743	1.322863	0.000000
C	-1.033218	0.073633	1.296300
C	-1.593579	-0.558209	0.000000
C	-1.033218	0.073633	-1.296300
H	1.605141	-1.668833	1.309796
H	1.605141	-1.668833	-1.309796
H	0.877303	0.738216	2.137788
H	0.877303	0.738216	-2.137788
H	0.012867	2.171815	0.000000
H	1.732794	1.711213	0.000000
H	-1.246127	-0.589330	2.145098
H	-1.542251	1.024591	1.506143
H	-1.344516	-1.624747	0.000000
H	-2.689224	-0.499185	0.000000
H	-1.246127	-0.589330	-2.145098
H	-1.542251	1.024591	-1.506143

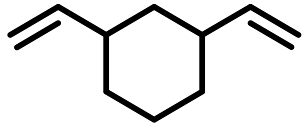

Ring-Opened Bicyclo[3.2.1]octene

C	-0.338754	-0.763050	0.000000
C	-0.170237	1.424402	-1.269734
C	-0.170237	1.424402	1.269734
C	0.345708	2.115174	0.000000
C	0.178277	-0.080626	1.286553
C	0.178277	-0.080626	-1.286553
C	-0.374682	-0.756393	-2.514044
C	-0.374682	-0.756393	2.514044
C	0.345388	-1.369388	3.454206
C	0.345388	-1.369388	-3.454206
H	-1.440181	-0.737751	0.000000
H	-0.052095	-1.821371	0.000000
H	-1.263460	1.537706	-1.329502
H	0.242349	1.906949	-2.164456
H	-1.263460	1.537706	1.329502
H	0.242349	1.906949	2.164456
H	0.053016	3.172970	0.000000
H	1.445809	2.096788	0.000000
H	1.274697	-0.175465	1.305706
H	1.274697	-0.175465	-1.305706
H	-1.462266	-0.724068	-2.615262
H	-1.462266	-0.724068	2.615262
H	-0.118756	-1.837727	4.318348
H	1.430827	-1.427055	3.395973
H	1.430827	-1.427055	-3.395973
H	-0.118756	-1.837727	-4.318348

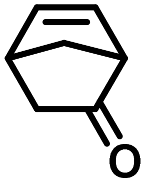

Norbornenone

C	1.030743	0.953267	0.023715
C	-1.174731	-0.003284	-0.042329
C	0.804123	-1.288735	-0.526602
C	-0.117119	-0.910917	0.634300
C	1.488849	-0.191526	-0.878855
C	-0.423163	1.294385	-0.418728
C	0.749065	0.165981	1.332462
O	-2.342230	-0.252838	-0.231796
H	1.701284	1.811919	0.100142
H	0.788691	-2.251086	-1.026423
H	-0.541845	-1.723388	1.223628
H	2.165187	-0.083606	-1.720663
H	-0.529262	1.527156	-1.481793
H	-0.850956	2.127907	0.152873
H	0.204214	0.753983	2.081818
H	1.653921	-0.255210	1.781008

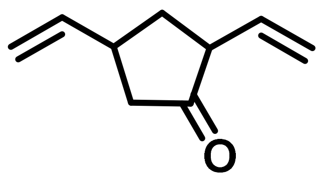

Ring-Opened Norbornenone

C	0.133381	-0.961581	-0.197820
C	-1.110662	-0.133606	-0.554120
C	-0.785705	1.280670	-0.019605
C	0.696593	1.330950	0.352306
C	1.311562	0.050986	-0.256434
O	-1.579170	2.189323	0.079737
C	-2.462063	-0.620056	-0.106520
C	2.554067	-0.426893	0.437098
C	3.739804	-0.610039	-0.144733
C	-2.740631	-1.836685	0.361317
H	0.037284	-1.350897	0.824677
H	0.295045	-1.816678	-0.860747
H	-1.141296	0.000843	-1.651316
H	0.758320	1.302085	1.450974
H	1.163094	2.266625	0.030576
H	1.548399	0.244232	-1.312159
H	-3.250570	0.127634	-0.183523
H	2.444016	-0.637068	1.503450
H	4.602782	-0.964204	0.412403
H	3.892676	-0.411749	-1.203810
H	-1.985972	-2.613845	0.455503
H	-3.748481	-2.104022	0.667147

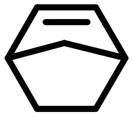

Norbornene

C	0.087962	-0.322205	1.128300
C	-1.191562	0.516542	-0.780555
C	1.280004	0.506750	-0.670327
C	0.087962	-0.322205	-1.128300
C	1.280004	0.506750	0.670327
C	-1.191562	0.516542	0.780555
C	0.040416	-1.380937	0.000000
H	0.119300	-0.688610	2.157624
H	-1.147622	1.523241	-1.206205
H	-2.088131	0.026508	-1.177671
H	1.920965	1.085540	-1.328752
H	0.119300	-0.688610	-2.157624
H	1.920965	1.085540	1.328752
H	-1.147622	1.523241	1.206205
H	-2.088131	0.026508	1.177671
H	-0.880237	-1.977695	0.000000
H	0.911866	-2.043091	0.000000

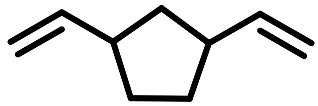

Ring-Opened Norbornene

C	0.000160	-0.800432	0.230689
C	-1.215235	0.014532	-0.263219
C	-0.777631	1.487741	-0.011764
C	0.777115	1.487387	-0.006266
C	1.215386	0.015503	-0.261912
C	-2.512670	-0.353352	0.396003
C	2.513162	-0.353723	0.395860
C	3.604413	-0.797033	-0.230077
C	-3.604466	-0.797435	-0.228432
H	-0.000260	-0.841753	1.330590
H	0.000720	-1.832843	-0.134577
H	-1.314394	-0.145834	-1.345959
H	-1.164650	1.828053	0.956482
H	-1.199547	2.158247	-0.767532
H	1.157031	1.821446	0.966999
H	1.205053	2.162161	-0.754807
H	1.314002	-0.142160	-1.345139
H	-2.532980	-0.237150	1.482360
H	2.534258	-0.239309	1.482391
H	4.513119	-1.048882	0.310263
H	3.631044	-0.925452	-1.310601
H	-3.631899	-0.927627	-1.308724
H	-4.512892	-1.048024	0.312962

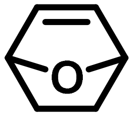

Oxanorbornene

C	0.081780	-0.366166	1.069005
C	-1.187593	0.503737	-0.778841
C	1.294870	0.467490	-0.668256
C	0.081780	-0.366166	-1.069005
C	1.294870	0.467490	0.668256
C	-1.187593	0.503737	0.778841
O	0.008759	-1.324853	0.000000
H	0.106150	-0.871055	2.035371
H	-1.124739	1.503773	-1.217015
H	-2.078820	0.007685	-1.174365
H	1.928029	1.028642	-1.346484
H	0.106150	-0.871055	-2.035371
H	1.928029	1.028642	1.346484
H	-1.124739	1.503773	1.217015
H	-2.078820	0.007685	1.174365

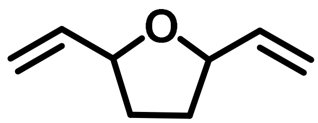

Ring-Opened Oxanorbornene

O	-0.073434	-0.212858	0.964342
C	1.004353	0.421232	0.245169
C	0.316666	1.227079	-0.870023
C	-0.990808	1.649981	-0.192245
C	-1.331373	0.399378	0.653570
C	1.963176	-0.623242	-0.244552
C	-2.254272	-0.561823	-0.058348
C	-1.950931	-1.814697	-0.393991
C	3.266281	-0.637912	0.033606
H	1.528893	1.105518	0.930835
H	0.111256	0.577515	-1.729169
H	0.928250	2.066528	-1.213795
H	-1.783125	1.914775	-0.898969
H	-0.818487	2.511963	0.462899
H	-1.803717	0.696183	1.602110
H	1.525317	-1.392733	-0.880754
H	-3.243334	-0.159006	-0.283640
H	-2.666180	-2.455910	-0.901568
H	-0.980972	-2.237001	-0.151163
H	3.720573	0.114208	0.675681
H	3.930447	-1.399149	-0.366324

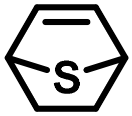

Thianorbornene

C	0.764007	1.270677	-0.668240
C	-0.042566	0.085625	1.183021
C	0.753327	-1.194487	-0.779455
C	0.753327	-1.194487	0.779455
C	-0.042566	0.085625	-1.183021
C	0.764007	1.270677	0.668240
S	-1.482521	0.028424	0.000000
H	1.335994	1.924656	-1.319181
H	-0.353262	0.124304	2.227413
H	0.260162	-2.087532	-1.173460
H	1.768661	-1.159712	-1.190776
H	0.260162	-2.087532	1.173460
H	1.768661	-1.159712	1.190776
H	-0.353262	0.124304	-2.227413
H	1.335994	1.924656	1.319181

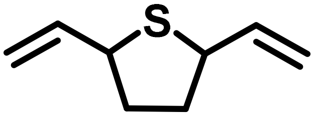

Ring-Opened Thianorbornene

S	-0.037307	-1.026014	-0.658821
C	-1.333837	0.263956	-0.258845
C	-0.538668	1.432756	0.347825
C	0.768137	1.553759	-0.439061
C	1.411874	0.150535	-0.515682
C	-2.383150	-0.302925	0.647445
C	2.269771	-0.191231	0.667722
C	3.536951	-0.600009	0.583861
C	-3.672132	-0.426486	0.326292
H	-1.792594	0.577179	-1.203202
H	-0.328657	1.217850	1.403344
H	-1.129608	2.355649	0.312905
H	1.460977	2.271093	0.016168
H	0.552021	1.902856	-1.456386
H	2.003149	0.050260	-1.431051
H	-2.030680	-0.627391	1.626883
H	1.798245	-0.093624	1.645843
H	4.122531	-0.833233	1.468855
H	4.035647	-0.724844	-0.375447
H	-4.050902	-0.125288	-0.648302
H	-4.396903	-0.836412	1.024200

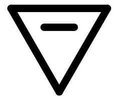

Cyclopropene

C	-0.647459	0.000000	-0.501261
C	0.647459	0.000000	-0.501261
C	0.000000	0.000000	0.861438
H	-1.581639	0.000000	-1.042762
H	1.581639	0.000000	-1.042762
H	0.000000	0.912787	1.466010
H	0.000000	-0.912787	1.466010

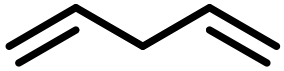

Ring-Opened Cyclopropene (1,4-Pentadiene)

C	-2.381168	0.147725	0.168572
C	-1.179221	-0.428980	0.183377
C	0.000000	-0.000000	-0.657488
C	1.179221	0.428980	0.183377
C	2.381168	-0.147725	0.168572
H	-3.192570	-0.203917	0.800473
H	-2.602442	0.993242	-0.480489
H	-0.996255	-1.270646	0.852605
H	0.310236	-0.827373	-1.311814
H	-0.310236	0.827373	-1.311814
H	0.996255	1.270646	0.852605
H	3.192570	0.203917	0.800473
H	2.602442	-0.993242	-0.480489

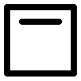

Cyclobutene

C	-0.670272	0.000000	0.814889
C	0.670272	0.000000	0.814889
C	-0.785944	0.000000	-0.699598
C	0.785944	0.000000	-0.699598
H	-1.420922	0.000000	1.601547
H	1.420922	0.000000	1.601547
H	-1.246211	-0.890034	-1.146648
H	-1.246211	0.890034	-1.146648
H	1.246211	-0.890034	-1.146648
H	1.246211	0.890034	-1.146648

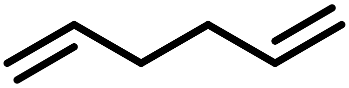

Ring-Opened Cyclobutene (1,5-hexadiene)

C	2.879371	0.011599	0.593038
C	1.933874	-0.114848	-0.338799
C	0.572136	0.521656	-0.289281
C	-0.572136	-0.521656	-0.289281
C	-1.933874	0.114848	-0.338799
C	-2.879371	-0.011599	0.593038
H	3.843433	-0.481994	0.501817
H	2.727122	0.618117	1.484085
H	2.133082	-0.737476	-1.213665
H	0.484215	1.157203	0.600127
H	0.441693	1.179699	-1.162114
H	-0.484215	-1.157203	0.600127
H	-0.441693	-1.179699	-1.162114
H	-2.133082	0.737476	-1.213665
H	-3.843433	0.481994	0.501817
H	-2.727122	-0.618117	1.484085

References

1. Lu, D. F.; Zhu, C. L.; Jia, Z. X.; Xu, H., Iron(II)-catalyzed intermolecular amino-oxygenation of olefins through the N-O bond cleavage of functionalized hydroxylamines. *J. Am. Chem. Soc.* **2014,** *136* (38), 13186-9.
2. Rudroff, F.; Bianchi, D. A.; Moran-Ramallal, R.; Iqbal, N.; Dreier, D.; Mihovilovic, M. D., Synthesis of tetrahydrofuran-based natural products and their carba analogs via stereoselective enzyme mediated Baeyer–Villiger oxidation. *Tetrahedron* **2016,** *72* (46), 7212-7221.
3. Treu, J.; Hoffmann, H. M. R., Chelation-Controlled Reduction of r-Methylated 8-Oxabicyclo[3.2.1]oct-6-en-3-ones with Samarium Diiodide. Diastereoselective Preparation of Secondary Alcohols. *J. Org. Chem.* **1997,** *62*, 4650-4652.
4. Stellmach, K. A.; Paul, M. K.; Su, Y.-L.; Ramprasad, R.; Engler, A. C.; Gutekunst, W. R., Improving the Accuracy of Ceiling Temperature Measurements: Best Practices and Common Pitfalls. *Macromolecules* **2025,** *58* (8), 3729-3741.
5. Ivin, K. J.; Mol, J. C., Olefin Metathesis and Metathesis Polymerization. *Academic Press: San Diego, CA* **1997**, 226.
6. Zhou, J.; Sathe, D.; Wang, J., Understanding the Structure-Polymerization Thermodynamics Relationships of Fused-Ring Cyclooctenes for Developing Chemically Recyclable Polymers. *J. Am. Chem. Soc.* **2022,** *144* (2), 928-934.
7. Su, H. W.; Zhou, J.; Yoon, S.; Wang, J., Evaluating Trans-Benzocyclobutene-Fused Cyclooctene as a Monomer for Chemically Recyclable Polymer. *Chem. Asian J.* **2023,** *18* (3), e202201133.
